# Association of Recent SARS-CoV-2 Infection With New-Onset Alcohol Use Disorder, January 2020 Through January 2022

**DOI:** 10.1001/jamanetworkopen.2022.55496

**Published:** 2023-02-09

**Authors:** Veronica R. Olaker, Ellen K. Kendall, Christina X. Wang, Theodore V. Parran, Pauline Terebuh, David C. Kaelber, Rong Xu, Pamela B. Davis

**Affiliations:** 1 Center for Artificial Intelligence in Drug Discovery, Case Western Reserve University School of Medicine, Cleveland, Ohio; 2 Center for Medical Education, Case Western Reserve University School of Medicine, Cleveland, Ohio; 3 Center for Community Health Integration, Case Western Reserve University School of Medicine, Cleveland, Ohio; 4 The Center for Clinical Informatics Research and Education, The MetroHealth System, Cleveland, Ohio

## Abstract

**Importance:**

The COVID-19 pandemic affects many diseases, including alcohol use disorders (AUDs). As the pandemic evolves, understanding the association of a new diagnosis of AUD with COVID-19 over time is required to mitigate negative consequences.

**Objective:**

To examine the association of COVID-19 infection with new diagnosis of AUD over time from January 2020 through January 2022.

**Design, Setting, and Participants:**

In this retrospective cohort study of electronic health records of US patients 12 years of age or older, new diagnoses of AUD were compared between patients with COVID-19 and patients with other respiratory infections who had never had COVID-19 by 3-month intervals from January 20, 2020, through January 27, 2022.

**Exposures:**

SARS-CoV-2 infection or non–SARS-CoV-2 respiratory infection.

**Main Outcomes and Measures:**

New diagnoses of AUD were compared in COVID-19 and propensity score–matched control cohorts by hazard ratios (HRs) and 95% CIs from either 14 days to 3 months or 3 to 6 months after the index event.

**Results:**

This study comprised 1 201 082 patients with COVID-19 (56.9% female patients; 65.7% White; mean [SD] age at index, 46.2 [18.9] years) and 1 620 100 patients with other respiratory infections who had never had COVID-19 (60.4% female patients; 71.1% White; mean [SD] age at index, 44.5 [20.6] years). There was a significantly increased risk of a new diagnosis of AUD in the 3 months after COVID-19 was contracted during the first 3 months of the pandemic (block 1) compared with control cohorts (HR, 2.53 [95% CI, 1.82-3.51]), but the risk decreased to nonsignificance in the next 3 time blocks (April 2020 to January 2021). The risk for AUD diagnosis increased after infection in January to April 2021 (HR, 1.30 [95% CI, 1.08-1.56]) and April to July 2021 (HR, 1.80 [95% CI, 1.47-2.21]). The result became nonsignificant again in blocks 7 and 8 (COVID-19 diagnosis between July 2021 and January 2022). A similar temporal pattern was seen for new diagnosis of AUD 3 to 6 months after infection with COVID-19 vs control index events.

**Conclusions and Relevance:**

Elevated risk for AUD after COVID-19 infection compared with non–COVID-19 respiratory infections during some time frames may suggest an association of SARS-CoV-2 infection with the pandemic-associated increase in AUD. However, the lack of excess hazard in most time blocks makes it likely that the circumstances surrounding the pandemic and the fear and anxiety they created also were important factors associated with new diagnoses of AUD.

## Key Points

**Question:**

Did the risk of a new alcohol use disorder (AUD) diagnosis after COVID-19 infection change over time during the pandemic?

**Findings:**

A cohort study using electronic health records of 2 821 182 US patients compared the risk of developing AUD after COVID-19 with that among patients after non–COVID-19 respiratory infections. An excess risk of a new diagnosis of AUD with COVID-19 was observed in the beginning of the pandemic, which then subsided, increased again for infections contracted from January to July 2021, and then became nonsignificant again after August 2021.

**Meaning:**

The results of this study suggest that the risk of a new diagnosis of AUD after a COVID-19 diagnosis may not be a consequence of the infection itself but rather associated with the context of the diagnosis and the pandemic.

## Teaser

This cohort study uses electronic health records of 2 821 182 US patients to examine associations of a new diagnosis of alcohol use disorder with COVID-19 over time from January 2020 through January 2022.

## Introduction

Alcohol use disorder (AUD) affects both the individual and society. The patient is at risk for disorders of the liver, pancreas, brain, gut, cardiovascular system, immune system, and musculoskeletal system as well as serious accidents affecting both the individual and those around them.^1^ In 2019, the Centers for Disease Control and Prevention (CDC) estimated the annual cost of AUD at $249 billion, including lost workplace productivity, medical care, accidents, and criminal justice system costs.^2^ For all these reasons, reports of increased AUD diagnoses during the COVID-19 pandemic are particularly concerning. Persons treated for other mental health disorders, those who consumed alcohol hazardously before the pandemic, and those with caregiving responsibilities^3^ were more likely to increase their alcohol use. Increased risk of AUD after COVID-19 diagnosis was reported in a retrospective cohort study of the US Department of Veterans Affairs population.^4^ Taquet et al^5^ found that the risk of psychiatric sequelae, including AUD, was significantly elevated for patients with a diagnosis of COVID-19 until December 13, 2020, as did a later study through October 20, 2021,^6^ but with a much smaller hazard ratio (HR). As the pandemic social context and SARS-CoV-2 variants evolve, it remains unclear how the risk of a new diagnosis of AUD after SARS-CoV-2 infection varies with time during the pandemic. Our study evaluated the risk of a new diagnosis of AUD by 3-month intervals and extends the evaluation into 2022, testing how risk has changed over time as the pandemic restrictions changed, vaccination and new therapeutics became available, viral variants evolved, and the societal views of the pandemic changed.^7^

## Methods

### Database Description

The TriNetX Analytics Platform was used to conduct this study, accessed from November 1 to December 14, 2022. The TriNetX COVID-19 Research USA No Date Shift Network was used. This network contains data from more than 60 million patients from 34 health care organizations in the United States. Data are deidentified per the Health Insurance Portability and Accountability Act (HIPAA) criteria—Section §164.514(a) of the HIPAA Privacy Rule. Studies using TriNetX data have been determined not to be human participant research and are exempt from review by the MetroHealth System institutional review board (Cleveland, Ohio). Patient consent was waived by the MetroHealth System institutional review board based on the deidentification of the data. In addition, TriNetX also has an exemption from the Western institutional review board based on deidentification of the data in a Health Insurance Portability and Accountability Act–compliant manner. TriNetX has been used extensively for epidemiologic analyses, including substance abuse and post–COVID-19 complications.^5,8,9^ This study followed the Strengthening the Reporting of Observational Studies in Epidemiology (STROBE) reporting guideline.

### Study Population and Cohort Definitions

**Figure 1.**
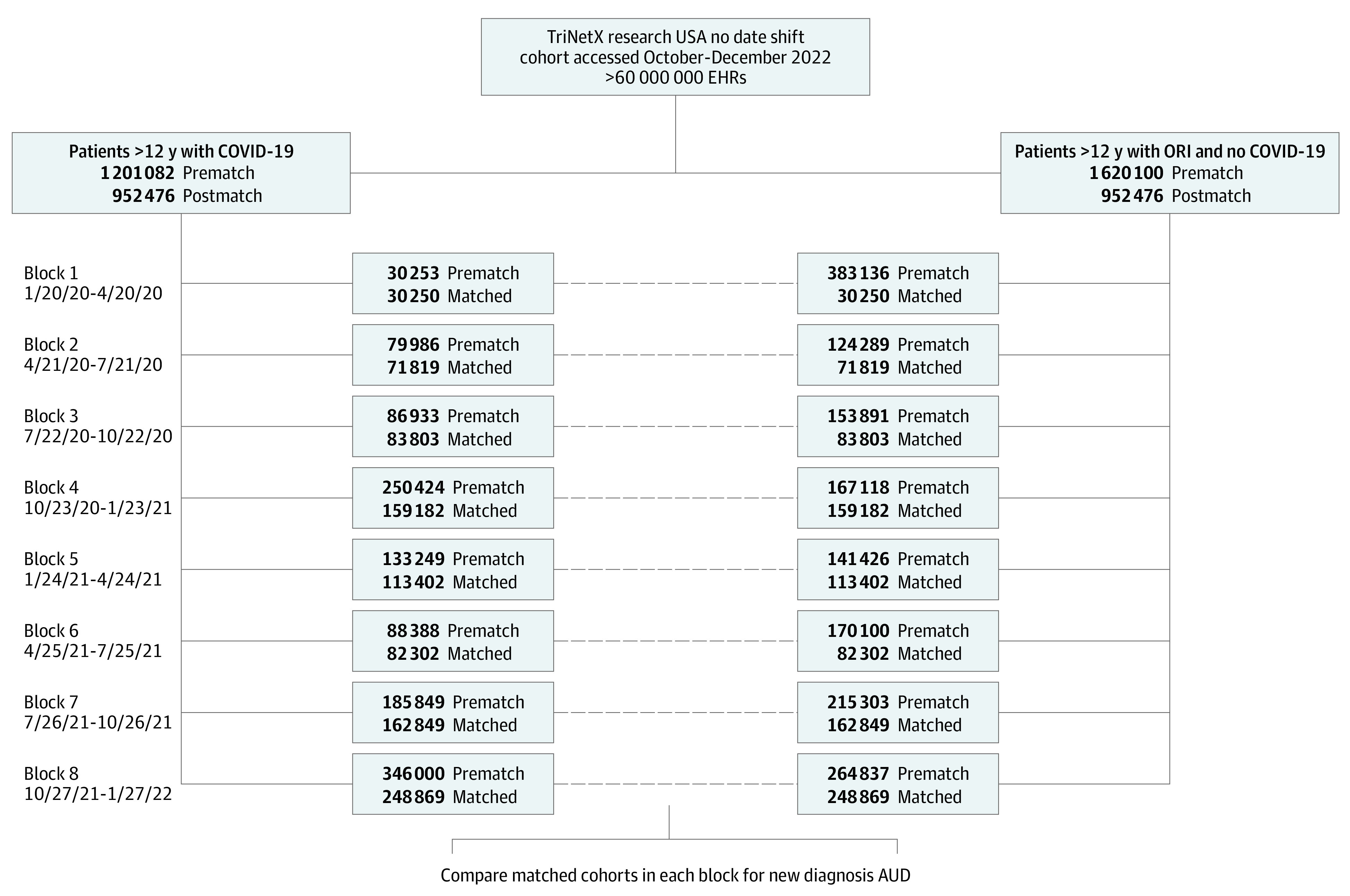
Flow Diagram for Building Cohorts in Defined Time Blocks The number of patients in both the COVID-19 and other respiratory infection (ORI) cohorts before and after propensity score matching is shown for each of the 8 time blocks that were analyzed. The dates that comprise each 3-month time block are shown in the first column. AUD indicates alcohol use disorder; EHR, electronic health record.

The total study population consisted of 2 821 182 patients 12 years of age or older: 1 201 082 with a diagnosis of COVID-19 (hereafter referred to as the COVID-19 cohort) and 1 620 100 who had other respiratory infections (ORIs) but had no prior COVID-19 documented in their electronic health records (EHRs) (based on a clinical encounter diagnosis code for COVID-19 or positive test result for SARS-CoV-2) (hereafter referred to as the ORI cohort). Patients with an encounter diagnosis of AUD before the follow-up window were excluded. Patients were excluded if they died before the follow-up analysis began, which was 14 days after the last day of the respective index event window. Each group was divided into 8 individual cohorts by 3-month blocks of time based on the time of initial infection from January 20, 2020, through January 27, 2022, to yield 16 cohorts: 8 with COVID-19 and 8 with ORIs (Figure 1). This temporal division provided cohorts of sufficient size for analysis in each block.

The index event for COVID-19 cohorts was an encounter diagnosis *International Statistical Classification of Diseases and Related Health Problems, Tenth Revision* (*ICD-10*) code for COVID-19 (U07.1). Additional codes were used for the first block of time from January 20 to April 20, 2020, to capture cases that were coded differently early in the pandemic (eMethods in Supplement 1). Separate analyses were run in which COVID-19 cohorts were defined by the presence of either a positive COVID-19 RNA test result or an *ICD-10* encounter diagnosis code of COVID-19 (U07.1) (eMethods in Supplement 1). The index event for ORI cohorts was defined by the presence of an *ICD-10* encounter diagnosis code of either acute upper respiratory tract infection (J00-J06), influenza and pneumonia (J09-J18), or other acute lower respiratory tract infection (J20-J22), as adapted from Taquet et al.^5^ To test the sensitivity of our findings to the index event, we also used bone fracture as a control index event (eMethods and eTables 3 and 4 in Supplement 1).

### Covariates and Outcomes

For analysis, the COVID-19 and ORI cohorts were matched for demographic characteristics, risk factors for greater severity of illness from COVID-19,^5^ other substance use disorders, mental health disorders that commonly co-occur with substance use disorders,^10^ family history of substance use, COVID-19 vaccination (starting in block 4), hospitalization, and *ICD-10* codes for socioeconomic status (Table). Race and ethnicity were obtained by self-report of patients and mapped by TriNetX to the US Government (Office of Management and Budget) designated categories for race and ethnicity. These data were used in propensity score matching since both COVID severity and prevalence of AUD are affected by race and ethnicity. Cohort matching used the TriNetX built-in propensity score matching function (1:1 matching using a nearest neighbor greedy matching algorithm with a caliper of 0.25 times the SD). The primary outcome was a new (or first-time) encounter diagnosis *ICD-10* code of AUD (F10: alcohol-related disorders).

**Table.** Baseline Patient Characteristics Before and After Matching for Block 1

Characteristic	Cohort before matching, No. (%)		SMD	Cohort after matching, No. (%)		SMD
	COVID-19 (n = 30 253)	ORI (n = 383 136)		COVID-19 (n = 30 250)	ORI (n = 30 250)	
Current age, mean (SD), y	52 (17.6)	46.8 (19.8)	0.27	51.9 (17.6)	52.5 (18.7)	0.032
Age at index, mean (SD), y	49.2 (17.6)	44.0 (19.9)	0.28	49.2 (17.6)	49.8 (18.7)	0.031
Sex						
Female	16 655 (55.1)	229 644 (59.9)	0.10	16 654 (55.1)	16 647 (55.0)	0.000
Male	13 429 (44.4)	143 244 (37.4)	0.14	13 427 (44.4)	13 434 (44.4)	0.000
Unknown	169 (0.6)	10 248 (2.7)	0.17	169 (0.6)	169 (0.6)	0.000
Ethnicity						
Hispanic or Latino	4430 (14.6)	33 360 (8.7)	0.19	4429 (14.6)	4723 (15.6)	0.027
Not Hispanic or Latino	17 164 (56.7)	269 091 (70.2)	0.28	17 162 (56.7)	17 258 (57.1)	0.006
Unknown	8659 (28.6)	80 685 (21.1)	0.18	8659 (28.6)	8269 (27.3)	0.029
Race						
Asian	1010 (3.3)	10 184 (2.7)	0.04	1009 (3.3)	970 (3.2)	0.007
Black or African American	7828 (25.9)	59 195 (15.5)	0.26	7827 (25.9)	7500 (24.8)	0.025
Native American or Alaska Native	100 (0.3)	1188 (0.3)	0.00	100 (0.3)	100 (0.3)	0.000
Native Hawaiian or Other Pacific Islander	68 (0.2)	564 (0.1)	0.02	68 (0.2)	53 (0.2)	0.011
Unknown	6408 (21.2)	45 472 (11.9)	0.25	6407 (21.2)	6876 (22.7)	0.037
White	14 839 (49.1)	266 533 (69.6)	0.43	14 839 (49.1)	14 751 (48.8)	0.006
Generalized anxiety disorder	1590 (5.3)	25 250 (6.6)	0.06	1590 (5.3)	1478 (4.9)	0.017
Phobic anxiety disorders	540 (1.8)	6434 (1.7)	0.01	540 (1.8)	505 (1.7)	0.009
Other anxiety disorders	5495 (18.2)	84 732 (22.1)	0.10	5493 (18.2)	5200 (17.2)	0.025
Reaction to severe stress, and adjustment disorders	1691 (5.6)	22 340 (5.8)	0.01	1691 (5.6)	1567 (5.2)	0.018
Depressive episode	5087 (16.8)	63 772 (16.6)	0.00	5085 (16.8)	4676 (15.5)	0.037
Major depressive disorder, recurrent	1335 (4.4)	16 911 (4.4)	0.00	1334 (4.4)	1219 (4.0)	0.019
Bipolar disorder	686 (2.3)	8045 (2.1)	0.01	686 (2.3)	657 (2.2)	0.007
Personal history of other mental and behavioral disorders	628 (2.1)	4455 (1.2)	0.07	626 (2.1)	586 (1.9)	0.009
Attention-deficit/hyperactivity disorders	715 (2.4)	12 972 (3.4)	0.06	715 (2.4)	661 (2.2)	0.012
Borderline personality disorder	116 (0.4)	1031 (0.3)	0.02	116 (0.4)	99 (0.3)	0.009
Schizophrenia, schizotypal, delusional, and other nonmood psychotic disorders	869 (2.9)	5289 (1.4)	0.10	868 (2.9)	810 (2.7)	0.012
Antisocial personality disorder	21 (0.1)	90 (0.02)	0.02	21 (0.1)	22 (0.1)	0.001
Family history of other psychoactive substance abuse and dependence	176 (0.6)	584 (0.2)	0.07	175 (0.6)	146 (0.5)	0.013
Persons with potential health hazards related to socioeconomic and psychosocial circumstances	2053 (6.8)	16 031 (4.2)	0.11	2051 (6.8)	1801 (6.0)	0.034
Opioid-related disorders	446 (1.5)	4857 (1.3)	0.02	446 (1.5)	380 (1.3)	0.019
Cannabis-related disorders	526 (1.7)	5430 (1.4)	0.03	526 (1.7)	482 (1.6)	0.011
Sedative-, hypnotic-, or anxiolytic-related disorders	94 (0.3)	922 (0.2)	0.01	94 (0.3)	63 (0.2)	0.020
Cocaine-related disorders	175 (0.6)	1779 (0.5)	0.02	175 (0.6)	151 (0.5)	0.011
Other stimulant-related disorders	471 (1.6)	3079 (0.8)	0.07	470 (1.6)	436 (1.4)	0.009
Hallucinogen-related disorders	119 (0.4)	557 (0.1)	0.05	117 (0.4)	83 (0.3)	0.020
Nicotine dependence	3416 (11.3)	45 028 (11.8)	0.01	3415 (11.3)	3172 (10.5)	0.026
Inhalant-related disorders	96 (0.3)	1371 (0.4)	0.01	96 (0.3)	103 (0.3)	0.004
Other psychoactive substance–related disorders	1349 (4.5)	8344 (2.2)	0.13	1346 (4.5)	1199 (4.0)	0.024

Abbreviations: ORI, other respiratory infections; SMD, standard mean difference.

### Statistical Analysis

We compared the risk of the outcome between the COVID-19 cohorts and the ORI cohorts in 8 index time frames spanning the COVID-19 pandemic using HRs and 95% CIs. We studied 2 outcome follow-up periods: 14 days to 3 months after the index event and 3 to 6 months after the index event. All statistical analyses were conducted in the TriNetX Analytics Platform. Kaplan-Meier analysis was used to estimate the probability of clinical outcomes. Cox proportional hazards regression analysis was used to compare the matched cohorts. The proportional hazard assumption was tested using the generalized Schoenfeld approach. The TriNetX Platform calculates HRs and associated 95% CIs, using R’s Survival package, version 3.2-3 (R Group for Statistical Computing). Hypothesis tests were 2-sided, and results were deemed statistically significant at *P* < .05.

## Results

This study comprised 1 201 082 patients with COVID-19 (56.9% female; 65.7% White; mean [SD] age at index, 46.2 [18.9] years) and 1 620 100 patients with ORIs (60.4% female; 71.1% White; mean [SD] age at index, 44.5 [20.6] years). Figure 1 shows the distribution of the population among the 8 time blocks, before and after matching. After matching, the total patient population was 1 904 952, with 952 476 in the COVID-19 cohorts and the same number in the ORI cohort. The standard mean difference was less than 0.1 for all covariates after matching. The risk of a new diagnosis of AUD was compared in the COVID-19 cohorts and matched ORI cohorts in the same time frame. The numbers of COVID-19 cases in each block increased over blocks 1 to 4, decreased in blocks 5 and 6, and increased markedly in blocks 7 and 8, consistent with CDC reports of COVID-19 cases in the US during this time. Patient characteristics before and after matching for demographic characteristics, AUD comorbidities, and risk factors for substance use disorders are shown for block 1 in the Table and for the other time blocks in eTables 1 to 8 in Supplement 1.

**Figure 2.**
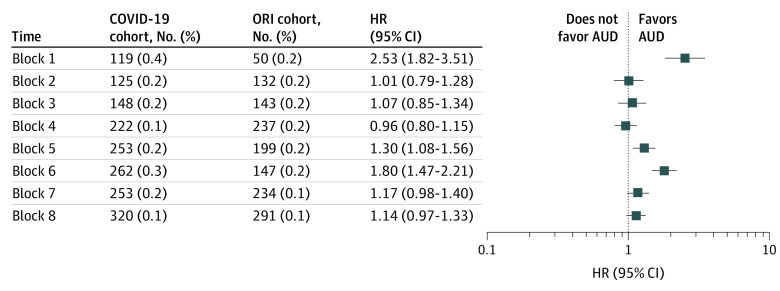
Risk of New Alcohol Use Disorder (AUD) Diagnosis After COVID-19 Diagnosis vs Other Respiratory Infection (ORI) Diagnosis From 14 Days to 3 Months After Index Event Hazard ratios (HRs) for the COVID-19 vs ORI cohorts across the 8 time blocks for the outcome of a first *International Statistical Classification of Diseases and Related Health Problems, Tenth Revision* (*ICD-10*) encounter diagnosis of an alcohol-related disorder (coded as F10). These HRs are for the follow-up window of an *ICD-10* encounter diagnosis from 14 days to 3 months after the index event of either COVID-19 or a non–COVID-19 ORI.

**Figure 3.**
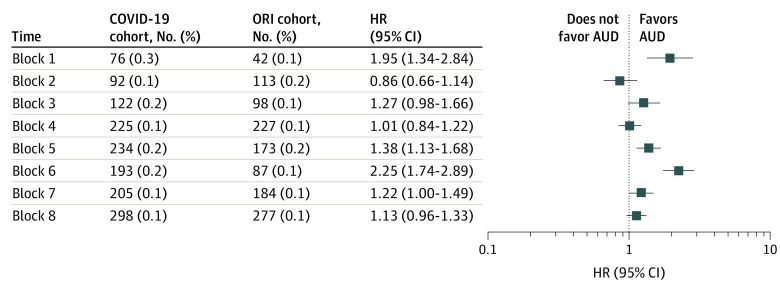
Risk of New Alcohol Use Disorder (AUD) Diagnosis After COVID-19 Diagnosis vs Other Respiratory Infection (ORI) Diagnosis From 3 to 6 Months After Index Event Hazard ratios (HRs) for the COVID-19 vs ORI cohorts across the 8 time blocks for the outcome of a first *International Statistical Classification of Diseases and Related Health Problems, Tenth Revision* (*ICD-10*) encounter diagnosis of an alcohol-related disorder (coded as F10). These HRs are for the follow-up window of an *ICD-10* encounter diagnosis from 3 to 6 months after the index event of either COVID-19 or a non–COVID-19 ORI.

For block 1 (January to March 2020), which began when the first case of COVID-19 was diagnosed in the US, there was a significantly increased HR for a new *ICD-10* encounter diagnosis of AUD for patients in the 14 days to 3 months after COVID-19 diagnosis: 119 of 30 250 patients (0.4%) in the COVID-19 cohort and 50 of 30 250 patients (0.2%) in the ORI cohort (HR, 2.53 [95% CI, 1.82-3.51]) (Figure 2). From 3 to 6 months after the index event, there was also a significant increase in new *ICD-10* encounter diagnoses of AUD: 76 of 30 250 patients (0.3%) in the COVID-19 cohort and 42 of 30 250 patients (0.1%) in the ORI cohort (HR, 1.95 [95% CI, 1.34-2.84]) (Figure 3).

For the follow-up period of 14 days to 3 months, there was no increased HR of a new *ICD-10* AUD encounter diagnosis after COVID-19 in blocks 2 to 4 (block 2: HR, 1.01 [95% CI, 0.79-1.28]; block 3: HR, 1.07 [95% CI, 0.85-1.34]; block 4: HR, 0.96 [95% CI, 0.80-1.15]), but the HR increased in blocks 5 and 6 (block 5: HR, 1.30 [95% CI, 1.08-1.56]; block 6: HR, 1.80 [95% CI, 1.47-2.21]), then became nonsignificant in blocks 7 and 8 (block 7: HR, 1.17 [95% CI, 0.98-1.40]; block 8: HR, 1.14 [95% CI, 0.97-1.33]) (Figure 2). The number of patients with *ICD-10* encounter diagnoses with AUD increased steadily over the period of the study.

New encounter diagnoses of AUD followed a similar pattern 3 to 6 months after the index events (Figure 3). For block 1, there was a significantly increased HR of a new AUD encounter diagnosis in the COVID-19 cohort compared with the ORI cohort (HR, 1.95 [95% CI, 1.34-2.84]). The HR became nonsignificant for blocks 2 to 4 (block 2: HR, 0.86 [95% CI, 0.66-1.14]; block 3: HR, 1.27 [95% CI, 0.98-1.66]; block 4: HR, 1.01 [95% CI, 0.84-1.22]), then increased significantly in blocks 5 and 6 (block 5: HR, 1.38 [95% CI, 1.13-1.68]; block 6: HR, 2.25 [95% CI, 1.74-2.89]), and became nonsignificant in blocks 7 and 8 (block 7: HR, 1.22 [95% CI, 1.00-1.49]; block 8: HR, 1.13 [95% CI, 0.96-1.33]). The number of patients with an encounter diagnosis for AUD after COVID-19 generally increased across the 8 time blocks.

Similar temporal patterns were observed if the definition of COVID-19 diagnosis expanded to include patients with a positive polymerase chain reaction test result (eFigure 1 and 2 in Supplement 1). Similar temporal patterns were also observed when the index event in the control group was an encounter diagnosis for a fracture (eFigure 3 and 4 in Supplement 1). There was a modestly increased HR for COVID-19 vs ORI during the period of Delta variant predominance, with an HR of 1.18 (95% CI, 1.02-1.38) for new AUD encounter diagnoses 14 days to 3 months after infection and an HR of 1.30 (95% CI, 1.11-1.53) for new diagnoses 3 to 6 months after infection compared with ORI (eFigure 5 and 6 in Supplement 1). We considered a variant to be predominant when CDC sequencing detected it in more than 90% of sequenced samples (for Delta, July 15 to November 30, 2021, and for Omicron, after December 15, 2021).^11^

## Discussion

Our results demonstrate that the excess risk of a new AUD encounter diagnosis after a SARS-CoV-2 infection was significantly increased for those who contracted COVID-19 compared with patients with an ORI in the first 3 months of the pandemic. The HRs decreased in blocks 2 to 4 but increased in blocks 5 and 6 (January 25 to July 26, 2021). There was no difference between the COVID-19 and ORI cohorts for people infected starting July 26, 2021, and after.

Several possibilities could account for the variable excess risk of an AUD encounter diagnosis after a COVID-19 infection as the pandemic continued. Higher HRs for an AUD encounter diagnosis among individuals who contracted COVID-19 compared with those who had an encounter diagnosis for ORI or fracture might suggest some biological association of the virus, which is supported by the modest increase in HR after infection in the Delta variant–predominant phase, if the characteristics of the virus itself are associated with an AUD diagnosis. However, the lack of increased HR compared with control cohorts in blocks 2 to 4 and blocks 7 to 8, together with the decrease in absolute risk for a new AUD encounter diagnosis after COVID-19 in those same time blocks (Figure 2 and Figure 3), argue against a biological effect of SARS-CoV-2 accounting entirely for the increased HR. New encounter diagnoses of AUD increased after COVID-19 infections contracted in blocks 5 and 6 (January 25 to July 26, 2021). During the follow-up period, when the new diagnoses of AUD after these infections encounters occurred (April 26, 2021, to January 25, 2022), the US experienced a surge of COVID-19 cases that followed a months-long decrease, with the emergence of the Delta variant followed by the highly contagious Omicron variant.

Another possible explanation for the decrease in HR over time seen in the population infected with SARS-CoV-2 is the increase in undiagnosed or unreported SARS-CoV-2 infections among individuals in the control cohorts. The EHRs might not contain information on non–laboratory-confirmed asymptomatic infections or infections diagnosed at home, both of which increased in the Omicron variant–predominant period.^12^ By January 2022 (the last date of our analysis), an estimated 50% of adults had evidence of natural infection, based on antibodies to SARS-CoV-2 nucleocapsid, in testing of discarded blood samples obtained for other, unknown purposes. These samples probably included individuals who were already known to have COVID-19 and may not represent the general population. Our data show that the proportion of the control population who subsequently had an AUD diagnosis did not increase over time during the pandemic. If increasing unrecognized infections occurred in the control group, and infection was associated with AUD encounter diagnosis, we would have expected that proportion to increase. Although unrecognized infections among control participants may be associated with the observations, they are unlikely to account for them entirely.

It seems likely that the social context of the pandemic, including anxiety, fear, social isolation, stress, and other contextual factors, is associated with this time-related trend. An encounter diagnosis of COVID-19, particularly at the outset of the pandemic, was alarming because of the high rate of hospitalization and death from COVID-19, the persistence of symptoms long after acute disease subsided, and the suggestion that unknown long-term consequences of COVID-19 infection might ensue. After Hurricane Katrina, stress, anxiety, and resource deprivation were considered to be associated with a surge in AUD (as well as smoking and posttraumatic stress disorder).^13^ As the COVID-19 pandemic continued, several factors may have alleviated anxiety and stress. An effective vaccine offered a means for protection. One study reported that individuals who received their first dose of a COVID-19 vaccine from December 2020 to March 2021 reported lower levels of mental distress compared with individuals who did not receive the vaccine.^14^ Also, the emergence of different variants may affect the anxiety surrounding COVID-19. When the Delta variant produced a surge in cases after several months’ decrease in cases, there was great consternation and fear. This period coincided with a secondary surge of new AUD encounter diagnoses in our study. With the shift to the highly contagious but less-severe Omicron variant, which also has lower risk for long COVID,^15^ the surge in AUD encounter diagnoses abated. In addition, the development of effective treatments, both immunologic and antiviral, may mitigate fear of infection. It seems likely that some biological factor of COVID-19 plus a strong element of the circumstances of the pandemic was associated with our observed changes in excess hazard for new encounter diagnoses of AUD. In addition, the biological effect of variants that emerged later may differ from earlier variants, and this, combined with the effect of vaccines and other interventions, may be associated with changes in excess AUD encounter diagnoses over time during the pandemic.

Besides the effect of changes in severity and available prophylaxis and treatment of COVID-19, the pandemic responses changed over time. Recommendations for isolation time after diagnosis of COVID-19 have decreased from 10 days^16^ to only 5 days.^17^ Weerakoon et al^18^ found that, for every 1 week spent at home during the pandemic, the odds of binge drinking increased by 1.19 times, and by 1.77 times for those with diagnosed depression. This finding aligns with a systematic review of the literature on drinking behavior during the pandemic, which found that preexisting mental conditions, especially anxiety and depression, were associated with a higher risk of increased drinking behavior.^3^ Other changes that might mitigate stress include the reopening of in-person schooling for children in kindergarten through grade 12, freeing parents from caregiving and instructional responsibilities; reopening of businesses; economic recovery; relaxation of travel restrictions; and improved availability of goods and services. On balance, we suggest that the pandemic context is associated with the secular trend in excess risk for AUD encounter diagnoses.

We considered the possibility that bias of ascertainment might account for the increased risk for an AUD encounter diagnosis among patients who contracted SARS-CoV-2, because these patients may have had increased frequency of contact with the medical system, providing more opportunities for an AUD to be detected. However, comparison of patients with COVID-19 with another control cohort with an encounter diagnosis of a large bone fracture,^5^ a diagnosis also likely to involve follow-up, also showed increased risk for an AUD encounter diagnosis after COVID-19 at the beginning of the pandemic, and the association followed a similar pattern to the comparison with ORI, suggesting that simple bias of ascertainment may not entirely account for our observations.

The presence of a significant risk of an AUD encounter diagnosis after COVID-19 in the 3- to 6-month follow-up period is interesting. Those who had COVID-19 from January 20 to April 20, 2020, had a much higher risk of an AUD encounter diagnosis from 3 to 6 months after infection compared with those who had a non–COVID-19 ORI encounter diagnosis. One possibility is that AUD may be exacerbated if COVID-19 symptoms fail to resolve. In addition, AUD tends to not be diagnosed until it has reached the later stages, or the moderate and severe categories, and the natural history of alcohol abuse develops over approximately 3 to 15 years.^19^ Perhaps early COVID-19 infection involved stress and isolation that triggered clinically recognizable AUD that was previously undetected among patients who were considered low-risk drinkers. Alternatively, stress applied when COVID-19 was diagnosed may have continued over a period of months due to prolonged symptoms and continuing social isolation, resulting in persistent inappropriate alcohol consumption and an encounter diagnosis of AUD. It is also possible that a diagnosis of COVID-19 simply brought people with AUD to medical attention.

Our results confirm those of Taquet et al^5^ for the early period of the pandemic and may explain the lower HR reported in a second study,^6^ which included a longer pandemic time period, during which, our data show, the HR was decreasing, diluting the strong early association. Our results for the overall prevalence of AUD encounter diagnoses are consistent with values reported in the literature (2.5%-4%).^20^ We provide new support for the association of the pandemic context with the emergence of AUD.

### Limitations

This study has some limitations, including its observational retrospective nature, which permits us to identify association but not causation. The results here should therefore be viewed as hypothesis generating. In addition, the EHR is subject to overdiagnosis, underdiagnosis, or misdiagnosis. Electronic health records may not capture all the relevant data about patients, especially treatments, vaccinations, or diagnostic tests obtained outside of health care systems or the details of the social determinants of health. With the cumulative incidence of nonmedically attended illnesses and wider availability of home testing over time, COVID-19 misclassification may have increased over time.

The population captured in TriNetX, although quite large, may not be representative of the US population, so these studies should be repeated in other cohorts. In this study, AUD was defined based on *ICD-10* code F10 (alcohol-related disorders). Due to sample size limitations, we did not further examine the associations of COVID-19 with AUD subgroups, including alcohol abuse and alcohol dependence.

## Conclusions

The results of this cohort study suggest that patients who had COVID-19 in the first 3 months of the COVID-19 pandemic had a higher risk of having an encounter diagnosis of AUD 14 days to 6 months after infection than those who had never been identified as having COVID-19 but had an ORI encounter diagnosis or fracture encounter diagnosis during the same time period. The risk of AUD decreased for the next 9 months, then increased for 6 months, and decreased again until the end of the study period. The reasons for this temporal variation remain to be determined definitively, but contextual factors of SARS-CoV-2 infection and the pandemic itself are likely important factors. Future work is required to clarify the relative associations of COVID-19 itself and the pandemic context in increasing risk for addictive disease.

## Notes

Supplement 1.
eMethods.
**eFigure 1.** Risk of New AUD Diagnosis After COVID-19 Including Positive Lab Test in Cohort Definition vs ORI Diagnosis From 14 Days to 3 Months After Index Event **eFigure 2.** Risk of New AUD Diagnosis After COVID-19 Including Positive Lab Test in Cohort Definition vs ORI Diagnosis From 3 to 6 Months After Index Event **eFigure 3.** Risk of New AUD Diagnosis After COVID-19 Diagnosis vs Bone Fracture Diagnosis From 14 Days to 3 Months After Index Event **eFigure 4.** Risk of New AUD Diagnosis After COVID-19 Diagnosis vs Bone Fracture Diagnosis From 3 to 6 Months After Index Event **eFigure 5.** Risk of New AUD Diagnosis After COVID-19 Diagnosis vs ORI Diagnosis in Delta and Omicron Periods From 14 Days to 3 Months After Index Event **eFigure 6.** Risk of New AUD Diagnosis After COVID-19 Diagnosis vs ORI Diagnosis in Delta and Omicron Periods From 3 to 6 Months After Index Event **eTable 1. **Baseline Characteristics for Block 1 Before and After Matching **eTable 2. **Baseline Characteristics for Block 2 Before and After Matching **eTable 3. **Baseline Characteristics for Block 3 Before and After Matching **eTable 4. **Baseline Characteristics for Block 4 Before and After Matching **eTable 5. **Baseline Characteristics for Block 5 Before and After Matching **eTable 6. **Baseline Characteristics for Block 6 Before and After Matching **eTable 7. **Baseline Characteristics for Block 7 Before and After Matching **eTable 8. **Baseline Characteristics for Block 8 Before and After Matching+A1:G117

Supplement 2.
Data Sharing Statement
