# Supplementary material for: Association of Recent SARS-CoV-2 Infection With New-Onset Alcohol Use Disorder, January 2020 Through January 2022
Source: JAMA Netw Open. 2023 Feb 9;6(2):e2255496. doi: 10.1001/jamanetworkopen.2022.55496 (PMC9912133; doi:10.1001/jamanetworkopen.2022.55496)
Supplement: Supplement 1. — eMethods. eFigure 1. Risk of New AUD Diagnosis After COVID-19 Including Positive Lab Test in Cohort Definition vs ORI Diagnosis From 14 Days to 3 Months After Index Event eFigure 2. Risk of New AUD Diagnosis After COVID-19 Including Positive Lab Test in Cohort Definition vs ORI Diagnosis From 3 to 6 Months After Index Event eFigure 3. Risk of New AUD Diagnosis After COVID-19 Diagnosis vs Bone Fracture Diagnosis From 14 Days to 3 Months After Index Event eFigure 4. Risk of New AUD Diagnosis After COVID-19 Diagnosis vs Bone Fracture Diagnosis From 3 to 6 Months After Index Event eFigure 5. Risk of New AUD Diagnosis After COVID-19 Diagnosis vs ORI Diagnosis in Delta and Omicron Periods From 14 Days to 3 Months After Index Event eFigure 6. Risk of New AUD Diagnosis After COVID-19 Diagnosis vs ORI Diagnosis in Delta and Omicron Periods From 3 to 6 Months After Index Event eTable 1. Baseline Characteristics for Block 1 Before and After Matching eTable 2. Baseline Characteristics for Block 2 Before and After Matching eTable 3. Baseline Characteristics for Block 3 Before and After Matching eTable 4. Baseline Characteristics for Block 4 Before and After Matching eTable 5. Baseline Characteristics for Block 5 Before and After Matching eTable 6. Baseline Characteristics for Block 6 Before and After Matching eTable 7. Baseline Characteristics for Block 7 Before and After Matching eTable 8. Baseline Characteristics for Block 8 Before and After Matching+A1:G117 [file jamanetwopen-e2255496-s001.pdf]

Supplementary Online Content

Olaker VR, Kendall EK, Wang CX, et al. Association of recent SARS-CoV-2 infection with new-onset alcohol use disorder, January 2020 through January 2022. *JAMA Netw Open.* 2023;6(2):e2255496. doi:10.1001/jamanetworkopen.2022.55496

eMethods.
eFigure 1. Risk of New AUD Diagnosis After COVID-19 Including Positive Lab Test in Cohort Definition vs ORI Diagnosis From 14 Days to 3 Months After Index Event
eFigure 2. Risk of New AUD Diagnosis After COVID-19 Including Positive Lab Test in Cohort Definition vs ORI Diagnosis From 3 to 6 Months After Index Event
eFigure 3. Risk of New AUD Diagnosis After COVID-19 Diagnosis vs Bone Fracture Diagnosis From 14 Days to 3 Months After Index Event
eFigure 4. Risk of New AUD Diagnosis After COVID-19 Diagnosis vs Bone Fracture Diagnosis From 3 to 6 Months After Index Event
eFigure 5. Risk of New AUD Diagnosis After COVID-19 Diagnosis vs ORI Diagnosis in Delta and Omicron Periods From 14 Days to 3 Months After Index Event
eFigure 6. Risk of New AUD Diagnosis After COVID-19 Diagnosis vs ORI Diagnosis in Delta and Omicron Periods From 3 to 6 Months After Index Event
eTable 1. Baseline Characteristics for Block 1 Before and After Matching
eTable 2. Baseline Characteristics for Block 2 Before and After Matching
eTable 3. Baseline Characteristics for Block 3 Before and After Matching
eTable 4. Baseline Characteristics for Block 4 Before and After Matching
eTable 5. Baseline Characteristics for Block 5 Before and After Matching
eTable 6. Baseline Characteristics for Block 6 Before and After Matching
eTable 7. Baseline Characteristics for Block 7 Before and After Matching
eTable 8. Baseline Characteristics for Block 8 Before and After Matching+A1:G117

This supplementary material has been provided by the authors to give readers additional information about their work.

© 2023 Olaker VR et al. *JAMA Network Open.*

eMethods.

TriNetX description

TriNetX Database and Statistical Analysis Description of TriNetX database: The data used in this study was accessed between November 1 and December 15, 2022 from the TriNetX Research USA No Date Shift Network. This resource provides access to electronic medical records (diagnoses, procedures, medications, laboratory values, genomic information) from over 92 million patients from 34 healthcare organizations, which is de-identified per criteria from the Health Insurance Portability and Accountability Act (HIPAA), Section §164.514(a) of the HIPAA Privacy Rule. MetroHealth System, Cleveland, Ohio, IRB has determined any research using TriNetX, is not Human Subject Research and therefore exempt from IRB review.

The TriNetX Research USA No Date Shift Network platform de-identifies and aggregates electronic health record (EHR) data from 34 contributing healthcare systems, most of which are large academic medical institutions with both inpatient and outpatient facilities at multiple locations across the US. Patient EHR data includes information from hospitals, primary care, and specialty treatment providers, covering diverse geographic locations, age groups, racial and ethnic groups, income levels and insurance types including various commercial insurances, governmental insurance (Medicare and Medicaid), self-pay/uninsured, worker compensation insurance, military/VA insurance among others. Race and ethnicity data in TriNetX is derived from self-reports in the clinical EHR systems, which is then mapped to the following categories: (1) Race: Asian, American Indian or Alaskan Native, Black or African American, Native Hawaiian or Other Pacific Islander, White, Unknown race; and (2) Ethnicity: Hispanic or Latino, Not Hispanic or Latino, Unknown Ethnicity.

Cohort definitions

The primary COVID-19 cohorts were defined by the presence of the diagnostic code for COVID-19, U07.1. Since this code was not available until April 1st, 2020 (citation), an alternate inclusion criteria list was used for block one only (indexed 1/20/2020-4/20/2020). The terms used to capture these earlier cases of COVID-19 is below:

U07.1: COVI-19
B34.2: Coronavirus infection, unspecified
B97.29: Other coronavirus as the cause of diseases classified elsewhere
J12.81: Pneumonia due to SARS-associated coronavirus
U07.2: COVID-19, virus not identified (WHO)

One term was also excluded for this early-COVID-19 group, 079.89: other specified viral infection. This method of querying for COVID-19 patients before U07.1 was available was recommended by TriNetX. COVID-19 patients were excluded from the cohorts if they had a COVID-19 diagnosis 3 months prior to that specific index window.

The other respiratory infection control cohorts were defined by the presence of any of the following diagnoses:

J00: Acute nasopharyngitis [common cold]
J01: Acute sinusitis
J02: Acute pharyngitis
J03: Acute tonsillitis
J04: Acute laryngitis and tracheitis
J05: Acute obstructive laryngitis [croup] and epiglottitis
J06: Acute upper respiratory infections of multiple and unspecified sites

J09: Influenza due to certain identified influenza virus
J10: Influenza due to other identified influenza virus
J11: Influenza due to unidentified influenza virus
J12: Viral pneumonia, not elsewhere classified
J13: Pneumonia due to Streptococcus pneumoniae
J14: Pneumonia due to Hemophilus influenzae
J15: Bacterial pneumonia, not elsewhere classified
J16: Pneumonia due to other infectious organisms, not elsewhere classified
J17: Pneumonia in diseases classified elsewhere
J18: Pneumonia, unspecified organism
J20: Acute bronchitis
J21: Acute bronchiolitis
J22: Unspecified acute lower respiratory infection
This cohort definition was defined by Taquet et al[1]

The exclusion criteria for the ORI control cohorts excludes the codes listed below. There are two separate groups, both of which were excluded for the control cohorts. The first group aims to exclude patients who had COVID-19 early in the pandemic before the code U07.1 became available on April 1st, 2020. The codes contained in the second list were excluded if they occurred in a patient's chart at any point in time. Positive COVID-19 antibody tests were not excluded because we did not want to exclude patients who were vaccinated but were never infected with COVID-19.

First terms for COVID-19: excluded if these occurred between January 20th, 2020 and March 31st, 2020 for control cohorts:
B97.29: Other coronavirus as the cause of diseases classified elsewhere
B34.2: Coronavirus infection, unspecified
J12.81: Pneumonia due to SARS-associated coronavirus

Codes excluded for occurring at any point in time for control cohorts:
J12.82: Pneumonia due to COVID-19
U07.1: COVID-19
U07.2: COVID-19, virus not identified (WHO)
94311-8: SARS coronavirus 2 N gene [Cycle Threshold #] in Unspecified specimen by Nucleic acid amplification using CDC primer-probe set N1 (at most 40.00 units)
9088: SARS coronavirus 2 and related RNA [Presence] (labResult: Positive)
94312-6: SARS coronavirus 2 N gene [Cycle Threshold #] in Unspecified specimen by Nucleic acid amplification using CDC primer-probe set N2 (at most 40.00 units)
94558-4: SARS coronavirus 2 Ag [Presence] in Respiratory specimen by Rapid immunoassay (labResult: Positive)
95522-9: SARS-CoV-2 (COVID-19) N gene [Log #/volume] (viral load) in Respiratory specimen by NAA with probe detection (at least 0.80 {Log_copies}/mL)
94511-3: SARS-CoV-2 (COVID19) ORF1ab region [Cycle Threshold #] in Unspecified specimen by NAA with probe detection (at most 40.00 units)
94643-4: SARS-CoV-2 (COVID-19) S gene [Cycle Threshold #] in Specimen by NAA with probe detection (at most 40.00 {Ct_value})
94745-7: SARS coronavirus 2 RNA [Cycle Threshold #] in Respiratory specimen by NAA with probe detection (at most 40.00 units)
97097-0: SARS-CoV-2 (COVID-19) Ag [Presence] in Upper respiratory specimen by Rapid immunoassay (labResult: Positive)
96764-6: SARS-CoV-2 (COVID-19) E gene [Cycle Threshold #] in Respiratory specimen by NAA with probe detection (at most 40.00 units (most recent occurrence))

© 2023 Olaker VR et al. *JAMA Network Open.*

95209-3: SARS coronavirus+SARS coronavirus 2 Ag [Presence] in Respiratory specimen by Rapid immunoassay (labResult: Positive)
94763-0: SARS-CoV-2 (COVID-19) [Presence] in Unspecified specimen by Organism specific culture (labResult: Positive)
96603-6: SARS-CoV-2 (COVID-19) S protein RBD neutralizing antibody [Presence] in Serum or Plasma by Immunoassay (labResult: Positive)
96119-3: SARS-CoV-2 (COVID-19) Ag [Presence] in Upper respiratory specimen by Immunoassay (labResult: Positive)
For both COVID-19 and control cohorts, patients were excluded if they had ever been diagnosed with an F10 code (alcohol related disorders) prior to their index event.
Lastly, patients in all cohorts (both COVID-19 and controls) were excluded if they had died up until 2 weeks after the last day of the index event window. This ensured that only alive patients were included in the analyses up until the follow-up window for outcomes began, which was two weeks after the index event for patients, even if a patient had the index event on the very last day of their respective index event time window.

Full covariate list

82 covariates were matched for in analyses for blocks 1-3, while 100 were matched for in analyses for blocks 4-8. The extra 18 covariates are codes for COVID-19 vaccination. The first group of covariates matched across all analyses was demographics, including: current age, age at index, female, male, unknown gender, American Indian or Alaska Native, Asian, Black or African American, Native Hawaiian or other Pacific Islander, unknown race, White, Hispanic or Latinx, not Hispanic or Latinx, and unknown ethnicity. The next group of covariates matched across all analyses are risk factors for more severe COVID-19, and this list was the same used by Taquet et al.
E66: Overweight and obesity
I10-I16: Hypertensive diseases
E10: Type 1 diabetes mellitus
E11: Type 2 diabetes mellitus
J40: Bronchitis, not specified as acute or chronic
J41: Simple and mucopurulent chronic bronchitis
J42: Unspecified chronic bronchitis
J43: Emphysema
J44: Other chronic obstructive pulmonary disease
J45: Asthma
J47: Bronchiectasis
I30-I15A: Other forms of heart disease
I12: Hypertensive chronic kidney disease
K72: Hepatic failure, not elsewhere classified
K73: Chronic hepatitis, not elsewhere classified
K74: Fibrosis and cirrhosis of liver
K76.1: Chronic passive congestion of liver
K76.0: Fatty (change of) liver, not elsewhere classified
K76.6: Portal hypertension
K76.8: Other specified diseases of liver
I63: Cerebral infarction
F01: Vascular dementia
F02: Dementia in other diseases classified elsewhere
F03: Unspecified dementia
G30: Alzheimer's disease
G31.0: Frontotemporal dementia

© 2023 Olaker VR et al. *JAMA Network Open.*

G31.83: Dementia with Lewy bodies
C00-D49: Neoplasms
C81-C96: Malignant neoplasms of lymphoid, hematopoietic and related tissue
M05: Rheumatoid arthritis with rheumatoid factor
M06: Other rheumatoid arthritis
M32: Systemic lupus erythematosus (SLE)
L40: Psoriasis
D80-D89: Certain disorders involving the immune mechanism
1008098: Renal Transplantation Procedures
1007811: Liver Transplantation Procedures
BMI, categorized into 3 groups: <25kg/m^2, 25-30kg/m^2, >30kg/m^2
Blood pressure, systolic, categorized into 3 groups: <140 mm[Hg], 140-160 mm[Hg], >160 mm[Hg]
Blood pressure, diastolic, categorized into 3 groups: <90 mm[Hg], 90-100 mm[Hg], >100 mmHg

Codes for prior hospitalization were also matched between cohorts:
1013659: Hospital Inpatient Services
1013661: New or Established Patient Initial Hospital Inpatient Care Services
1013729: Critical Care Services
IMP: Visit: Inpatient Encounter
NONAC: Visit: Inpatient Non-acute
SS: Visit: Short Stay

The next category of covariates includes risk factors/common mental health comorbidities for addictive disease (citation?):
F41.1: Generalized anxiety disorder
F40: Phobic anxiety disorders
F41: Other anxiety disorders
F43: Reaction to severe stress, and adjustment disorders
F32: Depressive episode
F33: Major depressive disorder, recurrent
F31: Bipolar disorder
Z86.59: Personal history of other mental and behavioral disorders
F90: Attention-deficit hyperactivity disorders
F60.3: Borderline personality disorder
F20-F29: Schizophrenia, schizotypal, delusional, and other non-mood psychotic disorders
F60.2: Antisocial personality disorder
Z81.3: Family history of other psychoactive substance abuse and dependence
Z55-Z65: Persons with potential health hazards related to socioeconomic and psychosocial circumstances
F11: Opioid related disorders
F12: Cannabis related disorders
F13: Sedative, hypnotic, or anxiolytic related disorders
F14: Cocaine related disorders
F15: Other stimulant related disorders
F16: Hallucinogen related disorders
F17: Nicotine dependence
F18: Inhalant related disorders
F19: Other psychoactive substance related disorders

The final group of covariates matched for are those for COVID-19 vaccination, which was a list of procedures and one medication indicated by the TriNetX website.

© 2023 Olaker VR et al. *JAMA Network Open.*

91300: Severe acute respiratory syndrome coronavirus 2 (SARS-CoV-2) (Coronavirus disease [COVID-19]) vaccine, mRNA-LNP, spike protein, preservative free, 30 mcg/0.3mL dosage, diluent reconstituted, for intramuscular use
0001A: Immunization administration by intramuscular injection of severe acute respiratory syndrome coronavirus 2 (SARS-CoV-2) (Coronavirus disease [COVID-19]) vaccine, mRNA-LNP, spike protein, preservative free, 30 mcg/0.3mL dosage, diluent reconstituted; first dose
0002A: Immunization administration by intramuscular injection of severe acute respiratory syndrome coronavirus 2 (SARS-CoV-2) (Coronavirus disease [COVID-19]) vaccine, mRNA-LNP, spike protein, preservative free, 30 mcg/0.3mL dosage, diluent reconstituted; second dose
91301: Severe acute respiratory syndrome coronavirus 2 (SARS-CoV-2) (Coronavirus disease [COVID-19]) vaccine, mRNA-LNP, spike protein, preservative free, 100 mcg/0.5mL dosage, for intramuscular use
0011A: Immunization administration by intramuscular injection of severe acute respiratory syndrome coronavirus 2 (SARS-CoV-2) (Coronavirus disease [COVID-19]) vaccine, mRNA-LNP, spike protein, preservative free, 100 mcg/0.5mL dosage; first dose
0012A: Immunization administration by intramuscular injection of severe acute respiratory syndrome coronavirus 2 (SARS-CoV-2) (Coronavirus disease [COVID-19]) vaccine, mRNA-LNP, spike protein, preservative free, 100 mcg/0.5mL dosage; second dose
91302: Severe acute respiratory syndrome coronavirus 2 (SARS-CoV-2) (coronavirus disease [COVID-19]) vaccine, DNA, spike protein, chimpanzee adenovirus Oxford 1 (ChAdOx1) vector, preservative free, 5x10^10 viral particles/0.5mL dosage, for intramuscular use
0021A: Immunization administration by intramuscular injection of severe acute respiratory syndrome coronavirus 2 (SARS-CoV-2) (coronavirus disease [COVID-19]) vaccine, DNA, spike protein, chimpanzee adenovirus Oxford 1 (ChAdOx1) vector, preservative free, 5x10^10 viral particles/0.5mL dosage; first dose
0022A: Immunization administration by intramuscular injection of severe acute respiratory syndrome coronavirus 2 (SARS-CoV-2) (coronavirus disease [COVID-19]) vaccine, DNA, spike protein, chimpanzee adenovirus Oxford 1 (ChAdOx1) vector, preservative free, 5x10^10 viral particles/0.5mL dosage; second dose
91303: Severe acute respiratory syndrome coronavirus 2 (SARS-CoV-2) (coronavirus disease [COVID-19]) vaccine, DNA, spike protein, adenovirus type 26 (Ad26) vector, preservative free, 5x10^10 viral particles/0.5mL dosage, for intramuscular use
0031A: Immunization administration by intramuscular injection of severe acute respiratory syndrome coronavirus 2 (SARS-CoV-2) (coronavirus disease [COVID-19]) vaccine, DNA, spike protein, adenovirus type 26 (Ad26) vector, preservative free, 5x10^10 viral particles/0.5mL dosage, single dose
XW013S6: Introduction of COVID-19 Vaccine Dose 1 into Subcutaneous Tissue, Percutaneous Approach, New Technology Group 6
XW013T6: Introduction of COVID-19 Vaccine Dose 2 into Subcutaneous Tissue, Percutaneous Approach, New Technology Group 6
XW013U6: Introduction of COVID-19 Vaccine into Subcutaneous Tissue, Percutaneous Approach, New Technology Group 6
XW023U6: Introduction of COVID-19 Vaccine into Muscle, Percutaneous Approach, New Technology Group 6
XW023T6: Introduction of COVID-19 Vaccine Dose 2 into Muscle, Percutaneous Approach, New Technology Group 6
XW023S6: Introduction of COVID-19 Vaccine Dose 1 into Muscle, Percutaneous Approach, New Technology Group 6
213: SARS-CoV-2 (COVID-19) Vaccine

© 2023 Olaker VR et al. *JAMA Network Open.*

Details on statistical analyses

The status of SARS-CoV-2 infection was based on the International Classification of Diseases (ICD-10) diagnosis of U07.1. The outcome measure of alcohol use disorder was determined by the presence of a new diagnosis of an alcohol-related disorder (F10). SARS-CoV-2 infections were recorded for patients aged 12 years of age and older. Each COVID-19 cohort was matched with a cohort of patients without SARS-CoV2 infection by the TriNetX built-in propensity score matching function (1:1 matching using a nearest neighbor greedy matching algorithm with a caliper of 0.25 times the standard deviation). Risk of new AUD diagnosis following date of infection was then compared for the SARS-CoV2 cohort to the other respiratory infection cohort using hazard ratios and 95% confidence intervals. Kaplan-Meier analysis was used to estimate the probability of clinical outcomes. Cox's proportional hazards model was used to compare the two matched cohorts. The proportional hazard assumption was tested using the generalized Schoenfeld approach. In our main analyses, there was a single violation of proportionality in block 5 for 3-6 months of follow-up with a p of 0.0048. The TriNetX Platform calculates the hazard ratios and associated confidence intervals, using R's Survival package v3.2-3. For generating hazard ratios, TriNetX sets robust=FALSE using the R survival package, but it does not take into account potential clustering of COVID-19 cases within the healthcare organizations or specific geolocations, a potential weakness or confounding factor in the analysis.

Additional cohorts were run to test the robustness of the findings presented. For one, COVID-19 cohorts were defined differently to include positive COVID-19 tests (using rollup lab code 9088 for any positive RNA test ever) in addition to the option of a COVID-19 diagnosis (U07.1). Blocks four and eight of these analyses had to be split into two smaller time blocks because the additions of the 9088 code for inclusion made these cohorts too large to be run with the original index event timeframes. Block 4 was originally set for index events that occurred from 10/23/20-1/23/20. To reduce the cohort size, two cohorts were made to capture this time frame. Block 4 Part 1 indexes diagnoses from 10/23/20 and 12/04/20. Block 4 Part 2 indexes diagnoses from 12/05/21 to 1/23/21. Block 8 was originally set for index events that occurred from 10/27/21-10/27/22. To reduce the cohort size, two cohorts were made to capture this time frame. Block 8 Part 1 indexes diagnoses from 10/27/21 to 12/8/21. Block 8 Part 2 indexes diagnoses from 12/09/21-1/27/22. These results are displayed in figures 1 and 2. These analyses gave a similar pattern of HRs compared to the main analyses that only used U07.1, but they were slightly lower than those for the U07.1 cohorts only.

Another set of analyses was run by changing the control cohorts to the index event of large bone fractures (adapted from Taquet et al.) rather than other respiratory infections. The codes used for inclusion were any of the following:
S32: Fracture of lumbar spine and pelvis
S42: Fracture of shoulder and upper arm
S52: Fracture of forearm
F72: Fracture of femur
S82: Fracture of lower leg, including ankle
These analyses also yielded similar temporal results to those presented in the analyses and are shown in figures 3 and 4.

Lastly, cohorts for COVID-19 vs ORI were run in delta and omicron specific time periods to see if these waves of COVID-19 showed any break from the HR trend observed in the three-month blocks of time that we originally ran. These results are presented in figures 5 and 6. These results showed no difference in risk of first diagnosis of an alcohol-related disorder.

Characteristics for all cohorts
Table 1 in the article presented baseline characteristics for the block 1 cohorts. This table was additionally reduced to only show demographics, risk factors, and common comorbidities for addictive disease. An

© 2023 Olaker VR et al. *JAMA Network Open.*

excel file that is part of the online supplementary material contains the data for all covariates matched for blocks 1-8 for the COVID-19 vs. ORI cohort analyses.

© 2023 Olaker VR et al. *JAMA Network Open.*

eFigure 1.

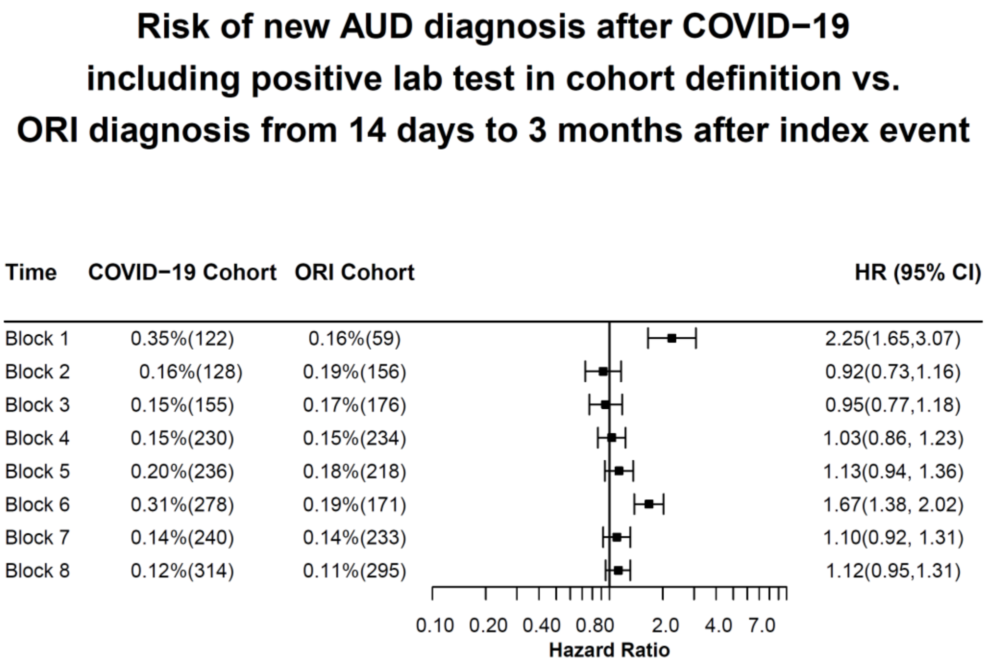

eFigure 1. This plot shows the HRs and 95% CIs when COVID-19 groups that had the additional inclusion criteria of a positive COVID-19 test were compared to the same ORI cohorts used in the main analysis. The follow-up period for these results is from 14 days to 3 months after the index event, which is a diagnosis or positive test of COVID-19 for the exposure group and diagnosis of another respiratory infection for the control group.

© 2023 Olaker VR et al. *JAMA Network Open.*

eFigure 2.

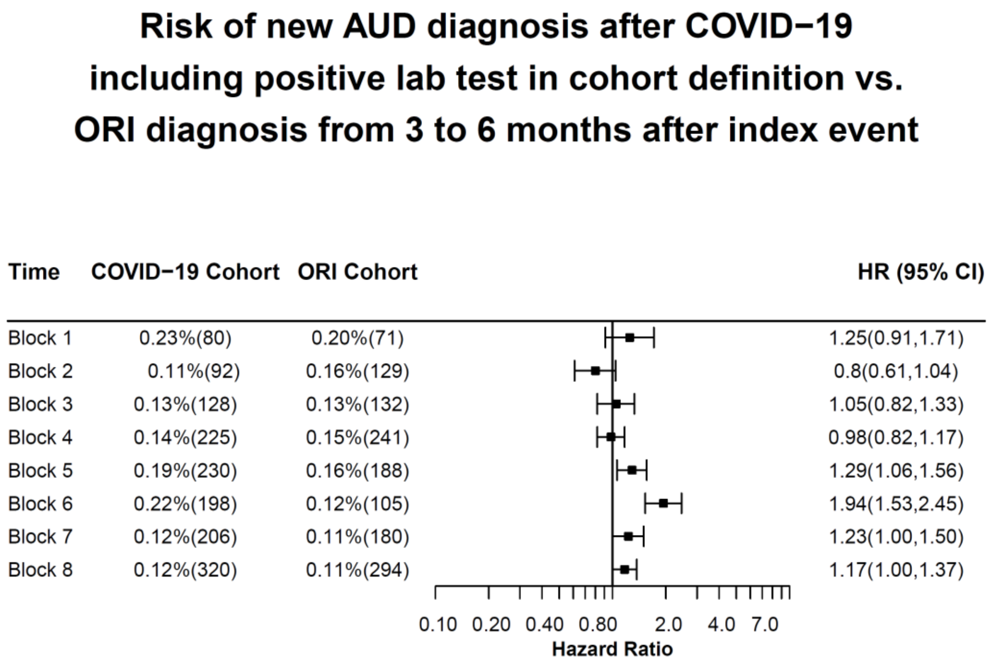

eFigure 2. This plot shows the HRs and 95% CIs when COVID-19 groups that had the additional inclusion criteria of a positive COVID-19 test were compared to the same ORI cohorts used in the main analysis. The follow-up period for these results is from 3 to 6 months after the index event, which is a diagnosis or positive test of COVID-19 for the exposure group and diagnosis of another respiratory infection for the control group.

eFigure 3.

Risk of new AUD diagnosis after COVID−19 diagnosis vs. bone fracture diagnosis from 14 days to 3 months after index event

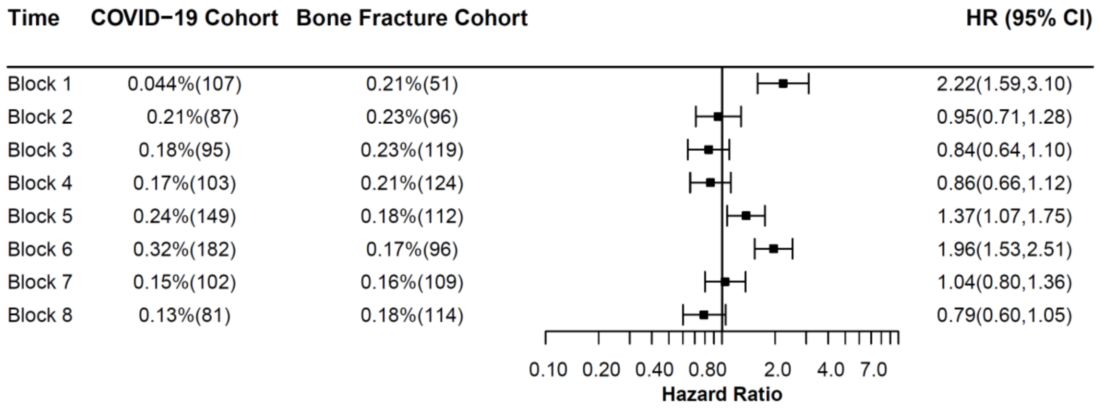

eFigure 3. This plot shows the HRs and 95% CIs when COVID-19 were compared to control cohorts defined by a large bone fracture (adapted from Taquet et al.). The follow-up period for these results is from 14 days to 3 months after the index event, which is a diagnosis of COVID-19 for the exposure group and diagnosis of another respiratory infection for the control group.

© 2023 Olaker VR et al. *JAMA Network Open.*

eFigure 4.

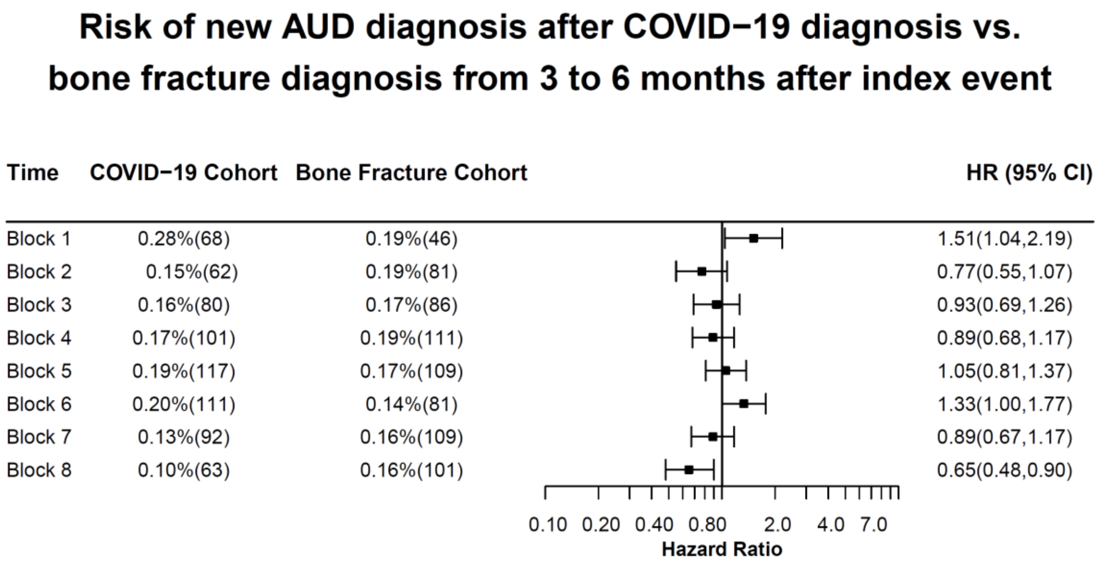

eFigure 4. This plot shows the HRs and 95% CIs when COVID-19 were compared to control cohorts defined by a large bone fracture (adapted from Taquet et al.). The follow-up period for these results is from 3 to 6 months after the index event, which is a diagnosis of COVID-19 for the exposure group and diagnosis of another respiratory infection for the control group.

© 2023 Olaker VR et al. *JAMA Network Open.*

eFigure 5.

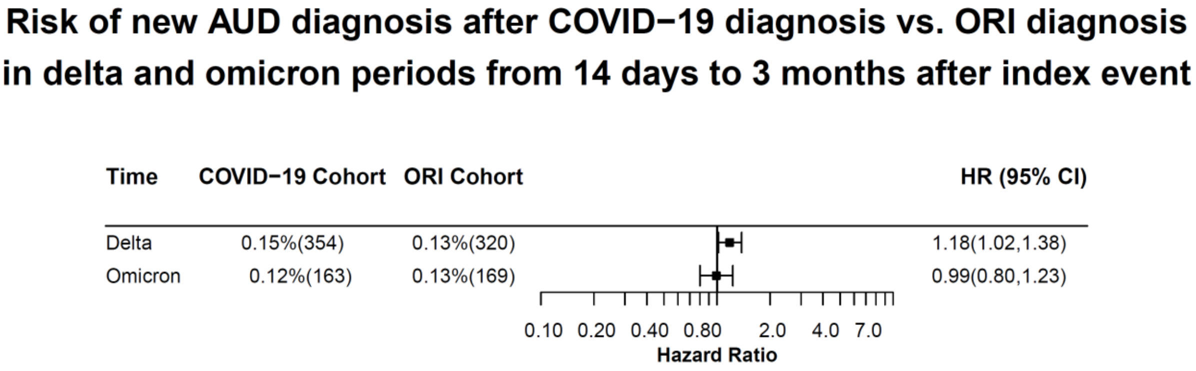

eFigure 5. This plot shows the HRs and 95% CIs when COVID-19 were compared to ORI control cohorts, both indexed in the delta and omicron time periods. The follow-up period for these results is from 14 days to 3 months after the index event, which is a diagnosis of COVID-19 for the exposure group and diagnosis of another respiratory infection for the control group.

© 2023 Olaker VR et al. *JAMA Network Open.*

eFigure 6.

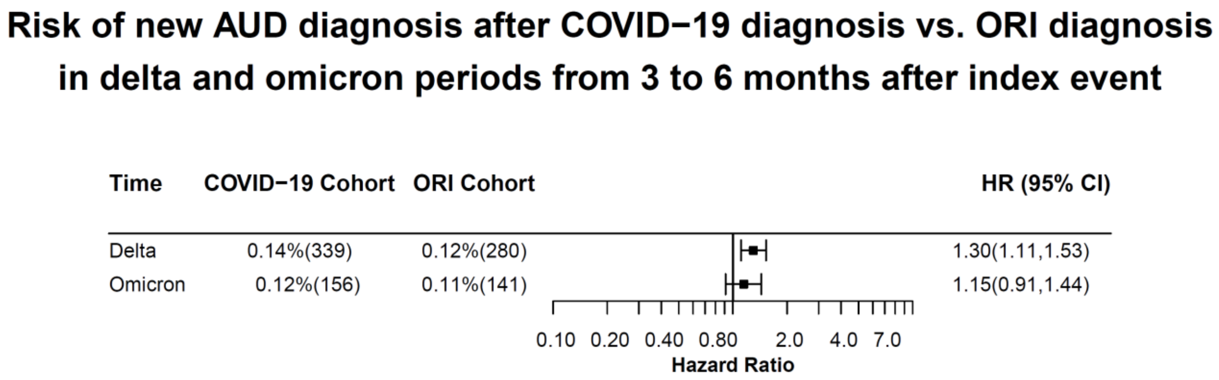

eFigure 6. This plot shows the HRs and 95% CIs when COVID-19 were compared to ORI control cohorts, both indexed in the delta and omicron time periods. The follow-up period for these results is from 3 to 6 months after the index event, which is a diagnosis of COVID-19 for the exposure group and diagnosis of another respiratory infection for the control group.

© 2023 Olaker VR et al. *JAMA Network Open.*

eTable 1. Baseline Characteristics for Block 1 Before and After Matching

	Before matching			After matching		
	Cohort, No. (%)			Cohort, No. (%)		
Characteristics	COVID-19	ORI	SMD	COVID-19	ORI	SMD
Total number	30253	383,136		30250	30250	
Current Age, mean (SD), y	52 (17.6)	46.8 (19.8)	0.27	51.9 (17.6)	52.5 (18.7)	0.032
Age at Index, mean (SD), y	49.2 (17.6)	44.0 (19.9)	0.28	49.2 (17.6)	49.8 (18.7)	0.031
Gender						
Female	16655 (55.1)	229644 (59.9)	0.10	16654 (55.1)	16647 (55)	0.000
Male	13429 (44.4)	143244 (37.4)	0.14	13427 (44.4)	13434 (44.4)	0.000
Unknown	169 (0.6)	10248 (2.7)	0.17	169 (0.6)	169 (0.6)	0.000
Ethnicity						
Hispanic or Latino	4430 (14.6)	33360 (8.7)	0.19	4429 (14.6)	4723 (15.6)	0.027
Not Hispanic or Latino	17164 (56.7)	269091 (70.2)	0.28	17162 (56.7)	17258 (57.1)	0.006
Unknown	8659 (28.6)	80685 (21.1)	0.18	8659 (28.6)	8269 (27.3)	0.029
Race						
Asian	1010 (3.3)	10184 (2.7)	0.04	1009 (3.3)	970 (3.2)	0.007
Black or African American	7828 (25.9)	59195 (15.5)	0.26	7827 (25.9)	7500 (24.8)	0.025
Native American or Alaska Native	100 (0.3)	1188 (0.3)	0.00	100 (0.3)	100 (0.3)	0.000
Native Hawaiian or Other Pacific Islander	68 (0.2)	564 (0.1)	0.02	68 (0.2)	53 (0.2)	0.011
Unknown	6408 (21.2)	45472 (11.9)	0.25	6407 (21.2)	6876 (22.7)	0.037
White	14839 (49.1)	266533 (69.6)	0.43	14839 (49.1)	14751 (48.8)	0.006
Hypertensive diseases	10434 (34.5)	113315 (29.6)	0.11	10432 (34.5)	9834 (32.5)	0.042
Neoplasms	7726 (25.5)	88263 (23)	0.06	7723 (25.5)	7236 (23.9)	0.037
Other forms of heart disease	6448 (21.3)	63509 (16.6)	0.12	6445 (21.3)	6042 (20)	0.033
Overweight and obesity	6318 (20.9)	72684 (19)	0.05	6317 (20.9)	5927 (19.6)	0.032
Type 2 diabetes mellitus	5866 (19.4)	49192 (12.8)	0.18	5864 (19.4)	5416 (17.9)	0.038
Other anxiety disorders	5495 (18.2)	84732 (22.1)	0.10	5493 (18.2)	5200 (17.2)	0.025
Depressive episode	5087 (16.8)	63772 (16.6)	0.00	5085 (16.8)	4676 (15.5)	0.037
Asthma	4061 (13.4)	59374 (15.5)	0.06	4060 (13.4)	3883 (12.8)	0.017
Nicotine dependence	3416 (11.3)	45028 (11.8)	0.01	3415 (11.3)	3172 (10.5)	0.026
Persons with potential health hazards related to socioeconomic and psychosocial circumstances	2053 (6.8)	16031 (4.2)	0.11	2051 (6.8)	1801 (6)	0.034
Cerebral infarction	1827 (6)	11028 (2.9)	0.15	1826 (6)	1580 (5.2)	0.035
Reaction to severe stress, and adjustment disorders	1691 (5.6)	22340 (5.8)	0.01	1691 (5.6)	1567 (5.2)	0.018
Other chronic obstructive pulmonary disease	1686 (5.6)	22510 (5.9)	0.01	1686 (5.6)	1566 (5.2)	0.018

© 2023 Olaker VR et al. *JAMA Network Open.*

Generalized anxiety disorder	1590 (5.3)	25250 (6.6)	0.06	1590 (5.3)	1478 (4.9)	0.017
Bronchitis, not specified as acute or chronic	1508 (5)	30651 (8)	0.12	1506 (5)	1494 (4.9)	0.002
Hypertensive chronic kidney disease	1386 (4.6)	9385 (2.5)	0.12	1384 (4.6)	1208 (4)	0.029
Other psychoactive substance related disorders	1349 (4.5)	8344 (2.2)	0.13	1346 (4.5)	1199 (4)	0.024
Major depressive disorder, recurrent	1335 (4.4)	16911 (4.4)	0.00	1334 (4.4)	1219 (4)	0.019
Certain disorders involving the immune mechanism	1067 (3.5)	10214 (2.7)	0.05	1067 (3.5)	1011 (3.3)	0.010
Fatty (change of) liver, not elsewhere classified	1011 (3.3)	12214 (3.2)	0.01	1011 (3.3)	961 (3.2)	0.009
Schizophrenia, schizotypal, delusional, and other non-mood psychotic disorders	869 (2.9)	5289 (1.4)	0.10	868 (2.9)	810 (2.7)	0.012
Type 1 diabetes mellitus	820 (2.7)	6809 (1.8)	0.06	819 (2.7)	738 (2.4)	0.017
Attention-deficit hyperactivity disorders	715 (2.4)	12972 (3.4)	0.06	715 (2.4)	661 (2.2)	0.012
Other specified diseases of liver	709 (2.3)	8398 (2.2)	0.01	709 (2.3)	649 (2.1)	0.013
Bipolar disorder	686 (2.3)	8045 (2.1)	0.01	686 (2.3)	657 (2.2)	0.007
Emphysema	682 (2.3)	8945 (2.3)	0.01	681 (2.3)	616 (2)	0.015
Malignant neoplasms of lymphoid, hematopoietic and related tissue	665 (2.2)	5574 (1.5)	0.06	665 (2.2)	628 (2.1)	0.008
Personal history of other mental and behavioral disorders	628 (2.1)	4455 (1.2)	0.07	626 (2.1)	586 (1.9)	0.009
Other rheumatoid arthritis	594 (2)	6957 (1.8)	0.01	594 (2)	537 (1.8)	0.014
Phobic anxiety disorders	540 (1.8)	6434 (1.7)	0.01	540 (1.8)	505 (1.7)	0.009
Unspecified dementia	531 (1.8)	2517 (0.7)	0.10	530 (1.8)	474 (1.6)	0.014
Cannabis related disorders	526 (1.7)	5430 (1.4)	0.03	526 (1.7)	482 (1.6)	0.011
Other stimulant related disorders	471 (1.6)	3079 (0.8)	0.07	470 (1.6)	436 (1.4)	0.009
Opioid related disorders	446 (1.5)	4857 (1.3)	0.02	446 (1.5)	380 (1.3)	0.019
Fibrosis and cirrhosis of liver	431 (1.4)	3263 (0.9)	0.05	430 (1.4)	351 (1.2)	0.023
Psoriasis	414 (1.4)	5917 (1.5)	0.01	414 (1.4)	389 (1.3)	0.007
Systemic lupus erythematosus (SLE)	282 (0.9)	2688 (0.7)	0.03	281 (0.9)	239 (0.8)	0.015
Bronchiectasis	250 (0.8)	3328 (0.9)	0.00	250 (0.8)	240 (0.8)	0.004
Unspecified chronic bronchitis	216 (0.7)	3094 (0.8)	0.01	216 (0.7)	189 (0.6)	0.011
Dementia in other diseases classified elsewhere	212 (0.7)	1208 (0.3)	0.05	212 (0.7)	180 (0.6)	0.013
Alzheimer's disease	183 (0.6)	993 (0.3)	0.05	183 (0.6)	159 (0.5)	0.011
Family history of other psychoactive substance abuse and dependence	176 (0.6)	584 (0.2)	0.07	175 (0.6)	146 (0.5)	0.013
Cocaine related disorders	175 (0.6)	1779 (0.5)	0.02	175 (0.6)	151 (0.5)	0.011
Rheumatoid arthritis with rheumatoid factor	173 (0.6)	2211 (0.6)	0.00	173 (0.6)	158 (0.5)	0.007
Hepatic failure, not elsewhere classified	172 (0.6)	1217 (0.3)	0.04	172 (0.6)	149 (0.5)	0.010
Chronic passive congestion of liver	164 (0.5)	2018 (0.5)	0.00	164 (0.5)	142 (0.5)	0.010

© 2023 Olaker VR et al. *JAMA Network Open.*

Simple and mucopurulent chronic bronchitis	151 (0.5)	2777 (0.7)	0.03	151 (0.5)	151 (0.5)	0.000
Vascular dementia	140 (0.5)	713 (0.2)	0.05	140 (0.5)	115 (0.4)	0.013
Portal hypertension	129 (0.4)	1005 (0.3)	0.03	128 (0.4)	103 (0.3)	0.013
Hallucinogen related disorders	119 (0.4)	557 (0.1)	0.05	117 (0.4)	83 (0.3)	0.020
Borderline personality disorder	116 (0.4)	1031 (0.3)	0.02	116 (0.4)	99 (0.3)	0.009
Inhalant related disorders	96 (0.3)	1371 (0.4)	0.01	96 (0.3)	103 (0.3)	0.004
Sedative, hypnotic, or anxiolytic related disorders	94 (0.3)	922 (0.2)	0.01	94 (0.3)	63 (0.2)	0.020
Chronic hepatitis, not elsewhere classified	65 (0.2)	454 (0.1)	0.02	64 (0.2)	49 (0.2)	0.011
Dementia with Lewy bodies	26 (0.1)	122 (0)	0.02	26 (0.1)	22 (0.1)	0.005
Frontotemporal dementia	21 (0.1)	79 (0)	0.02	21 (0.1)	19 (0.1)	0.003
Antisocial personality disorder	21 (0.1)	90 (0)	0.02	21 (0.1)	22 (0.1)	0.001
Blood Pressure, Systolic	16728 (55.3)	212903 (55.6)	0.06	16725 (55.3)	15310 (50.6)	0.012
Blood Pressure, Systolic, at least 160 mm [Hg]	4609 (15.2)	52910 (13.8)	0.04	4607 (15.2)	4306 (14.2)	0.028
Blood Pressure, Systolic, 140-160 mm [Hg]	9099 (30.1)	108078 (28.2)	0.04	9096 (30.1)	8415 (27.8)	0.050
Blood Pressure, Systolic, At most 140 mm [Hg]	15855 (52.4)	206277 (53.8)	0.03	15852 (52.4)	14712 (48.6)	0.075
Blood Pressure, Diastolic	16537 (54.7)	212345 (55.4)	0.09	16534 (54.7)	15244 (50.4)	0.060
Blood Pressure, Diastolic, at least 100 mm [Hg]	3429 (11.3)	37575 (9.8)	0.05	3427 (11.3)	3149 (10.4)	0.030
Blood Pressure, Diastolic, 90-100 mm [Hg]	6908 (22.8)	83328 (21.7)	0.03	6906 (22.8)	6416 (21.2)	0.039
Blood Pressure, Diastolic, At most 90 mm [Hg]	16087 (53.2)	209114 (54.6)	0.03	16084 (53.2)	14948 (49.4)	0.075
BMI	12486 (41.3)	160434 (41.9)	0.15	12484 (41.3)	11663 (38.6)	0.043
BMI, at least 30 kg/m2	6820 (22.5)	75560 (19.7)	0.07	6819 (22.5)	6311 (20.9)	0.041
BMI, 25-30 kg/m2	6025 (19.9)	74209 (19.4)	0.01	6023 (19.9)	5691 (18.8)	0.028
BMI, At most 25 kg/m2	4997 (16.5)	74200 (19.4)	0.07	4995 (16.5)	4580 (15.1)	0.038
Hospital Inpatient Services	3471 (11.5)	36287 (9.5)	0.07	3469 (11.5)	3189 (10.5)	0.030
New or Established Patient Initial Hospital Inpatient Care Services	2870 (9.5)	29191 (7.6)	0.07	2868 (9.5)	2595 (8.6)	0.031
Critical Care Services	1253 (4.1)	9736 (2.5)	0.09	1251 (4.1)	1134 (3.7)	0.020
Renal Transplantation Procedures	57 (0.2)	280 (0.1)	0.03	57 (0.2)	58 (0.2)	0.001
Liver Transplantation Procedures	10 (0)	73 (0)	0.01	10 (0)	10 (0)	0.000
Severe acute respiratory syndrome coronavirus 2 (SARS-CoV-2) (Coronavirus disease [COVID-19]) vaccine, mRNA-LNP, spike protein, preservative free, 30 mcg/0.3mL dosage, diluent reconstituted, for intramuscular use	0 (0)	0 (0)		0 (0)	0 (0)	

© 2023 Olaker VR et al. *JAMA Network Open.*

Immunization administration by intramuscular injection of severe acute respiratory syndrome coronavirus 2 (SARS-CoV-2) (Coronavirus disease [COVID-19]) vaccine, mRNA-LNP, spike protein, preservative free, 30 mcg/0.3mL dosage, diluent reconstituted; first dose	0 (0)	0 (0)		0 (0)	0 (0)	
Immunization administration by intramuscular injection of severe acute respiratory syndrome coronavirus 2 (SARS-CoV-2) (Coronavirus disease [COVID-19]) vaccine, mRNA-LNP, spike protein, preservative free, 30 mcg/0.3mL dosage, diluent reconstituted; second dose	0 (0)	0 (0)		0 (0)	0 (0)	
Severe acute respiratory syndrome coronavirus 2 (SARS-CoV-2) (Coronavirus disease [COVID-19]) vaccine, mRNA-LNP, spike protein, preservative free, 100 mcg/0.5mL dosage, for intramuscular use	0 (0)	0 (0)		0 (0)	0 (0)	
Immunization administration by intramuscular injection of severe acute respiratory syndrome coronavirus 2 (SARS-CoV-2) (Coronavirus disease [COVID-19]) vaccine, mRNA-LNP, spike protein, preservative free, 100 mcg/0.5mL dosage; first dose	0 (0)	0 (0)		0 (0)	0 (0)	
Immunization administration by intramuscular injection of severe acute respiratory syndrome coronavirus 2 (SARS-CoV-2) (Coronavirus disease [COVID-19]) vaccine, mRNA-LNP, spike protein, preservative free, 100 mcg/0.5mL dosage; second dose	0 (0)	0 (0)		0 (0)	0 (0)	
Severe acute respiratory syndrome coronavirus 2 (SARS-CoV-2) (coronavirus disease [COVID-19]) vaccine, DNA, spike protein, chimpanzee adenovirus Oxford 1 (ChAdOx1) vector, preservative free, 5x10^10 viral particles/0.5mL dosage, for intramuscular use	0 (0)	0 (0)		0 (0)	0 (0)	

© 2023 Olaker VR et al. *JAMA Network Open.*

Immunization administration by intramuscular injection of severe acute respiratory syndrome coronavirus 2 (SARS-CoV-2) (coronavirus disease [COVID-19]) vaccine, DNA, spike protein, chimpanzee adenovirus Oxford 1 (ChAdOx1) vector, preservative free, 5x10^10 viral particles/0.5mL dosage; first dose	0 (0)	0 (0)		0 (0)	0 (0)	
Immunization administration by intramuscular injection of severe acute respiratory syndrome coronavirus 2 (SARS-CoV-2) (coronavirus disease [COVID-19]) vaccine, DNA, spike protein, chimpanzee adenovirus Oxford 1 (ChAdOx1) vector, preservative free, 5x10^10 viral particles/0.5mL dosage; second dose	0 (0)	0 (0)		0 (0)	0 (0)	
Severe acute respiratory syndrome coronavirus 2 (SARS-CoV-2) (coronavirus disease [COVID-19]) vaccine, DNA, spike protein, adenovirus type 26 (Ad26) vector, preservative free, 5x10^10 viral particles/0.5mL dosage, for intramuscular use	0 (0)	0 (0)		0 (0)	0 (0)	
Immunization administration by intramuscular injection of severe acute respiratory syndrome coronavirus 2 (SARS-CoV-2) (coronavirus disease [COVID-19]) vaccine, DNA, spike protein, adenovirus type 26 (Ad26) vector, preservative free, 5x10^10 viral particles/0.5mL dosage, single dose	0 (0)	0 (0)		0 (0)	0 (0)	
Introduction of COVID-19 Vaccine Dose 1 into Subcutaneous Tissue, Percutaneous Approach, New Technology Group 6	0 (0)	0 (0)		0 (0)	0 (0)	
Introduction of COVID-19 Vaccine Dose 2 into Subcutaneous Tissue, Percutaneous Approach, New Technology Group 6	0 (0)	0 (0)		0 (0)	0 (0)	
Introduction of COVID-19 Vaccine into Subcutaneous Tissue, Percutaneous Approach, New Technology Group 6	0 (0)	0 (0)		0 (0)	0 (0)	
Introduction of COVID-19 Vaccine into Muscle, Percutaneous Approach, New Technology Group 6	0 (0)	0 (0)		0 (0)	0 (0)	

© 2023 Olaker VR et al. *JAMA Network Open.*

Introduction of COVID-19 Vaccine Dose 2 into Muscle, Percutaneous Approach, New Technology Group 6	0 (0)	0 (0)		0 (0)	0 (0)	
Introduction of COVID-19 Vaccine Dose 1 into Muscle, Percutaneous Approach, New Technology Group 6	0 (0)	0 (0)		0 (0)	0 (0)	
Visit: Inpatient Encounter	7368 (24.4)	89775 (23.4)	0.02	7365 (24.3)	6757 (22.3)	0.048
Visit: Short Stay	1291 (4.3)	6992 (1.8)	0.14	1289 (4.3)	1156 (3.8)	0.022
Visit: Inpatient Non-acute	37 (0.1)	1380 (0.4)	0.05	37 (0.1)	45 (0.1)	0.007
SARS-CoV-2 (COVID-19) Vaccine	0 (0)	10 (0)	0.01	0 (0)	0 (0)	

© 2023 Olaker VR et al. *JAMA Network Open.*

eTable 2. Baseline Characteristics for Block 2 Before and After Matching

	Before matching			After matching		
	Cohort , No. (%)			Cohort , No. (%)		
Characteristic Name	COVID-19	ORI	SMD	COVID-19	ORI	SMD
Total number	79986	124289		71819	71819	
Current Age, mean (SD), y	48.1 (18.2)	49.7 (19.6)	0.08	48.3 (18.3)	48.1 (19.2)	0.01
Age at Index, mean (SD), y	45.6 (18.2)	47.2 (19.6)	0.08	45.8 (18.3)	45.6 (19.2)	0.01
Gender						
Female	45025 (56.3)	75441 (60.7)	0.09	41116 (57.2)	41265 (57.5)	0.00
Male	33915 (42.4)	46202 (37.2)	0.11	29659 (41.3)	29560 (41.2)	0.00
Unknown	1046 (1.3)	2646 (2.1)	0.06	1044 (1.5)	994 (1.4)	0.01
Ethnicity						
Hispanic or Latino	17170 (21.5)	11311 (9.1)	0.35	10812 (15.1)	10870 (15.1)	0.00
Not Hispanic or Latino	17261 (21.6)	24766 (19.9)	0.04	16287 (22.7)	16337 (22.7)	0.00
Unknown	45555 (57)	88212 (71)	0.30	44720 (62.3)	44612 (62.1)	0.00
Race						
Asian	2068 (2.6)	2819 (2.3)	0.02	2004 (2.8)	2038 (2.8)	0.00
Black or African American	17728 (22.2)	21195 (17.1)	0.13	16314 (22.7)	16452 (22.9)	0.00
Native American or Alaska Native	302 (0.4)	416 (0.3)	0.01	262 (0.4)	262 (0.4)	0.00
Native Hawaiian or Other Pacific Islander	235 (0.3)	189 (0.2)	0.03	182 (0.3)	178 (0.2)	0.00
Unknown	16412 (20.5)	16527 (13.3)	0.19	12872 (17.9)	12989 (18.1)	0.00
White	43241 (54.1)	83143 (66.9)	0.26	40185 (56)	39900 (55.6)	0.01
Hypertensive diseases	21944 (27.4)	41867 (33.7)	0.14	20311 (28.3)	19958 (27.8)	0.01
Neoplasms	16735 (20.9)	33174 (26.7)	0.14	15666 (21.8)	15365 (21.4)	0.01
Overweight and obesity	14220 (17.8)	24323 (19.6)	0.05	12820 (17.9)	12656 (17.6)	0.01
Other forms of heart disease	12810 (16)	26022 (20.9)	0.13	11954 (16.6)	11775 (16.4)	0.01
Other anxiety disorders	12061 (15.1)	30592 (24.6)	0.24	11595 (16.1)	11542 (16.1)	0.00
Type 2 diabetes mellitus	12189 (15.2)	18413 (14.8)	0.01	10585 (14.7)	10410 (14.5)	0.01
Depressive episode	10919 (13.7)	23989 (19.3)	0.15	10253 (14.3)	10154 (14.1)	0.00
Asthma	8409 (10.5)	20227 (16.3)	0.17	8085 (11.3)	8145 (11.3)	0.00
Nicotine dependence	7257 (9.1)	16496 (13.3)	0.13	6877 (9.6)	6854 (9.5)	0.00
Persons with potential health hazards related to socioeconomic and psychosocial circumstances	4143 (5.2)	5716 (4.6)	0.03	3575 (5)	3568 (5)	0.00
Reaction to severe stress, and adjustment disorders	3533 (4.4)	8105 (6.5)	0.09	3327 (4.6)	3350 (4.7)	0.00
Generalized anxiety disorder	3379 (4.2)	9264 (7.5)	0.14	3259 (4.5)	3319 (4.6)	0.00
Bronchitis, not specified as acute or chronic	3238 (4)	10078 (8.1)	0.17	3169 (4.4)	3269 (4.6)	0.01
Cerebral infarction	3278 (4.1)	4509 (3.6)	0.02	2804 (3.9)	2830 (3.9)	0.00
Other chronic obstructive pulmonary disease	2803 (3.5)	10578 (8.5)	0.21	2772 (3.9)	2861 (4)	0.01
Major depressive disorder, recurrent	2722 (3.4)	6676 (5.4)	0.10	2582 (3.6)	2600 (3.6)	0.00
Fatty (change of) liver, not elsewhere classified	2636 (3.3)	4831 (3.9)	0.03	2305 (3.2)	2304 (3.2)	0.00

© 2023 Olaker VR et al. *JAMA Network Open.*

Hypertensive chronic kidney disease	2399 (3)	4478 (3.6)	0.03	2167 (3)	2149 (3)	0.00
Other psychoactive substance related disorders	2196 (2.7)	3606 (2.9)	0.01	1921 (2.7)	1927 (2.7)	0.00
Certain disorders involving the immune mechanism	2071 (2.6)	4468 (3.6)	0.06	1913 (2.7)	1899 (2.6)	0.00
Attention-deficit hyperactivity disorders	1643 (2.1)	3828 (3.1)	0.06	1576 (2.2)	1578 (2.2)	0.00
Other specified diseases of liver	1624 (2)	3625 (2.9)	0.06	1503 (2.1)	1464 (2)	0.00
Type 1 diabetes mellitus	1555 (1.9)	2663 (2.1)	0.01	1378 (1.9)	1370 (1.9)	0.00
Bipolar disorder	1426 (1.8)	3364 (2.7)	0.06	1365 (1.9)	1342 (1.9)	0.00
Schizophrenia, schizotypal, delusional, and other non-mood psychotic disorders	1513 (1.9)	2311 (1.9)	0.00	1336 (1.9)	1324 (1.8)	0.00
Emphysema	1197 (1.5)	4494 (3.6)	0.13	1175 (1.6)	1237 (1.7)	0.01
Other rheumatoid arthritis	1182 (1.5)	3018 (2.4)	0.07	1146 (1.6)	1185 (1.7)	0.00
Personal history of other mental and behavioral disorders	1267 (1.6)	1763 (1.4)	0.01	1108 (1.5)	1080 (1.5)	0.00
Cannabis related disorders	1164 (1.5)	2187 (1.8)	0.02	1075 (1.5)	1083 (1.5)	0.00
Malignant neoplasms of lymphoid, hematopoietic and related tissue	1084 (1.4)	2453 (2)	0.05	1036 (1.4)	1034 (1.4)	0.00
Psoriasis	891 (1.1)	2118 (1.7)	0.05	854 (1.2)	830 (1.2)	0.00
Phobic anxiety disorders	907 (1.1)	2117 (1.7)	0.05	835 (1.2)	802 (1.1)	0.00
Fibrosis and cirrhosis of liver	856 (1.1)	1578 (1.3)	0.02	777 (1.1)	801 (1.1)	0.00
Opioid related disorders	783 (1)	2381 (1.9)	0.08	763 (1.1)	794 (1.1)	0.00
Unspecified dementia	887 (1.1)	1130 (0.9)	0.02	755 (1.1)	748 (1)	0.00
Other stimulant related disorders	908 (1.1)	974 (0.8)	0.04	745 (1)	708 (1)	0.01
Systemic lupus erythematosus (SLE)	510 (0.6)	1188 (1)	0.04	489 (0.7)	511 (0.7)	0.00
Bronchiectasis	421 (0.5)	1881 (1.5)	0.10	418 (0.6)	493 (0.7)	0.01
Chronic passive congestion of liver	400 (0.5)	969 (0.8)	0.04	378 (0.5)	372 (0.5)	0.00
Unspecified chronic bronchitis	363 (0.5)	1523 (1.2)	0.08	355 (0.5)	351 (0.5)	0.00
Rheumatoid arthritis with rheumatoid factor	354 (0.4)	920 (0.7)	0.04	344 (0.5)	353 (0.5)	0.00
Cocaine related disorders	357 (0.4)	850 (0.7)	0.03	343 (0.5)	337 (0.5)	0.00
Hepatic failure, not elsewhere classified	355 (0.4)	707 (0.6)	0.02	338 (0.5)	370 (0.5)	0.01
Dementia in other diseases classified elsewhere	355 (0.4)	587 (0.5)	0.00	325 (0.5)	345 (0.5)	0.00
Alzheimer's disease	361 (0.5)	484 (0.4)	0.01	318 (0.4)	323 (0.5)	0.00
Portal hypertension	303 (0.4)	563 (0.5)	0.01	279 (0.4)	299 (0.4)	0.00
Simple and mucopurulent chronic bronchitis	274 (0.3)	1370 (1.1)	0.09	270 (0.4)	318 (0.4)	0.01
Inhalant related disorders	238 (0.3)	625 (0.5)	0.03	232 (0.3)	231 (0.3)	0.00
Borderline personality disorder	222 (0.3)	440 (0.4)	0.01	205 (0.3)	214 (0.3)	0.00
Vascular dementia	238 (0.3)	324 (0.3)	0.01	201 (0.3)	201 (0.3)	0.00
Family history of other psychoactive substance abuse and dependence	248 (0.3)	212 (0.2)	0.03	193 (0.3)	189 (0.3)	0.00

© 2023 Olaker VR et al. *JAMA Network Open.*

Hallucinogen related disorders	165 (0.2)	281 (0.2)	0.00	151 (0.2)	155 (0.2)	0.00
Sedative, hypnotic, or anxiolytic related disorders	141 (0.2)	435 (0.4)	0.03	136 (0.2)	132 (0.2)	0.00
Chronic hepatitis, not elsewhere classified	106 (0.1)	205 (0.2)	0.01	97 (0.1)	86 (0.1)	0.00
Dementia with Lewy bodies	45 (0.1)	50 (0)	0.01	39 (0.1)	35 (0)	0.00
Antisocial personality disorder	30 (0)	37 (0)	0.00	27 (0)	24 (0)	0.00
Frontotemporal dementia	31 (0)	32 (0)	0.01	26 (0)	16 (0)	0.01
Blood Pressure, Systolic	39824 (49.8)	67607 (54.4)	0.01	36300 (50.5)	35450 (49.4)	0.02
Blood Pressure, Systolic, at least 160 mm [Hg]	10219 (12.8)	19956 (16.1)	0.09	9431 (13.1)	9168 (12.8)	0.01
Blood Pressure, Systolic, 140-160 mm [Hg]	20849 (26.1)	38321 (30.8)	0.11	19168 (26.7)	18628 (25.9)	0.02
Blood Pressure, Systolic, At most 140 mm [Hg]	37911 (47.4)	65498 (52.7)	0.11	34597 (48.2)	34077 (47.4)	0.01
Blood Pressure, Diastolic	39312 (49.1)	66935 (53.9)	0.03	35828 (49.9)	35052 (48.8)	0.03
Blood Pressure, Diastolic, at least 100 mm [Hg]	7615 (9.5)	13868 (11.2)	0.05	6955 (9.7)	6791 (9.5)	0.01
Blood Pressure, Diastolic, 90-100 mm [Hg]	15985 (20)	29477 (23.7)	0.09	14665 (20.4)	14279 (19.9)	0.01
Blood Pressure, Diastolic, At most 90 mm [Hg]	38378 (48)	65918 (53)	0.10	34990 (48.7)	34417 (47.9)	0.02
BMI	31634 (39.5)	52836 (42.5)	0.10	28762 (40)	28104 (39.1)	0.04
BMI, at least 30 kg/m2	16493 (20.6)	25719 (20.7)	0.00	14645 (20.4)	14291 (19.9)	0.01
BMI, 25-30 kg/m2	14898 (18.6)	25416 (20.4)	0.05	13558 (18.9)	13324 (18.6)	0.01
BMI, At most 25 kg/m2	12726 (15.9)	24361 (19.6)	0.10	11951 (16.6)	11866 (16.5)	0.00
Hospital Inpatient Services	8416 (10.5)	15638 (12.6)	0.06	7566 (10.5)	7409 (10.3)	0.01
New or Established Patient Initial Hospital Inpatient Care Services	6866 (8.6)	12850 (10.3)	0.06	6163 (8.6)	6009 (8.4)	0.01
Critical Care Services	2377 (3)	4730 (3.8)	0.05	2169 (3)	2118 (2.9)	0.00
Renal Transplantation Procedures	150 (0.2)	134 (0.1)	0.02	124 (0.2)	103 (0.1)	0.01
Liver Transplantation Procedures	29 (0)	41 (0)	0.00	26 (0)	26 (0)	0.00
Severe acute respiratory syndrome coronavirus 2 (SARS-CoV-2) (Coronavirus disease [COVID-19]) vaccine, mRNA-LNP, spike protein, preservative free, 30 mcg/0.3mL dosage, diluent reconstituted, for intramuscular use	0 (0)	0 (0)		0 (0)	0 (0)	
Immunization administration by intramuscular injection of severe acute respiratory syndrome coronavirus 2 (SARS-CoV-2) (Coronavirus disease [COVID-19]) vaccine, mRNA-LNP, spike protein, preservative free, 30 mcg/0.3mL dosage, diluent reconstituted; first dose	0 (0)	0 (0)		0 (0)	0 (0)	

© 2023 Olaker VR et al. *JAMA Network Open.*

Immunization administration by intramuscular injection of severe acute respiratory syndrome coronavirus 2 (SARS-CoV-2) (Coronavirus disease [COVID-19]) vaccine, mRNA-LNP, spike protein, preservative free, 30 mcg/0.3mL dosage, diluent reconstituted; second dose	0 (0)	0 (0)		0 (0)	0 (0)	
Severe acute respiratory syndrome coronavirus 2 (SARS-CoV-2) (Coronavirus disease [COVID-19]) vaccine, mRNA-LNP, spike protein, preservative free, 100 mcg/0.5mL dosage, for intramuscular use	0 (0)	0 (0)		0 (0)	0 (0)	
Immunization administration by intramuscular injection of severe acute respiratory syndrome coronavirus 2 (SARS-CoV-2) (Coronavirus disease [COVID-19]) vaccine, mRNA-LNP, spike protein, preservative free, 100 mcg/0.5mL dosage; first dose	0 (0)	0 (0)		0 (0)	0 (0)	
Immunization administration by intramuscular injection of severe acute respiratory syndrome coronavirus 2 (SARS-CoV-2) (Coronavirus disease [COVID-19]) vaccine, mRNA-LNP, spike protein, preservative free, 100 mcg/0.5mL dosage; second dose	0 (0)	0 (0)		0 (0)	0 (0)	
Severe acute respiratory syndrome coronavirus 2 (SARS-CoV-2) (coronavirus disease [COVID-19]) vaccine, DNA, spike protein, chimpanzee adenovirus Oxford 1 (ChAdOx1) vector, preservative free, 5x10^10 viral particles/0.5mL dosage, for intramuscular use	0 (0)	0 (0)		0 (0)	0 (0)	
Immunization administration by intramuscular injection of severe acute respiratory syndrome coronavirus 2 (SARS-CoV-2) (coronavirus disease [COVID-19]) vaccine, DNA, spike protein, chimpanzee adenovirus Oxford 1 (ChAdOx1) vector, preservative free, 5x10^10 viral particles/0.5mL dosage; first dose	0 (0)	0 (0)		0 (0)	0 (0)	

© 2023 Olaker VR et al. *JAMA Network Open.*

Immunization administration by intramuscular injection of severe acute respiratory syndrome coronavirus 2 (SARS-CoV-2) (coronavirus disease [COVID-19]) vaccine, DNA, spike protein, chimpanzee adenovirus Oxford 1 (ChAdOx1) vector, preservative free, 5x10^10 viral particles/0.5mL dosage; second dose	0 (0)	0 (0)		0 (0)	0 (0)	
Severe acute respiratory syndrome coronavirus 2 (SARS-CoV-2) (coronavirus disease [COVID-19]) vaccine, DNA, spike protein, adenovirus type 26 (Ad26) vector, preservative free, 5x10^10 viral particles/0.5mL dosage, for intramuscular use	0 (0)	0 (0)		0 (0)	0 (0)	
Immunization administration by intramuscular injection of severe acute respiratory syndrome coronavirus 2 (SARS-CoV-2) (coronavirus disease [COVID-19]) vaccine, DNA, spike protein, adenovirus type 26 (Ad26) vector, preservative free, 5x10^10 viral particles/0.5mL dosage, single dose	0 (0)	0 (0)		0 (0)	0 (0)	
Introduction of COVID-19 Vaccine Dose 1 into Subcutaneous Tissue, Percutaneous Approach, New Technology Group 6	0 (0)	0 (0)		0 (0)	0 (0)	
Introduction of COVID-19 Vaccine Dose 2 into Subcutaneous Tissue, Percutaneous Approach, New Technology Group 6	0 (0)	0 (0)		0 (0)	0 (0)	
Introduction of COVID-19 Vaccine into Subcutaneous Tissue, Percutaneous Approach, New Technology Group 6	0 (0)	0 (0)		0 (0)	0 (0)	
Introduction of COVID-19 Vaccine into Muscle, Percutaneous Approach, New Technology Group 6	0 (0)	0 (0)		0 (0)	0 (0)	
Introduction of COVID-19 Vaccine Dose 2 into Muscle, Percutaneous Approach, New Technology Group 6	0 (0)	0 (0)		0 (0)	0 (0)	
Introduction of COVID-19 Vaccine Dose 1 into Muscle, Percutaneous Approach, New Technology Group 6	0 (0)	0 (0)		0 (0)	0 (0)	
Visit: Inpatient Encounter	20742 (25.9)	35025 (28.2)	0.05	18378 (25.6)	17817 (24.8)	[illegible]
Visit: Short Stay	2172 (2.7)	2297 (1.8)	0.06	1692 (2.4)	1679 (2.3)	[illegible]
Visit: Inpatient Non-acute	137 (0.2)	419 (0.3)	0.03	129 (0.2)	126 (0.2)	[illegible]

© 2023 Olaker VR et al. *JAMA Network Open.*

SARS-CoV-2 (COVID-19) Vaccine	10 (0)	0 (0)	0.02	10 (0)	0 (0)	[illegible]

© 2023 Olaker VR et al. *JAMA Network Open.*

eTable 3. Baseline Characteristics for Block 3 Before and After Matching

	Before matching			After matching		
	Cohort, no, (%)			Cohort, no, (%)		
Characteristic Name	COVID-19	ORI	SMD	COVID-19	ORI	SMD
Total number	86933	153891		83803	83803	
Current Age, mean (SD), y	46.3 (19.1)	46.5 (20.3)	0.01	46.4 (19.1)	46.4 (19.9)	0.00
Age at Index, mean (SD), y	44.4 (19.2)	44.3 (20.3)	0.01	44.2 (19.1)	44.1 (19.9)	0.00
Gender						
Female	49556 (57)	93506 (60.8)	0.08	48108 (57.4)	47843 (57.1)	0.02
Male	36535 (42)	56402 (36.7)	0.11	34853 (41.6)	34985 (41.7)	0.01
Unknown	842 (1)	3983 (2.6)	0.12	842 (1)	975 (1.2)	0.00
Ethnicity						
Hispanic or Latino	12250 (14.1)	12008 (7.8)	0.20	10423 (12.4)	10465 (12.5)	0.02
Not Hispanic or Latino	58869 (67.7)	110076 (71.5)	0.08	57605 (68.7)	56863 (67.9)	0.00
Unknown	15814 (18.2)	31807 (20.7)	0.06	15775 (18.8)	16475 (19.7)	0.02
Race						
Asian	1962 (2.3)	2897 (1.9)	0.03	1922 (2.3)	1943 (2.3)	0.00
Black or African American	13022 (15)	20976 (13.6)	0.04	12737 (15.2)	12541 (15)	0.01
Native American or Alaska Native	277 (0.3)	468 (0.3)	0.00	269 (0.3)	265 (0.3)	0.00
Native Hawaiian or Other Pacific Islander	242 (0.3)	232 (0.2)	0.03	198 (0.2)	212 (0.3)	0.00
Unknown	14756 (17)	20023 (13)	0.11	13565 (16.2)	14180 (16.9)	0.02
White	56674 (65.2)	109295 (71)	0.13	55112 (65.8)	54662 (65.2)	0.01
Hypertensive diseases	23646 (27.2)	45684 (29.7)	0.06	22409 (26.7)	21596 (25.8)	0.02
Neoplasms	20096 (23.1)	37969 (24.7)	0.04	18998 (22.7)	18597 (22.2)	0.01
Other anxiety disorders	16621 (19.1)	37349 (24.3)	0.13	15873 (18.9)	15498 (18.5)	0.01
Overweight and obesity	15314 (17.6)	28039 (18.2)	0.02	14468 (17.3)	13992 (16.7)	0.02
Other forms of heart disease	15145 (17.4)	28696 (18.6)	0.03	14093 (16.8)	13650 (16.3)	0.01
Depressive episode	13516 (15.5)	28587 (18.6)	0.08	12787 (15.3)	12434 (14.8)	0.01
Type 2 diabetes mellitus	12753 (14.7)	19974 (13)	0.05	11691 (14)	11370 (13.6)	0.01
Asthma	9726 (11.2)	24343 (15.8)	0.14	9491 (11.3)	9158 (10.9)	0.01
Nicotine dependence	8889 (10.2)	19577 (12.7)	0.08	8498 (10.1)	8387 (10)	0.00
Generalized anxiety disorder	5389 (6.2)	11301 (7.3)	0.05	5063 (6)	4829 (5.8)	0.01

© 2023 Olaker VR et al. *JAMA Network Open.*

Persons with potential health hazards related to socioeconomic and psychosocial circumstances	5002 (5.8)	6911 (4.5)	0.06	4401 (5.3)	4293 (5.1)	0.01
Reaction to severe stress, and adjustment disorders	4505 (5.2)	9672 (6.3)	0.05	4277 (5.1)	4156 (5)	0.01
Bronchitis, not specified as acute or chronic	4425 (5.1)	11495 (7.5)	0.10	4267 (5.1)	4141 (4.9)	0.01
Major depressive disorder, recurrent	3709 (4.3)	7769 (5)	0.04	3463 (4.1)	3366 (4)	0.01
Other chronic obstructive pulmonary disease	3436 (4)	11430 (7.4)	0.15	3333 (4)	3300 (3.9)	0.00
Cerebral infarction	3669 (4.2)	5102 (3.3)	0.05	3218 (3.8)	3176 (3.8)	0.00
Fatty (change of) liver, not elsewhere classified	2999 (3.5)	5262 (3.4)	0.00	2787 (3.3)	2660 (3.2)	0.01
Phobic anxiety disorders	2971 (3.4)	2960 (1.9)	0.09	2377 (2.8)	2269 (2.7)	0.01
Attention-deficit hyperactivity disorders	2425 (2.8)	5442 (3.5)	0.04	2356 (2.8)	2252 (2.7)	0.01
Other psychoactive substance related disorders	2644 (3)	3988 (2.6)	0.03	2320 (2.8)	2331 (2.8)	0.00
Hypertensive chronic kidney disease	2189 (2.5)	4586 (3)	0.03	2052 (2.4)	1980 (2.4)	0.01
Certain disorders involving the immune mechanism	2006 (2.3)	4807 (3.1)	0.05	1925 (2.3)	1935 (2.3)	0.00
Bipolar disorder	1728 (2)	3812 (2.5)	0.03	1626 (1.9)	1533 (1.8)	0.01
Other specified diseases of liver	1685 (1.9)	3852 (2.5)	0.04	1620 (1.9)	1591 (1.9)	0.00
Schizophrenia, schizotypal, delusional, and other non-mood psychotic disorders	1820 (2.1)	2562 (1.7)	0.03	1572 (1.9)	1478 (1.8)	0.01
Type 1 diabetes mellitus	1626 (1.9)	2876 (1.9)	0.00	1510 (1.8)	1414 (1.7)	0.01
Other rheumatoid arthritis	1450 (1.7)	3234 (2.1)	0.03	1378 (1.6)	1329 (1.6)	0.00
Personal history of other mental and behavioral disorders	1525 (1.8)	2239 (1.5)	0.02	1354 (1.6)	1335 (1.6)	0.00
Emphysema	1333 (1.5)	4854 (3.2)	0.11	1299 (1.6)	1332 (1.6)	0.00
Cannabis related disorders	1233 (1.4)	2499 (1.6)	0.02	1167 (1.4)	1163 (1.4)	0.00
Psoriasis	1180 (1.4)	2448 (1.6)	0.02	1127 (1.3)	1076 (1.3)	0.01
Malignant neoplasms of lymphoid, hematopoietic and related tissue	1041 (1.2)	2558 (1.7)	0.04	1001 (1.2)	995 (1.2)	0.00
Other stimulant related disorders	1126 (1.3)	1223 (0.8)	0.05	948 (1.1)	934 (1.1)	0.00
Opioid related disorders	880 (1)	2551 (1.7)	0.06	842 (1)	876 (1)	0.00
Fibrosis and cirrhosis of liver	900 (1)	1622 (1.1)	0.00	825 (1)	812 (1)	0.00
Unspecified dementia	798 (0.9)	1249 (0.8)	0.01	715 (0.9)	713 (0.9)	0.00
Systemic lupus erythematosus (SLE)	555 (0.6)	1229 (0.8)	0.02	532 (0.6)	536 (0.6)	0.00
Rheumatoid arthritis with rheumatoid factor	519 (0.6)	959 (0.6)	0.00	484 (0.6)	483 (0.6)	0.00
Bronchiectasis	463 (0.5)	2040 (1.3)	0.08	458 (0.5)	493 (0.6)	0.01
Unspecified chronic bronchitis	457 (0.5)	1639 (1.1)	0.06	443 (0.5)	452 (0.5)	0.00
Simple and mucopurulent chronic bronchitis	390 (0.4)	1447 (0.9)	0.06	380 (0.5)	399 (0.5)	0.00
Chronic passive congestion of liver	372 (0.4)	999 (0.6)	0.03	360 (0.4)	350 (0.4)	0.00
Dementia in other diseases classified elsewhere	363 (0.4)	567 (0.4)	0.01	326 (0.4)	304 (0.4)	0.00

© 2023 Olaker VR et al. *JAMA Network Open.*

Cocaine related disorders	347 (0.4)	880 (0.6)	0.02	320 (0.4)	344 (0.4)	0.00
Hepatic failure, not elsewhere classified	306 (0.4)	665 (0.4)	0.01	286 (0.3)	286 (0.3)	0.00
Alzheimer's disease	318 (0.4)	441 (0.3)	0.01	282 (0.3)	262 (0.3)	0.00
Inhalant related disorders	299 (0.3)	699 (0.5)	0.02	280 (0.3)	286 (0.3)	0.00
Portal hypertension	274 (0.3)	514 (0.3)	0.00	257 (0.3)	266 (0.3)	0.00
Borderline personality disorder	270 (0.3)	531 (0.3)	0.01	251 (0.3)	244 (0.3)	0.00
Family history of other psychoactive substance abuse and dependence	268 (0.3)	249 (0.2)	0.03	205 (0.2)	205 (0.2)	0.00
Vascular dementia	216 (0.2)	348 (0.2)	0.00	189 (0.2)	180 (0.2)	0.00
Sedative, hypnotic, or anxiolytic related disorders	185 (0.2)	535 (0.3)	0.03	167 (0.2)	175 (0.2)	0.00
Hallucinogen related disorders	174 (0.2)	328 (0.2)	0.00	159 (0.2)	158 (0.2)	0.00
Chronic hepatitis, not elsewhere classified	106 (0.1)	174 (0.1)	0.00	95 (0.1)	95 (0.1)	0.00
Dementia with Lewy bodies	36 (0)	61 (0)	0.00	34 (0)	28 (0)	0.00
Frontotemporal dementia	31 (0)	43 (0)	0.00	29 (0)	28 (0)	0.00
Antisocial personality disorder	30 (0)	45 (0)	0.00	25 (0)	25 (0)	0.00
Blood Pressure, Systolic	47528 (54.7)	88351 (57.4)	0.07	45652 (54.5)	44172 (52.7)	0.00
Blood Pressure, Systolic, At least 160 mm[Hg]	14708 (16.9)	24232 (15.7)	0.03	13525 (16.1)	12972 (15.5)	0.02
Blood Pressure, Systolic, 140-160 mm[Hg]	27033 (31.1)	47060 (30.6)	0.01	25444 (30.4)	24581 (29.3)	0.02
Blood Pressure, Systolic, At most 140 mm[Hg]	45732 (52.6)	85920 (55.8)	0.06	43942 (52.4)	42503 (50.7)	0.03
Blood Pressure, Diastolic	47122 (54.2)	87693 (57)	0.01	45247 (54)	43817 (52.3)	0.04
Blood Pressure, Diastolic, At least 100 mm[Hg]	10560 (12.1)	17093 (11.1)	0.03	9634 (11.5)	9325 (11.1)	0.01
Blood Pressure, Diastolic, 90-100 mm[Hg]	20834 (24)	36573 (23.8)	0.00	19508 (23.3)	18822 (22.5)	0.02
Blood Pressure, Diastolic, At most 90 mm[Hg]	46317 (53.3)	86440 (56.2)	0.06	44474 (53.1)	43023 (51.3)	0.03
BMI	35894 (41.3)	66711 (43.4)	0.10	34574 (41.3)	32974 (39.3)	0.02
BMI, At least 30 kg/m2	17580 (20.2)	30894 (20.1)	0.00	16712 (19.9)	16100 (19.2)	0.02
BMI, 25-30 kg/m2	15887 (18.3)	31215 (20.3)	0.05	15397 (18.4)	15000 (17.9)	0.01
BMI, At most 25 kg/m2	14537 (16.7)	32294 (21)	0.11	14275 (17)	14030 (16.7)	0.01
Hospital Inpatient Services	9837 (11.3)	18017 (11.7)	0.01	9004 (10.7)	8558 (10.2)	0.02
New or Established Patient Initial Hospital Inpatient Care Services	8191 (9.4)	14782 (9.6)	0.01	7430 (8.9)	7044 (8.4)	0.02
Critical Care Services	2488 (2.9)	5135 (3.3)	0.03	2279 (2.7)	2218 (2.6)	0.00
Renal Transplantation Procedures	117 (0.1)	148 (0.1)	0.01	108 (0.1)	108 (0.1)	0.00
Liver Transplantation Procedures	16 (0)	31 (0)	0.00	16 (0)	13 (0)	0.00

© 2023 Olaker VR et al. *JAMA Network Open.*

Severe acute respiratory syndrome coronavirus 2 (SARS-CoV-2) (Coronavirus disease [COVID-19]) vaccine, mRNA-LNP, spike protein, preservative free, 30 mcg/0.3mL dosage, diluent reconstituted, for intramuscular use	0 (0)	0 (0)		0 (0)	0 (0)	
Immunization administration by intramuscular injection of severe acute respiratory syndrome coronavirus 2 (SARS-CoV-2) (Coronavirus disease [COVID-19]) vaccine, mRNA-LNP, spike protein, preservative free, 30 mcg/0.3mL dosage, diluent reconstituted; first dose	0 (0)	0 (0)		0 (0)	0 (0)	
Immunization administration by intramuscular injection of severe acute respiratory syndrome coronavirus 2 (SARS-CoV-2) (Coronavirus disease [COVID-19]) vaccine, mRNA-LNP, spike protein, preservative free, 30 mcg/0.3mL dosage, diluent reconstituted; second dose	0 (0)	0 (0)		0 (0)	0 (0)	
Severe acute respiratory syndrome coronavirus 2 (SARS-CoV-2) (Coronavirus disease [COVID-19]) vaccine, mRNA-LNP, spike protein, preservative free, 100 mcg/0.5mL dosage, for intramuscular use	0 (0)	0 (0)		0 (0)	0 (0)	
Immunization administration by intramuscular injection of severe acute respiratory syndrome coronavirus 2 (SARS-CoV-2) (Coronavirus disease [COVID-19]) vaccine, mRNA-LNP, spike protein, preservative free, 100 mcg/0.5mL dosage; first dose	0 (0)	0 (0)		0 (0)	0 (0)	
Immunization administration by intramuscular injection of severe acute respiratory syndrome coronavirus 2 (SARS-CoV-2) (Coronavirus disease [COVID-19]) vaccine, mRNA-LNP, spike protein, preservative free, 100 mcg/0.5mL dosage; second dose	0 (0)	0 (0)		0 (0)	0 (0)	
Severe acute respiratory syndrome coronavirus 2 (SARS-CoV-2) (coronavirus disease [COVID-19]) vaccine, DNA, spike protein, chimpanzee adenovirus Oxford 1 (ChAdOx1) vector, preservative free, 5x10^10 viral particles/0.5mL dosage, for intramuscular use	0 (0)	0 (0)		0 (0)	0 (0)	

© 2023 Olaker VR et al. *JAMA Network Open.*

Immunization administration by intramuscular injection of severe acute respiratory syndrome coronavirus 2 (SARS-CoV-2) (coronavirus disease [COVID-19]) vaccine, DNA, spike protein, chimpanzee adenovirus Oxford 1 (ChAdOx1) vector, preservative free, 5x10^10 viral particles/0.5mL dosage; first dose	0 (0)	0 (0)		0 (0)	0 (0)	
Immunization administration by intramuscular injection of severe acute respiratory syndrome coronavirus 2 (SARS-CoV-2) (coronavirus disease [COVID-19]) vaccine, DNA, spike protein, chimpanzee adenovirus Oxford 1 (ChAdOx1) vector, preservative free, 5x10^10 viral particles/0.5mL dosage; second dose	0 (0)	0 (0)		0 (0)	0 (0)	
Severe acute respiratory syndrome coronavirus 2 (SARS-CoV-2) (coronavirus disease [COVID-19]) vaccine, DNA, spike protein, adenovirus type 26 (Ad26) vector, preservative free, 5x10^10 viral particles/0.5mL dosage, for intramuscular use	0 (0)	0 (0)		0 (0)	0 (0)	
Immunization administration by intramuscular injection of severe acute respiratory syndrome coronavirus 2 (SARS-CoV-2) (coronavirus disease [COVID-19]) vaccine, DNA, spike protein, adenovirus type 26 (Ad26) vector, preservative free, 5x10^10 viral particles/0.5mL dosage, single dose	0 (0)	0 (0)		0 (0)	0 (0)	
Introduction of COVID-19 Vaccine Dose 1 into Subcutaneous Tissue, Percutaneous Approach, New Technology Group 6	0 (0)	0 (0)		0 (0)	0 (0)	
Introduction of COVID-19 Vaccine Dose 2 into Subcutaneous Tissue, Percutaneous Approach, New Technology Group 6	0 (0)	0 (0)		0 (0)	0 (0)	
Introduction of COVID-19 Vaccine into Subcutaneous Tissue, Percutaneous Approach, New Technology Group 6	0 (0)	0 (0)		0 (0)	0 (0)	
Introduction of COVID-19 Vaccine into Muscle, Percutaneous Approach, New Technology Group 6	0 (0)	0 (0)		0 (0)	0 (0)	
Introduction of COVID-19 Vaccine Dose 2 into Muscle, Percutaneous Approach, New Technology Group 6	0 (0)	0 (0)		0 (0)	0 (0)	

© 2023 Olaker VR et al. *JAMA Network Open.*

Introduction of COVID-19 Vaccine Dose 1 into Muscle, Percutaneous Approach, New Technology Group 6	0 (0)	0 (0)		0 (0)	0 (0)	
Visit: Inpatient Encounter	21527 (24.8)	41631 (27.1)	0.05	20406 (24.4)	19453 (23.2)	0.03
Visit: Short Stay	1566 (1.8)	2349 (1.5)	0.02	1434 (1.7)	1410 (1.7)	0.00
Visit: Inpatient Non-acute	1803 (2.1)	826 (0.5)	0.14	1023 (1.2)	806 (1)	0.02
SARS-CoV-2 (COVID-19) Vaccine	0 (0)	10 (0)	0.01	0 (0)	0 (0)	

© 2023 Olaker VR et al. *JAMA Network Open.*

eTable 4. Baseline Characteristics for Block 4 Before and After Matching

	Before matching			After matching		
	Cohort, No. (%)			Cohort, No. (%)		
Characteristic Name	COVID-19	ORI	SMD	COVID-19	ORI	SMD
Total number	250424	167118		159182	159182	
Current Age, mean (SD), y	49.0 (19.0)	47.0 (20.2)	0.11	46.6 (19.2)	47.1 (20.1)	0.02
Age at Index, mean (SD), y	47.0 (19.0)	45.0 (20.2)	0.11	44.6 (19.2)		0.02
Gender						
Female	139916 (55.9)	101977 (61)	0.10	96920 (60.9)	96138 (60.4)	0.01
Male	105664 (42.2)	59874 (35.8)	0.13	57751 (36.3)	58568 (36.8)	0.01
Unknown	4844 (1.9)	5267 (3.2)	0.08	4511 (2.8)	4476 (2.8)	0.00
Ethnicity						
Hispanic or Latino	30753 (12.3)	12217 (7.3)	0.17	12385 (7.8)	12174 (7.6)	0.00
Not Hispanic or Latino	169515 (67.7)	119070 (71.2)	0.08	113759 (71.5)	113176 (71.1)	0.01
Unknown	50156 (20)	35831 (21.4)	0.03	33038 (20.8)	33832 (21.3)	0.01
Race						
Asian	5999 (2.4)	3002 (1.8)	0.04	2961 (1.9)	2980 (1.9)	0.00
Black or African American	40999 (16.4)	23055 (13.8)	0.07	22911 (14.4)	22616 (14.2)	0.01
Native American or Alaska Native	766 (0.3)	481 (0.3)	0.00	466 (0.3)	451 (0.3)	0.00
Native Hawaiian or Other Pacific Islander	369 (0.1)	168 (0.1)	0.01	187 (0.1)	167 (0.1)	0.00
Unknown	35324 (14.1)	18788 (11.2)	0.09	18042 (11.3)	18414 (11.6)	0.01
White	166967 (66.7)	121624 (72.8)	0.13	114615 (72)	114554 (72)	0.00
Hypertensive diseases	77044 (30.8)	51199 (30.6)	0.00	48067 (30.2)	48137 (30.2)	0.00
Neoplasms	58637 (23.4)	41977 (25.1)	0.04	39573 (24.9)	39072 (24.5)	0.01
Other anxiety disorders	48832 (19.5)	42958 (25.7)	0.15	38293 (24.1)	38239 (24)	0.00
Overweight and obesity	52168 (20.8)	32566 (19.5)	0.03	31305 (19.7)	31075 (19.5)	0.00
Other forms of heart disease	45317 (18.1)	30918 (18.5)	0.01	28894 (18.2)	28754 (18.1)	0.00
Depressive episode	38848 (15.5)	31604 (18.9)	0.09	28526 (17.9)	28462 (17.9)	0.00
Asthma	29871 (11.9)	27122 (16.2)	0.12	23863 (15)	23868 (15)	0.00
Type 2 diabetes mellitus	39009 (15.6)	22055 (13.2)	0.07	21007 (13.2)	21086 (13.2)	0.00
Nicotine dependence	21889 (8.7)	21219 (12.7)	0.13	17802 (11.2)	18082 (11.4)	0.01
Generalized anxiety disorder	14727 (5.9)	13087 (7.8)	0.08	11581 (7.3)	11611 (7.3)	0.00
Bronchitis, not specified as acute or chronic	13709 (5.5)	12900 (7.7)	0.09	10989 (6.9)	11126 (7)	0.00
Reaction to severe stress, and adjustment disorders	13663 (5.5)	11103 (6.6)	0.05	10007 (6.3)	9973 (6.3)	0.00
Other chronic obstructive pulmonary disease	10953 (4.4)	11670 (7)	0.11	9092 (5.7)	9431 (5.9)	0.01
Major depressive disorder, recurrent	10438 (4.2)	8675 (5.2)	0.05	7729 (4.9)	7703 (4.8)	0.00

© 2023 Olaker VR et al. *JAMA Network Open.*

Persons with potential health hazards related to socioeconomic and psychosocial circumstances	11345 (4.5)	7224 (4.3)	0.01	6944 (4.4)	6851 (4.3)	0.00
Fatty (change of) liver, not elsewhere classified	9728 (3.9)	6043 (3.6)	0.01	5682 (3.6)	5694 (3.6)	0.00
Attention-deficit hyperactivity disorders	6660 (2.7)	6128 (3.7)	0.06	5470 (3.4)	5488 (3.4)	0.00
Cerebral infarction	8707 (3.5)	5260 (3.1)	0.02	5107 (3.2)	4968 (3.1)	0.00
Certain disorders involving the immune mechanism	6541 (2.6)	5259 (3.1)	0.03	4635 (2.9)	4658 (2.9)	0.00
Hypertensive chronic kidney disease	6984 (2.8)	4650 (2.8)	0.00	4297 (2.7)	4278 (2.7)	0.00
Other specified diseases of liver	5737 (2.3)	4237 (2.5)	0.02	3771 (2.4)	3850 (2.4)	0.00
Other psychoactive substance related disorders	5811 (2.3)	4059 (2.4)	0.01	3719 (2.3)	3683 (2.3)	0.00
Emphysema	4237 (1.7)	4882 (2.9)	0.08	3608 (2.3)	3788 (2.4)	0.01
Phobic anxiety disorders	6053 (2.4)	3505 (2.1)	0.02	3557 (2.2)	3378 (2.1)	0.01
Bipolar disorder	4511 (1.8)	4150 (2.5)	0.05	3527 (2.2)	3592 (2.3)	0.00
Other rheumatoid arthritis	4403 (1.8)	3423 (2)	0.02	3130 (2)	3071 (1.9)	0.00
Type 1 diabetes mellitus	5087 (2)	3156 (1.9)	0.01	2940 (1.8)	2950 (1.9)	0.00
Psoriasis	3495 (1.4)	2859 (1.7)	0.03	2583 (1.6)	2597 (1.6)	0.00
Schizophrenia, schizotypal, delusional, and other non-mood psychotic disorders	4099 (1.6)	2549 (1.5)	0.01	2438 (1.5)	2416 (1.5)	0.00
Cannabis related disorders	3078 (1.2)	2765 (1.7)	0.04	2400 (1.5)	2424 (1.5)	0.00
Malignant neoplasms of lymphoid, hematopoietic and related tissue	3027 (1.2)	2715 (1.6)	0.04	2354 (1.5)	2386 (1.5)	0.00
Opioid related disorders	2372 (0.9)	2812 (1.7)	0.06	2079 (1.3)	2189 (1.4)	0.01
Personal history of other mental and behavioral disorders	3305 (1.3)	2144 (1.3)	0.00	2072 (1.3)	2024 (1.3)	0.00
Fibrosis and cirrhosis of liver	2534 (1)	1597 (1)	0.01	1515 (1)	1477 (0.9)	0.00
Other stimulant related disorders	1931 (0.8)	1297 (0.8)	0.00	1215 (0.8)	1225 (0.8)	0.00
Bronchiectasis	1299 (0.5)	2052 (1.2)	0.08	1212 (0.8)	1443 (0.9)	0.02
Systemic lupus erythematosus (SLE)	1573 (0.6)	1307 (0.8)	0.02	1177 (0.7)	1168 (0.7)	0.00
Unspecified chronic bronchitis	1306 (0.5)	1752 (1)	0.06	1169 (0.7)	1282 (0.8)	0.01
Unspecified dementia	2475 (1)	1196 (0.7)	0.03	1152 (0.7)	1165 (0.7)	0.00
Simple and mucopurulent chronic bronchitis	1082 (0.4)	1595 (1)	0.06	982 (0.6)	1091 (0.7)	0.01
Rheumatoid arthritis with rheumatoid factor	1372 (0.5)	1010 (0.6)	0.01	962 (0.6)	910 (0.6)	0.00
Chronic passive congestion of liver	1187 (0.5)	1048 (0.6)	0.02	890 (0.6)	897 (0.6)	0.00
Cocaine related disorders	901 (0.4)	874 (0.5)	0.02	692 (0.4)	740 (0.5)	0.00
Dementia in other diseases classified elsewhere	1270 (0.5)	625 (0.4)	0.02	634 (0.4)	608 (0.4)	0.00
Hepatic failure, not elsewhere classified	881 (0.4)	654 (0.4)	0.01	595 (0.4)	578 (0.4)	0.00
Inhalant related disorders	734 (0.3)	663 (0.4)	0.02	550 (0.3)	563 (0.4)	0.00
Alzheimer's disease	1083 (0.4)	506 (0.3)	0.02	522 (0.3)	492 (0.3)	0.00
Borderline personality disorder	643 (0.3)	576 (0.3)	0.02	507 (0.3)	507 (0.3)	0.00

© 2023 Olaker VR et al. *JAMA Network Open.*

Portal hypertension	786 (0.3)	488 (0.3)	0.00	455 (0.3)	448 (0.3)	0.00
Sedative, hypnotic, or anxiolytic related disorders	535 (0.2)	594 (0.4)	0.03	440 (0.3)	487 (0.3)	0.01
Vascular dementia	685 (0.3)	352 (0.2)	0.01	339 (0.2)	333 (0.2)	0.00
Family history of other psychoactive substance abuse and dependence	450 (0.2)	252 (0.2)	0.01	264 (0.2)	248 (0.2)	0.00
Hallucinogen related disorders	336 (0.1)	306 (0.2)	0.01	256 (0.2)	274 (0.2)	0.00
Chronic hepatitis, not elsewhere classified	288 (0.1)	199 (0.1)	0.00	182 (0.1)	186 (0.1)	0.00
Dementia with Lewy bodies	108 (0)	74 (0)	0.00	69 (0)	67 (0)	0.00
Frontotemporal dementia	72 (0)	53 (0)	0.00	46 (0)	48 (0)	0.00
Antisocial personality disorder	63 (0)	38 (0)	0.00	41 (0)	34 (0)	0.00
Blood Pressure, Systolic	135523 (54.1)	98170 (58.7)	0.07	92973 (58.4)	92303 (58)	0.02
Blood Pressure, Systolic, At least 160 mm[Hg]	42552 (17)	26879 (16.1)	0.02	25526 (16)	25455 (16)	0.00
Blood Pressure, Systolic, 140-160 mm[Hg]	79144 (31.6)	52519 (31.4)	0.00	50048 (31.4)	49800 (31.3)	0.00
Blood Pressure, Systolic, At most 140 mm[Hg]	130327 (52)	95367 (57.1)	0.10	90304 (56.7)	89528 (56.2)	0.01
Blood Pressure, Diastolic	134594 (53.7)	97800 (58.5)	0.02	92463 (58.1)	91940 (57.8)	0.03
Blood Pressure, Diastolic, At least 100 mm[Hg]	30724 (12.3)	19019 (11.4)	0.03	18060 (11.3)	18083 (11.4)	0.00
Blood Pressure, Diastolic, 90-100 mm[Hg]	61948 (24.7)	40973 (24.5)	0.01	38894 (24.4)	38766 (24.4)	0.00
Blood Pressure, Diastolic, At most 90 mm[Hg]	132210 (52.8)	96416 (57.7)	0.10	91375 (57.4)	90565 (56.9)	0.01
BMI	106277 (42.4)	71285 (42.7)	0.16	68751 (43.2)	67273 (42.3)	0.03
BMI, At least 30 kg/m2	56887 (22.7)	34136 (20.4)	0.06	32990 (20.7)	32924 (20.7)	0.00
BMI, 25-30 kg/m2	49378 (19.7)	33510 (20.1)	0.01	31687 (19.9)	31738 (19.9)	0.00
BMI, At most 25 kg/m2	40963 (16.4)	33828 (20.2)	0.10	30977 (19.5)	30859 (19.4)	0.00
Hospital Inpatient Services	26905 (10.7)	19104 (11.4)	0.02	17379 (10.9)	17465 (11)	0.00
New or Established Patient Initial Hospital Inpatient Care Services	21881 (8.7)	15533 (9.3)	0.02	14092 (8.9)	14168 (8.9)	0.00
Critical Care Services	7019 (2.8)	5385 (3.2)	0.02	4752 (3)	4780 (3)	0.00
Renal Transplantation Procedures	347 (0.1)	144 (0.1)	0.02	161 (0.1)	143 (0.1)	0.00
Severe acute respiratory syndrome coronavirus 2 (SARS-CoV-2) (Coronavirus disease [COVID-19]) vaccine, mRNA-LNP, spike protein, preservative free, 30 mcg/0.3mL dosage, diluent reconstituted, for intramuscular use	275 (0.1)	112 (0.1)	0.01	108 (0.1)	109 (0.1)	0.00
Immunization administration by intramuscular injection of severe acute respiratory syndrome coronavirus 2 (SARS-CoV-2) (Coronavirus disease [COVID-19]) vaccine, mRNA-LNP, spike protein, preservative free, 30 mcg/0.3mL dosage, diluent reconstituted; first dose	272 (0.1)	111 (0.1)	0.01	107 (0.1)	108 (0.1)	0.00

© 2023 Olaker VR et al. *JAMA Network Open.*

Severe acute respiratory syndrome coronavirus 2 (SARS-CoV-2) (Coronavirus disease [COVID-19]) vaccine, mRNA-LNP, spike protein, preservative free, 100 mcg/0.5mL dosage, for intramuscular use	116 (0)	75 (0)	0.00	76 (0)	73 (0)	0.00
Immunization administration by intramuscular injection of severe acute respiratory syndrome coronavirus 2 (SARS-CoV-2) (Coronavirus disease [COVID-19]) vaccine, mRNA-LNP, spike protein, preservative free, 100 mcg/0.5mL dosage; first dose	114 (0)	72 (0)	0.00	74 (0)	70 (0)	0.00
Liver Transplantation Procedures	56 (0)	44 (0)	0.00	39 (0)	41 (0)	0.00
Immunization administration by intramuscular injection of severe acute respiratory syndrome coronavirus 2 (SARS-CoV-2) (Coronavirus disease [COVID-19]) vaccine, mRNA-LNP, spike protein, preservative free, 30 mcg/0.3mL dosage, diluent reconstituted; second dose	27 (0)	34 (0)	0.01	19 (0)	31 (0)	0.01
Immunization administration by intramuscular injection of severe acute respiratory syndrome coronavirus 2 (SARS-CoV-2) (Coronavirus disease [COVID-19]) vaccine, mRNA-LNP, spike protein, preservative free, 100 mcg/0.5mL dosage; second dose	10 (0)	0 (0)	0.01	0 (0)	0 (0)	
Severe acute respiratory syndrome coronavirus 2 (SARS-CoV-2) (coronavirus disease [COVID-19]) vaccine, DNA, spike protein, chimpanzee adenovirus Oxford 1 (ChAdOx1) vector, preservative free, 5x10^10 viral particles/0.5mL dosage, for intramuscular use	0 (0)	0 (0)		0 (0)	0 (0)	
Immunization administration by intramuscular injection of severe acute respiratory syndrome coronavirus 2 (SARS-CoV-2) (coronavirus disease [COVID-19]) vaccine, DNA, spike protein, chimpanzee adenovirus Oxford 1 (ChAdOx1) vector, preservative free, 5x10^10 viral particles/0.5mL dosage; first dose	0 (0)	0 (0)		0 (0)	0 (0)	

© 2023 Olaker VR et al. *JAMA Network Open.*

Immunization administration by intramuscular injection of severe acute respiratory syndrome coronavirus 2 (SARS-CoV-2) (coronavirus disease [COVID-19]) vaccine, DNA, spike protein, chimpanzee adenovirus Oxford 1 (ChAdOx1) vector, preservative free, 5x10^10 viral particles/0.5mL dosage; second dose	0 (0)	0 (0)		0 (0)	0 (0)	
Severe acute respiratory syndrome coronavirus 2 (SARS-CoV-2) (coronavirus disease [COVID-19]) vaccine, DNA, spike protein, adenovirus type 26 (Ad26) vector, preservative free, 5x10^10 viral particles/0.5mL dosage, for intramuscular use	0 (0)	0 (0)		0 (0)	0 (0)	
Immunization administration by intramuscular injection of severe acute respiratory syndrome coronavirus 2 (SARS-CoV-2) (coronavirus disease [COVID-19]) vaccine, DNA, spike protein, adenovirus type 26 (Ad26) vector, preservative free, 5x10^10 viral particles/0.5mL dosage, single dose	0 (0)	0 (0)		0 (0)	0 (0)	
Introduction of COVID-19 Vaccine Dose 1 into Subcutaneous Tissue, Percutaneous Approach, New Technology Group 6	0 (0)	0 (0)		0 (0)	0 (0)	
Introduction of COVID-19 Vaccine Dose 2 into Subcutaneous Tissue, Percutaneous Approach, New Technology Group 6	0 (0)	0 (0)		0 (0)	0 (0)	
Introduction of COVID-19 Vaccine into Subcutaneous Tissue, Percutaneous Approach, New Technology Group 6	0 (0)	0 (0)		0 (0)	0 (0)	
Introduction of COVID-19 Vaccine into Muscle, Percutaneous Approach, New Technology Group 6	0 (0)	0 (0)		0 (0)	0 (0)	
Introduction of COVID-19 Vaccine Dose 2 into Muscle, Percutaneous Approach, New Technology Group 6	0 (0)	0 (0)		0 (0)	0 (0)	
Introduction of COVID-19 Vaccine Dose 1 into Muscle, Percutaneous Approach, New Technology Group 6	0 (0)	0 (0)		0 (0)	0 (0)	
Visit: Inpatient Encounter	62270 (24.9)	45478 (27.2)	0.05	41591 (26.1)	41904 (26.3)	0.00
Visit: Short Stay	6336 (2.5)	2575 (1.5)	0.07	2589 (1.6)	2540 (1.6)	0.00
Visit: Inpatient Non-acute	2953 (1.2)	931 (0.6)	0.07	985 (0.6)	931 (0.6)	0.00
SARS-CoV-2 (COVID-19) Vaccine	446 (0.2)	161 (0.1)	0.02	150 (0.1)	157 (0.1)	0.00

© 2023 Olaker VR et al. *JAMA Network Open.*

eTable 5. Baseline Characteristics for Block 5 Before and After Matching

	Before matching			After matching		
	Cohort, No. (%)			Cohort, No. (%)		
Characteristic Name	COVID-19	ORI	SMD	COVID-19	ORI	SMD
Total number	133249	141426		113402	113402	
Current Age, mean (SD), y	49.3 (18.9)	47.6 (21.0)	0.09	48.0 (19.0)	48.3 (20.6)	0.01
Age at Index, mean (SD), y	47.6 (18.9)	45.8 (21.0)	0.09	46.3 (19.0)	46.6 (20.6)	0.01
Gender						
Female	75006 (56.3)	85177 (60.2)	0.08	66227 (58.4)	65789 (58)	0.01
Male	55942 (42)	52317 (37)	0.10	44879 (39.6)	45147 (39.8)	0.00
Unknown	2301 (1.7)	3932 (2.8)	0.07	2296 (2)	2466 (2.2)	0.01
Ethnicity						
Hispanic or Latino	13387 (10)	10646 (7.5)	0.09	9575 (8.4)	9826 (8.7)	0.00
Not Hispanic or Latino	95592 (71.7)	101924 (72.1)	0.01	81841 (72.2)	81632 (72)	0.01
Unknown	24270 (18.2)	28856 (20.4)	0.06	21986 (19.4)	21944 (19.4)	0.00
Race						
Asian	3476 (2.6)	2632 (1.9)	0.05	2438 (2.2)	2483 (2.2)	0.00
Black or African American	23465 (17.6)	21437 (15.2)	0.07	18695 (16.5)	18929 (16.7)	0.00
Native American or Alaska Native	353 (0.3)	414 (0.3)	0.01	322 (0.3)	312 (0.3)	0.01
Native Hawaiian or Other Pacific Islander	192 (0.1)	139 (0.1)	0.01	123 (0.1)	130 (0.1)	0.00
Unknown	17560 (13.2)	16293 (11.5)	0.05	14021 (12.4)	14231 (12.5)	0.01
White	88203 (66.2)	100511 (71.1)	0.11	77803 (68.6)	77317 (68.2)	0.01
Hypertensive diseases	44208 (33.2)	47013 (33.2)	0.00	36531 (32.2)	36509 (32.2)	0.00
Neoplasms	35387 (26.6)	38806 (27.4)	0.02	29580 (26.1)	29606 (26.1)	0.00
Other anxiety disorders	28891 (21.7)	36672 (25.9)	0.10	25659 (22.6)	25294 (22.3)	0.01
Overweight and obesity	29744 (22.3)	28843 (20.4)	0.05	23658 (20.9)	23679 (20.9)	0.00
Other forms of heart disease	27346 (20.5)	29864 (21.1)	0.01	22506 (19.8)	22333 (19.7)	0.00
Depressive episode	23279 (17.5)	27788 (19.6)	0.06	20004 (17.6)	19864 (17.5)	0.00
Type 2 diabetes mellitus	23015 (17.3)	20844 (14.7)	0.07	17276 (15.2)	17366 (15.3)	0.00
Asthma	17614 (13.2)	24906 (17.6)	0.12	16259 (14.3)	16154 (14.2)	0.00
Nicotine dependence	14978 (11.2)	18890 (13.4)	0.06	13124 (11.6)	13147 (11.6)	0.00
Generalized anxiety disorder	8845 (6.6)	11140 (7.9)	0.05	7780 (6.9)	7615 (6.7)	0.01
Bronchitis, not specified as acute or chronic	7626 (5.7)	11359 (8)	0.09	6981 (6.2)	6962 (6.1)	0.00
Reaction to severe stress, and adjustment disorders	7964 (6)	9826 (6.9)	0.04	6929 (6.1)	6863 (6.1)	0.00
Other chronic obstructive pulmonary disease	6585 (4.9)	11851 (8.4)	0.14	6193 (5.5)	6229 (5.5)	0.00
Persons with potential health hazards related to socioeconomic and psychosocial circumstances	7850 (5.9)	6922 (4.9)	0.04	5878 (5.2)	5744 (5.1)	0.01
Major depressive disorder, recurrent	5993 (4.5)	7484 (5.3)	0.04	5176 (4.6)	5135 (4.5)	0.00

© 2023 Olaker VR et al. *JAMA Network Open.*

Cerebral infarction	6034 (4.5)	5359 (3.8)	0.04	4429 (3.9)	4344 (3.8)	0.00
Fatty (change of) liver, not elsewhere classified	5334 (4)	5538 (3.9)	0.00	4270 (3.8)	4269 (3.8)	0.00
Attention-deficit hyperactivity disorders	3944 (3)	5360 (3.8)	0.05	3621 (3.2)	3584 (3.2)	0.00
Certain disorders involving the immune mechanism	4171 (3.1)	5211 (3.7)	0.03	3541 (3.1)	3560 (3.1)	0.00
Phobic anxiety disorders	5399 (4.1)	3248 (2.3)	0.10	3308 (2.9)	3038 (2.7)	0.01
Hypertensive chronic kidney disease	4110 (3.1)	4673 (3.3)	0.01	3274 (2.9)	3297 (2.9)	0.00
Other psychoactive substance related disorders	4207 (3.2)	3780 (2.7)	0.03	3090 (2.7)	3021 (2.7)	0.00
Other specified diseases of liver	3147 (2.4)	3931 (2.8)	0.03	2692 (2.4)	2650 (2.3)	0.00
Bipolar disorder	2905 (2.2)	3655 (2.6)	0.03	2528 (2.2)	2484 (2.2)	0.00
Emphysema	2623 (2)	5057 (3.6)	0.10	2463 (2.2)	2545 (2.2)	0.00
Other rheumatoid arthritis	2837 (2.1)	3293 (2.3)	0.01	2402 (2.1)	2359 (2.1)	0.00
Type 1 diabetes mellitus	2806 (2.1)	2909 (2.1)	0.00	2241 (2)	2213 (2)	0.00
Schizophrenia, schizotypal, delusional, and other non-mood psychotic disorders	2688 (2)	2379 (1.7)	0.02	1998 (1.8)	1946 (1.7)	0.00
Malignant neoplasms of lymphoid, hematopoietic and related tissue	2221 (1.7)	2879 (2)	0.03	1968 (1.7)	1974 (1.7)	0.00
Cannabis related disorders	2238 (1.7)	2453 (1.7)	0.00	1861 (1.6)	1882 (1.7)	0.00
Psoriasis	2142 (1.6)	2508 (1.8)	0.01	1795 (1.6)	1828 (1.6)	0.00
Personal history of other mental and behavioral disorders	2238 (1.7)	2070 (1.5)	0.02	1715 (1.5)	1703 (1.5)	0.00
Opioid related disorders	1613 (1.2)	2602 (1.8)	0.05	1469 (1.3)	1448 (1.3)	0.00
Fibrosis and cirrhosis of liver	1677 (1.3)	1588 (1.1)	0.01	1270 (1.1)	1247 (1.1)	0.00
Other stimulant related disorders	1584 (1.2)	1197 (0.8)	0.03	1068 (0.9)	1055 (0.9)	0.00
Unspecified dementia	1140 (0.9)	1188 (0.8)	0.00	880 (0.8)	878 (0.8)	0.00
Systemic lupus erythematosus (SLE)	1043 (0.8)	1172 (0.8)	0.01	858 (0.8)	869 (0.8)	0.00
Bronchiectasis	845 (0.6)	2313 (1.6)	0.09	824 (0.7)	871 (0.8)	0.00
Unspecified chronic bronchitis	850 (0.6)	1690 (1.2)	0.06	791 (0.7)	816 (0.7)	0.00
Rheumatoid arthritis with rheumatoid factor	889 (0.7)	951 (0.7)	0.00	725 (0.6)	706 (0.6)	0.00
Chronic passive congestion of liver	684 (0.5)	972 (0.7)	0.02	600 (0.5)	599 (0.5)	0.00
Simple and mucopurulent chronic bronchitis	626 (0.5)	1563 (1.1)	0.07	599 (0.5)	655 (0.6)	0.01
Cocaine related disorders	601 (0.5)	844 (0.6)	0.02	540 (0.5)	543 (0.5)	0.00
Hepatic failure, not elsewhere classified	612 (0.5)	672 (0.5)	0.00	488 (0.4)	493 (0.4)	0.00
Dementia in other diseases classified elsewhere	466 (0.4)	619 (0.4)	0.01	401 (0.4)	404 (0.4)	0.00
Portal hypertension	523 (0.4)	484 (0.3)	0.01	396 (0.3)	380 (0.3)	0.00
Inhalant related disorders	431 (0.3)	614 (0.4)	0.02	395 (0.3)	378 (0.3)	0.00
Borderline personality disorder	430 (0.3)	470 (0.3)	0.00	359 (0.3)	343 (0.3)	0.00
Alzheimer's disease	420 (0.3)	489 (0.3)	0.01	344 (0.3)	353 (0.3)	0.00
Sedative, hypnotic, or anxiolytic related disorders	355 (0.3)	508 (0.4)	0.02	313 (0.3)	303 (0.3)	0.00
Vascular dementia	287 (0.2)	352 (0.2)	0.01	238 (0.2)	250 (0.2)	0.00

© 2023 Olaker VR et al. *JAMA Network Open.*

Family history of other psychoactive substance abuse and dependence	359 (0.3)	247 (0.2)	0.02	222 (0.2)	225 (0.2)	0.00
Hallucinogen related disorders	241 (0.2)	301 (0.2)	0.01	212 (0.2)	215 (0.2)	0.00
Chronic hepatitis, not elsewhere classified	197 (0.1)	218 (0.2)	0.00	165 (0.1)	162 (0.1)	0.00
Dementia with Lewy bodies	59 (0)	57 (0)	0.00	41 (0)	43 (0)	0.00
Antisocial personality disorder	51 (0)	32 (0)	0.01	33 (0)	30 (0)	0.00
Frontotemporal dementia	31 (0)	45 (0)	0.01	27 (0)	29 (0)	0.00
Blood Pressure, Systolic	77412 (58.1)	83243 (58.9)	0.03	65609 (57.9)	64801 (57.1)	0.04
Blood Pressure, Systolic, At least 160 mm[Hg]	26596 (20)	24624 (17.4)	0.07	20427 (18)	20370 (18)	0.00
Blood Pressure, Systolic, 140-160 mm[Hg]	47438 (35.6)	45556 (32.2)	0.07	37778 (33.3)	37801 (33.3)	0.00
Blood Pressure, Systolic, At most 140 mm[Hg]	74517 (55.9)	80695 (57.1)	0.02	63169 (55.7)	62606 (55.2)	0.01
Blood Pressure, Diastolic	75826 (56.9)	82648 (58.4)	0.02	64559 (56.9)	64229 (56.6)	0.05
Blood Pressure, Diastolic, At least 100 mm[Hg]	18643 (14)	16771 (11.9)	0.06	14154 (12.5)	14115 (12.4)	0.00
Blood Pressure, Diastolic, 90-100 mm[Hg]	36932 (27.7)	35376 (25)	0.06	29258 (25.8)	29262 (25.8)	0.00
Blood Pressure, Diastolic, At most 90 mm[Hg]	74498 (55.9)	81419 (57.6)	0.03	63656 (56.1)	63033 (55.6)	0.01
BMI	61179 (45.9)	61600 (43.6)	0.15	50650 (44.7)	49082 (43.3)	0.02
BMI, At least 30 kg/m2	32454 (24.4)	29458 (20.8)	0.08	25239 (22.3)	25153 (22.2)	0.00
BMI, 25-30 kg/m2	27365 (20.5)	28590 (20.2)	0.01	22750 (20.1)	22681 (20)	0.00
BMI, At most 25 kg/m2	23960 (18)	29413 (20.8)	0.07	21492 (19)	21322 (18.8)	0.00
Hospital Inpatient Services	16628 (12.5)	18864 (13.3)	0.03	13761 (12.1)	13643 (12)	0.00
New or Established Patient Initial Hospital Inpatient Care Services	13662 (10.3)	15527 (11)	0.02	11232 (9.9)	11135 (9.8)	0.00
Critical Care Services	4365 (3.3)	5620 (4)	0.04	3722 (3.3)	3715 (3.3)	0.00
Severe acute respiratory syndrome coronavirus 2 (SARS-CoV-2) (Coronavirus disease [COVID-19]) vaccine, mRNA-LNP, spike protein, preservative free, 30 mcg/0.3mL dosage, diluent reconstituted, for intramuscular use	1648 (1.2)	3670 (2.6)	0.10	1578 (1.4)	1693 (1.5)	0.01
Immunization administration by intramuscular injection of severe acute respiratory syndrome coronavirus 2 (SARS-CoV-2) (Coronavirus disease [COVID-19]) vaccine, mRNA-LNP, spike protein, preservative free, 30 mcg/0.3mL dosage, diluent reconstituted; first dose	1634 (1.2)	3621 (2.6)	0.10	1564 (1.4)	1675 (1.5)	0.01
Severe acute respiratory syndrome coronavirus 2 (SARS-CoV-2) (Coronavirus disease [COVID-19]) vaccine, mRNA-LNP, spike protein, preservative free, 100 mcg/0.5mL dosage, for intramuscular use	776 (0.6)	1012 (0.7)	0.02	608 (0.5)	584 (0.5)	0.00

© 2023 Olaker VR et al. *JAMA Network Open.*

Immunization administration by intramuscular injection of severe acute respiratory syndrome coronavirus 2 (SARS-CoV-2) (Coronavirus disease [COVID-19]) vaccine, mRNA-LNP, spike protein, preservative free, 100 mcg/0.5mL dosage; first dose	771 (0.6)	998 (0.7)	0.02	603 (0.5)	578 (0.5)	0.00
Immunization administration by intramuscular injection of severe acute respiratory syndrome coronavirus 2 (SARS-CoV-2) (Coronavirus disease [COVID-19]) vaccine, mRNA-LNP, spike protein, preservative free, 30 mcg/0.3mL dosage, diluent reconstituted; second dose	558 (0.4)	2164 (1.5)	0.11	558 (0.5)	660 (0.6)	0.01
Immunization administration by intramuscular injection of severe acute respiratory syndrome coronavirus 2 (SARS-CoV-2) (Coronavirus disease [COVID-19]) vaccine, mRNA-LNP, spike protein, preservative free, 100 mcg/0.5mL dosage; second dose	193 (0.1)	580 (0.4)	0.05	192 (0.2)	201 (0.2)	0.00
Renal Transplantation Procedures	230 (0.2)	137 (0.1)	0.02	118 (0.1)	123 (0.1)	0.00
Liver Transplantation Procedures	24 (0)	35 (0)	0.00	24 (0)	21 (0)	0.00
Severe acute respiratory syndrome coronavirus 2 (SARS-CoV-2) (coronavirus disease [COVID-19]) vaccine, DNA, spike protein, adenovirus type 26 (Ad26) vector, preservative free, 5x10^10 viral particles/0.5mL dosage, for intramuscular use	20 (0)	49 (0)	0.01	20 (0)	21 (0)	0.00
Immunization administration by intramuscular injection of severe acute respiratory syndrome coronavirus 2 (SARS-CoV-2) (coronavirus disease [COVID-19]) vaccine, DNA, spike protein, adenovirus type 26 (Ad26) vector, preservative free, 5x10^10 viral particles/0.5mL dosage, single dose	20 (0)	49 (0)	0.01	20 (0)	21 (0)	0.00
Introduction of COVID-19 Vaccine Dose 2 into Subcutaneous Tissue, Percutaneous Approach, New Technology Group 6	10 (0)	0 (0)	0.01	10 (0)	0 (0)	0.01
Introduction of COVID-19 Vaccine into Muscle, Percutaneous Approach, New Technology Group 6	10 (0)	10 (0)	0.00	10 (0)	10 (0)	0.00
Introduction of COVID-19 Vaccine Dose 1 into Muscle, Percutaneous Approach, New Technology Group 6	10 (0)	10 (0)	0.00	10 (0)	10 (0)	0.00

© 2023 Olaker VR et al. *JAMA Network Open.*

Severe acute respiratory syndrome coronavirus 2 (SARS-CoV-2) (coronavirus disease [COVID-19]) vaccine, DNA, spike protein, chimpanzee adenovirus Oxford 1 (ChAdOx1) vector, preservative free, 5x10^10 viral particles/0.5mL dosage, for intramuscular use	0 (0)	0 (0)		0 (0)	0 (0)	
Immunization administration by intramuscular injection of severe acute respiratory syndrome coronavirus 2 (SARS-CoV-2) (coronavirus disease [COVID-19]) vaccine, DNA, spike protein, chimpanzee adenovirus Oxford 1 (ChAdOx1) vector, preservative free, 5x10^10 viral particles/0.5mL dosage; first dose	0 (0)	0 (0)		0 (0)	0 (0)	
Immunization administration by intramuscular injection of severe acute respiratory syndrome coronavirus 2 (SARS-CoV-2) (coronavirus disease [COVID-19]) vaccine, DNA, spike protein, chimpanzee adenovirus Oxford 1 (ChAdOx1) vector, preservative free, 5x10^10 viral particles/0.5mL dosage; second dose	0 (0)	0 (0)		0 (0)	0 (0)	
Introduction of COVID-19 Vaccine Dose 1 into Subcutaneous Tissue, Percutaneous Approach, New Technology Group 6	0 (0)	0 (0)		0 (0)	0 (0)	
Introduction of COVID-19 Vaccine into Subcutaneous Tissue, Percutaneous Approach, New Technology Group 6	0 (0)	0 (0)		0 (0)	0 (0)	
Introduction of COVID-19 Vaccine Dose 2 into Muscle, Percutaneous Approach, New Technology Group 6	10 (0)	10 (0)	0.00	0 (0)	0 (0)	
Visit: Inpatient Encounter	36501 (27.4)	40873 (28.9)	0.03	30835 (27.2)	30590 (27)	0.00
Visit: Short Stay	3126 (2.3)	2219 (1.6)	0.06	2045 (1.8)	2031 (1.8)	0.00
Visit: Inpatient Non-acute	2468 (1.9)	920 (0.7)	0.11	1225 (1.1)	915 (0.8)	0.03
SARS-CoV-2 (COVID-19) Vaccine	2725 (2)	3561 (2.5)	0.03	2197 (1.9)	2247 (2)	0.00

© 2023 Olaker VR et al. *JAMA Network Open.*

eTable 6. Baseline Characteristics for Block 6 Before and After Matching

	Before matching			After matching		
	Cohort, No. (%)			Cohort, No. (%)		
Characteristic Name	COVID-19	ORI	SMD	COVID-19	ORI	SMD
Total number	88388	170100		82302	82302	
Current Age, mean (SD), y	48.5 (19.4)	47.1 (21.2)	0.07	48.0 (19.3)	48.7 (20.5)	0.04
Age at Index, mean (SD), y	47.0 (19.4)	45.6 (21.2)	0.07	46.5 (19.3)	47.2 (20.5)	0.04
Gender						
Female	51433 (58.2)	102691 (60.4)	0.04	47753 (58)	47436 (57.6)	0.01
Male	36263 (41)	63309 (37.2)	0.08	33857 (41.1)	34125 (41.5)	0.01
Unknown	692 (0.8)	4100 (2.4)	0.13	692 (0.8)	741 (0.9)	0.01
Ethnicity						
Hispanic or Latino	8135 (9.2)	13609 (8)	0.04	7739 (9.4)	7749 (9.4)	0.00
Not Hispanic or Latino	69041 (78.1)	123068 (72.4)	0.13	63409 (77)	62745 (76.2)	0.02
Unknown	11212 (12.7)	33423 (19.6)	0.19	11154 (13.6)	11808 (14.3)	0.02
Race						
Asian	1985 (2.2)	3333 (2)	0.02	1866 (2.3)	1908 (2.3)	0.00
Black or African American	13981 (15.8)	25056 (14.7)	0.03	13239 (16.1)	13595 (16.5)	0.01
Native American or Alaska Native	211 (0.2)	510 (0.3)	0.01	202 (0.2)	195 (0.2)	0.00
Native Hawaiian or Other Pacific Islander	130 (0.1)	175 (0.1)	0.01	119 (0.1)	116 (0.1)	0.00
Unknown	10903 (12.3)	19817 (11.7)	0.02	10381 (12.6)	10823 (13.2)	0.02
White	61178 (69.2)	121209 (71.3)	0.04	56495 (68.6)	55665 (67.6)	0.02
Hypertensive diseases	31421 (35.5)	55307 (32.5)	0.06	27485 (33.4)	26985 (32.8)	0.01
Neoplasms	29927 (33.9)	45899 (27)	0.15	25187 (30.6)	24623 (29.9)	0.01
Other anxiety disorders	24048 (27.2)	43396 (25.5)	0.04	20499 (24.9)	19648 (23.9)	0.02
Other forms of heart disease	23295 (26.4)	35460 (20.8)	0.13	19307 (23.5)	18921 (23)	0.01
Overweight and obesity	21011 (23.8)	34153 (20.1)	0.09	18042 (21.9)	17624 (21.4)	0.01
Depressive episode	20288 (23)	32351 (19)	0.10	16800 (20.4)	16369 (19.9)	0.01
Type 2 diabetes mellitus	17909 (20.3)	24609 (14.5)	0.15	14665 (17.8)	14436 (17.5)	0.01
Nicotine dependence	14667 (16.6)	22229 (13.1)	0.10	12217 (14.8)	11835 (14.4)	0.01
Asthma	13341 (15.1)	28534 (16.8)	0.05	11975 (14.6)	11463 (13.9)	0.02
Persons with potential health hazards related to socioeconomic and psychosocial circumstances	9248 (10.5)	8763 (5.2)	0.20	6528 (7.9)	6414 (7.8)	0.01
Generalized anxiety disorder	7543 (8.5)	12991 (7.6)	0.03	6240 (7.6)	6007 (7.3)	0.01
Reaction to severe stress, and adjustment disorders	6615 (7.5)	11376 (6.7)	0.03	5581 (6.8)	5426 (6.6)	0.01
Bronchitis, not specified as acute or chronic	5991 (6.8)	13394 (7.9)	0.04	5308 (6.4)	5092 (6.2)	0.01
Cerebral infarction	7075 (8)	6582 (3.9)	0.18	5009 (6.1)	4828 (5.9)	0.01
Other chronic obstructive pulmonary disease	5603 (6.3)	13283 (7.8)	0.06	4922 (6)	4712 (5.7)	0.01
Phobic anxiety disorders	7447 (8.4)	4595 (2.7)	0.25	4336 (5.3)	4112 (5)	0.01
Major depressive disorder, recurrent	4799 (5.4)	8568 (5)	0.02	3947 (4.8)	3872 (4.7)	0.00

© 2023 Olaker VR et al. *JAMA Network Open.*

Other psychoactive substance related disorders	4886 (5.5)	4531 (2.7)	0.14	3321 (4)	3176 (3.9)	0.01
Fatty (change of) liver, not elsewhere classified	3910 (4.4)	6485 (3.8)	0.03	3297 (4)	3183 (3.9)	0.01
Certain disorders involving the immune mechanism	3647 (4.1)	6062 (3.6)	0.03	3087 (3.8)	3058 (3.7)	0.00
Attention-deficit hyperactivity disorders	3325 (3.8)	6591 (3.9)	0.01	2923 (3.6)	2730 (3.3)	0.01
Hypertensive chronic kidney disease	2789 (3.2)	5272 (3.1)	0.00	2450 (3)	2366 (2.9)	0.01
Schizophrenia, schizotypal, delusional, and other non-mood psychotic disorders	3022 (3.4)	2875 (1.7)	0.11	2064 (2.5)	2010 (2.4)	0.00
Other rheumatoid arthritis	2596 (2.9)	3721 (2.2)	0.05	2058 (2.5)	2026 (2.5)	0.00
Emphysema	2277 (2.6)	5878 (3.5)	0.05	2009 (2.4)	1957 (2.4)	0.00
Bipolar disorder	2462 (2.8)	4121 (2.4)	0.02	2005 (2.4)	1930 (2.3)	0.01
Other specified diseases of liver	2208 (2.5)	4545 (2.7)	0.01	1931 (2.3)	1921 (2.3)	0.00
Personal history of other mental and behavioral disorders	2750 (3.1)	2641 (1.6)	0.10	1897 (2.3)	1823 (2.2)	0.01
Malignant neoplasms of lymphoid, hematopoietic and related tissue	2072 (2.3)	3301 (1.9)	0.03	1780 (2.2)	1767 (2.1)	0.00
Type 1 diabetes mellitus	2042 (2.3)	3389 (2)	0.02	1741 (2.1)	1698 (2.1)	0.00
Cannabis related disorders	2133 (2.4)	2928 (1.7)	0.05	1734 (2.1)	1689 (2.1)	0.00
Psoriasis	1758 (2)	2942 (1.7)	0.02	1476 (1.8)	1442 (1.8)	0.00
Other stimulant related disorders	2182 (2.5)	1420 (0.8)	0.13	1297 (1.6)	1242 (1.5)	0.01
Fibrosis and cirrhosis of liver	1604 (1.8)	1934 (1.1)	0.06	1233 (1.5)	1210 (1.5)	0.00
Opioid related disorders	1156 (1.3)	2705 (1.6)	0.02	1031 (1.3)	1027 (1.2)	0.00
Unspecified dementia	1056 (1.2)	1604 (0.9)	0.02	843 (1)	813 (1)	0.00
Bronchiectasis	803 (0.9)	2375 (1.4)	0.05	728 (0.9)	706 (0.9)	0.00
Systemic lupus erythematosus (SLE)	821 (0.9)	1363 (0.8)	0.01	694 (0.8)	700 (0.9)	0.00
Unspecified chronic bronchitis	785 (0.9)	1914 (1.1)	0.02	676 (0.8)	722 (0.9)	0.01
Rheumatoid arthritis with rheumatoid factor	772 (0.9)	1140 (0.7)	0.02	628 (0.8)	607 (0.7)	0.00
Simple and mucopurulent chronic bronchitis	565 (0.6)	1689 (1)	0.04	505 (0.6)	504 (0.6)	0.00
Hepatic failure, not elsewhere classified	476 (0.5)	815 (0.5)	0.01	401 (0.5)	402 (0.5)	0.00
Cocaine related disorders	433 (0.5)	901 (0.5)	0.01	393 (0.5)	414 (0.5)	0.00
Chronic passive congestion of liver	439 (0.5)	1103 (0.6)	0.02	392 (0.5)	399 (0.5)	0.00
Portal hypertension	470 (0.5)	616 (0.4)	0.03	374 (0.5)	363 (0.4)	0.00
Inhalant related disorders	414 (0.5)	713 (0.4)	0.01	352 (0.4)	356 (0.4)	0.00
Dementia in other diseases classified elsewhere	316 (0.4)	747 (0.4)	0.01	294 (0.4)	277 (0.3)	0.00
Family history of other psychoactive substance abuse and dependence	524 (0.6)	268 (0.2)	0.07	280 (0.3)	253 (0.3)	0.01
Borderline personality disorder	366 (0.4)	534 (0.3)	0.02	274 (0.3)	273 (0.3)	0.00
Alzheimer's disease	279 (0.3)	614 (0.4)	0.01	254 (0.3)	254 (0.3)	0.00
Sedative, hypnotic, or anxiolytic related disorders	267 (0.3)	575 (0.3)	0.01	225 (0.3)	229 (0.3)	0.00
Hallucinogen related disorders	252 (0.3)	378 (0.2)	0.01	196 (0.2)	199 (0.2)	0.00

© 2023 Olaker VR et al. *JAMA Network Open.*

Vascular dementia	211 (0.2)	445 (0.3)	0.00	186 (0.2)	194 (0.2)	0.00
Chronic hepatitis, not elsewhere classified	201 (0.2)	243 (0.1)	0.02	151 (0.2)	150 (0.2)	0.00
Dementia with Lewy bodies	42 (0)	85 (0.1)	0.00	38 (0)	40 (0)	0.00
Antisocial personality disorder	36 (0)	37 (0)	0.01	30 (0)	25 (0)	0.00
Frontotemporal dementia	20 (0)	57 (0)	0.01	17 (0)	19 (0)	0.00
Blood Pressure, Systolic	58085 (65.7)	103562 (60.9)	0.04	52093 (63.3)	50637 (61.5)	0.03
Blood Pressure, Systolic, At least 160 mm[Hg]	22553 (25.5)	31385 (18.5)	0.17	18640 (22.6)	18325 (22.3)	0.01
Blood Pressure, Systolic, 140-160 mm[Hg]	38046 (43)	57274 (33.7)	0.19	32631 (39.6)	32257 (39.2)	0.01
Blood Pressure, Systolic, At most 140 mm[Hg]	56363 (63.8)	100545 (59.1)	0.10	50394 (61.2)	49274 (59.9)	0.03
Blood Pressure, Diastolic	56777 (64.2)	102886 (60.5)	0.01	50979 (61.9)	50066 (60.8)	0.04
Blood Pressure, Diastolic, At least 100 mm[Hg]	16247 (18.4)	21298 (12.5)	0.16	13180 (16)	12931 (15.7)	0.01
Blood Pressure, Diastolic, 90-100 mm[Hg]	29942 (33.9)	44266 (26)	0.17	25353 (30.8)	24954 (30.3)	0.01
Blood Pressure, Diastolic, At most 90 mm[Hg]	56031 (63.4)	101485 (59.7)	0.08	50269 (61.1)	49166 (59.7)	0.03
BMI	49226 (55.7)	76775 (45.1)	0.15	43673 (53.1)	40488 (49.2)	0.00
BMI, At least 30 kg/m2	25903 (29.3)	36476 (21.4)	0.18	22152 (26.9)	22042 (26.8)	0.00
BMI, 25-30 kg/m2	21463 (24.3)	35693 (21)	0.08	19147 (23.3)	19049 (23.1)	0.00
BMI, At most 25 kg/m2	19407 (22)	36977 (21.7)	0.01	17803 (21.6)	17132 (20.8)	0.02
Hospital Inpatient Services	14421 (16.3)	22245 (13.1)	0.09	11962 (14.5)	11826 (14.4)	0.00
New or Established Patient Initial Hospital Inpatient Care Services	12232 (13.8)	18465 (10.9)	0.09	10024 (12.2)	9901 (12)	0.00
Critical Care Services	3841 (4.3)	6607 (3.9)	0.02	3204 (3.9)	3156 (3.8)	0.00
Severe acute respiratory syndrome coronavirus 2 (SARS-CoV-2) (Coronavirus disease [COVID-19]) vaccine, mRNA-LNP, spike protein, preservative free, 30 mcg/0.3mL dosage, diluent reconstituted, for intramuscular use	2805 (3.2)	9780 (5.8)	0.13	2734 (3.3)	2521 (3.1)	0.01
Immunization administration by intramuscular injection of severe acute respiratory syndrome coronavirus 2 (SARS-CoV-2) (Coronavirus disease [COVID-19]) vaccine, mRNA-LNP, spike protein, preservative free, 30 mcg/0.3mL dosage, diluent reconstituted; first dose	2748 (3.1)	9604 (5.6)	0.12	2678 (3.3)	2466 (3)	0.01
Immunization administration by intramuscular injection of severe acute respiratory syndrome coronavirus 2 (SARS-CoV-2) (Coronavirus disease [COVID-19]) vaccine, mRNA-LNP, spike protein, preservative free, 30	2274 (2.6)	8620 (5.1)	0.13	2228 (2.7)	2040 (2.5)	0.01

© 2023 Olaker VR et al. *JAMA Network Open.*

mcg/0.3mL dosage, diluent reconstituted; second dose						
Severe acute respiratory syndrome coronavirus 2 (SARS-CoV-2) (Coronavirus disease [COVID-19]) vaccine, mRNA-LNP, spike protein, preservative free, 100 mcg/0.5mL dosage, for intramuscular use	867 (1)	1931 (1.1)	0.02	786 (1)	769 (0.9)	0.00
Immunization administration by intramuscular injection of severe acute respiratory syndrome coronavirus 2 (SARS-CoV-2) (Coronavirus disease [COVID-19]) vaccine, mRNA-LNP, spike protein, preservative free, 100 mcg/0.5mL dosage; first dose	851 (1)	1886 (1.1)	0.01	774 (0.9)	747 (0.9)	0.00
Immunization administration by intramuscular injection of severe acute respiratory syndrome coronavirus 2 (SARS-CoV-2) (Coronavirus disease [COVID-19]) vaccine, mRNA-LNP, spike protein, preservative free, 100 mcg/0.5mL dosage; second dose	809 (0.9)	1773 (1)	0.01	728 (0.9)	695 (0.8)	0.00
Renal Transplantation Procedures	148 (0.2)	160 (0.1)	0.02	121 (0.1)	116 (0.1)	0.00
Severe acute respiratory syndrome coronavirus 2 (SARS-CoV-2) (coronavirus disease [COVID-19]) vaccine, DNA, spike protein, adenovirus type 26 (Ad26) vector, preservative free, 5x10^10 viral particles/0.5mL dosage, for intramuscular use	67 (0.1)	204 (0.1)	0.01	66 (0.1)	67 (0.1)	0.00
Immunization administration by intramuscular injection of severe acute respiratory syndrome coronavirus 2 (SARS-CoV-2) (coronavirus disease [COVID-19]) vaccine, DNA, spike protein, adenovirus type 26 (Ad26) vector, preservative free, 5x10^10 viral particles/0.5mL dosage, single dose	67 (0.1)	204 (0.1)	0.01	66 (0.1)	67 (0.1)	0.00
Liver Transplantation Procedures	21 (0)	40 (0)	0.00	17 (0)	21 (0)	0.00
Introduction of COVID-19 Vaccine into Muscle, Percutaneous Approach, New Technology Group 6	10 (0)	10 (0)	0.01	10 (0)	10 (0)	0.00
Introduction of COVID-19 Vaccine Dose 2 into Muscle, Percutaneous Approach, New Technology Group 6	10 (0)	10 (0)	0.01	10 (0)	10 (0)	0.00

© 2023 Olaker VR et al. *JAMA Network Open.*

Introduction of COVID-19 Vaccine Dose 1 into Muscle, Percutaneous Approach, New Technology Group 6	10 (0)	10 (0)	0.01	10 (0)	10 (0)	0.00
Severe acute respiratory syndrome coronavirus 2 (SARS-CoV-2) (coronavirus disease [COVID-19]) vaccine, DNA, spike protein, chimpanzee adenovirus Oxford 1 (ChAdOx1) vector, preservative free, 5x10^10 viral particles/0.5mL dosage, for intramuscular use	0 (0)	0 (0)		0 (0)	0 (0)	
Immunization administration by intramuscular injection of severe acute respiratory syndrome coronavirus 2 (SARS-CoV-2) (coronavirus disease [COVID-19]) vaccine, DNA, spike protein, chimpanzee adenovirus Oxford 1 (ChAdOx1) vector, preservative free, 5x10^10 viral particles/0.5mL dosage; first dose	0 (0)	0 (0)		0 (0)	0 (0)	
Immunization administration by intramuscular injection of severe acute respiratory syndrome coronavirus 2 (SARS-CoV-2) (coronavirus disease [COVID-19]) vaccine, DNA, spike protein, chimpanzee adenovirus Oxford 1 (ChAdOx1) vector, preservative free, 5x10^10 viral particles/0.5mL dosage; second dose	0 (0)	0 (0)		0 (0)	0 (0)	
Introduction of COVID-19 Vaccine Dose 1 into Subcutaneous Tissue, Percutaneous Approach, New Technology Group 6	0 (0)	0 (0)		0 (0)	0 (0)	
Introduction of COVID-19 Vaccine Dose 2 into Subcutaneous Tissue, Percutaneous Approach, New Technology Group 6	0 (0)	0 (0)		0 (0)	0 (0)	
Introduction of COVID-19 Vaccine into Subcutaneous Tissue, Percutaneous Approach, New Technology Group 6	0 (0)	0 (0)		0 (0)	0 (0)	
Visit: Inpatient Encounter	26644 (30.1)	47883 (28.2)	0.04	23548 (28.6)	23157 (28.1)	0.01
Visit: Inpatient Non-acute	3514 (4)	1631 (1)	0.20	1819 (2.2)	1598 (1.9)	0.02
Visit: Short Stay	1265 (1.4)	2763 (1.6)	0.02	1237 (1.5)	1281 (1.6)	0.00
SARS-CoV-2 (COVID-19) Vaccine	4681 (5.3)	10002 (5.9)	0.03	4207 (5.1)	4098 (5)	0.01

© 2023 Olaker VR et al. *JAMA Network Open.*

eTable 7. Baseline Characteristics for Block 7 Before and After Matching

	Before matching				After matching	
	Cohort, No. (%)				Cohort, NO. (%)	
Characteristic Name	COVID-19	ORI	SMD	COVID-19	ORI	SMD
Total number	185849	215303		162849	162849	
Current Age, mean (SD), y	47.5 (18.9)	43.9 (21.4)	0.18	46.1 (18.9)	47.0 (20.8)	0.05
Age at Index, mean (SD), y	46.3 (18.9)	42.7 (21.4)	0.18	44.8 (18.9)	45.7 (20.8)	0.05
Gender						
Female	106238 (57.2)	130058 (60.4)	0.07	95880 (58.9)	96416 (59.2)	0.01
Male	77193 (41.5)	79099 (36.7)	0.10	64551 (39.6)	63763 (39.2)	0.01
Unknown	2418 (1.3)	6146 (2.9)	0.11	2418 (1.5)	2670 (1.6)	0.01
Ethnicity						
Hispanic or Latino	19030 (10.2)	17675 (8.2)	0.07	15268 (9.4)	15582 (9.6)	0.01
Not Hispanic or Latino	139427 (75)	157896 (73.3)	0.04	121664 (74.7)	120829 (74.2)	0.01
Unknown	27392 (14.7)	39732 (18.5)	0.10	25917 (15.9)	26438 (16.2)	0.01
Race						
Asian	3496 (1.9)	4754 (2.2)	0.02	3305 (2)	3225 (2)	0.00
Black or African American	29006 (15.6)	28241 (13.1)	0.07	24042 (14.8)	24759 (15.2)	0.01
Native American or Alaska Native	651 (0.4)	655 (0.3)	0.01	520 (0.3)	549 (0.3)	0.00
Native Hawaiian or Other Pacific Islander	279 (0.2)	226 (0.1)	0.01	226 (0.1)	215 (0.1)	0.00
Unknown	22798 (12.3)	26158 (12.1)	0.00	20166 (12.4)	20325 (12.5)	0.00
White	129619 (69.7)	155269 (72.1)	0.05	114590 (70.4)	113776 (69.9)	0.01
Hypertensive diseases	55611 (29.9)	61216 (28.4)	0.03	47534 (29.2)	48737 (29.9)	0.02
Neoplasms	47084 (25.3)	53102 (24.7)	0.02	40217 (24.7)	41313 (25.4)	0.02
Other anxiety disorders	41563 (22.4)	54063 (25.1)	0.06	37149 (22.8)	37894 (23.3)	0.01
Overweight and obesity	39294 (21.1)	41806 (19.4)	0.04	33058 (20.3)	33637 (20.7)	0.01
Other forms of heart disease	36568 (19.7)	38632 (17.9)	0.04	30326 (18.6)	30841 (18.9)	0.01
Depressive episode	32297 (17.4)	39085 (18.2)	0.02	28036 (17.2)	28550 (17.5)	0.01
Asthma	23940 (12.9)	35987 (16.7)	0.11	22335 (13.7)	22974 (14.1)	0.01
Type 2 diabetes mellitus	27581 (14.8)	26160 (12.2)	0.08	21997 (13.5)	22267 (13.7)	0.00

© 2023 Olaker VR et al. *JAMA Network Open.*

Nicotine dependence	19916 (10.7)	23875 (11.1)	0.01	17387 (10.7)	17917 (11)	0.01
Generalized anxiety disorder	12757 (6.9)	16792 (7.8)	0.04	11411 (7)	11586 (7.1)	0.00
Reaction to severe stress, and adjustment disorders	11338 (6.1)	14471 (6.7)	0.03	9999 (6.1)	10264 (6.3)	0.01
Bronchitis, not specified as acute or chronic	10609 (5.7)	15392 (7.1)	0.06	9700 (6)	10135 (6.2)	0.01
Persons with potential health hazards related to socioeconomic and psychosocial circumstances	11020 (5.9)	10029 (4.7)	0.06	8462 (5.2)	8314 (5.1)	0.00
Other chronic obstructive pulmonary disease	8480 (4.6)	13301 (6.2)	0.07	7783 (4.8)	8156 (5)	0.01
Major depressive disorder, recurrent	8151 (4.4)	10542 (4.9)	0.02	7232 (4.4)	7407 (4.5)	0.01
Fatty (change of) liver, not elsewhere classified	7038 (3.8)	7817 (3.6)	0.01	5967 (3.7)	6103 (3.7)	0.00
Cerebral infarction	7729 (4.2)	6333 (2.9)	0.07	5671 (3.5)	5498 (3.4)	0.01
Phobic anxiety disorders	8483 (4.6)	5369 (2.5)	0.11	5623 (3.5)	5061 (3.1)	0.02
Attention-deficit hyperactivity disorders	5952 (3.2)	9174 (4.3)	0.06	5589 (3.4)	5672 (3.5)	0.00
Certain disorders involving the immune mechanism	5778 (3.1)	6497 (3)	0.01	4823 (3)	4986 (3.1)	0.01
Other psychoactive substance related disorders	5997 (3.2)	4814 (2.2)	0.06	4345 (2.7)	4145 (2.5)	0.01
Hypertensive chronic kidney disease	4962 (2.7)	5324 (2.5)	0.01	4093 (2.5)	4164 (2.6)	0.00
Other specified diseases of liver	4010 (2.2)	5280 (2.5)	0.02	3591 (2.2)	3734 (2.3)	0.01
Bipolar disorder	3831 (2.1)	4879 (2.3)	0.01	3344 (2.1)	3364 (2.1)	0.00
Other rheumatoid arthritis	3822 (2.1)	4239 (2)	0.01	3244 (2)	3319 (2)	0.00
Emphysema	3294 (1.8)	5739 (2.7)	0.06	3031 (1.9)	3233 (2)	0.01
Type 1 diabetes mellitus	3478 (1.9)	3602 (1.7)	0.02	2854 (1.8)	2858 (1.8)	0.00
Schizophrenia, schizotypal, delusional, and other non-mood psychotic disorders	3441 (1.9)	2993 (1.4)	0.04	2571 (1.6)	2529 (1.6)	0.00
Cannabis related disorders	3088 (1.7)	3286 (1.5)	0.01	2568 (1.6)	2633 (1.6)	0.00
Psoriasis	2938 (1.6)	3496 (1.6)	0.00	2551 (1.6)	2613 (1.6)	0.00
Personal history of other mental and behavioral disorders	3061 (1.6)	2895 (1.3)	0.02	2394 (1.5)	2366 (1.5)	0.00
Malignant neoplasms of lymphoid, hematopoietic and related tissue	2508 (1.3)	3337 (1.6)	0.02	2262 (1.4)	2375 (1.5)	0.01
Opioid related disorders	2211 (1.2)	2974 (1.4)	0.02	1963 (1.2)	2028 (1.2)	0.00
Fibrosis and cirrhosis of liver	2092 (1.1)	1948 (0.9)	0.02	1645 (1)	1618 (1)	0.00
Other stimulant related disorders	1898 (1)	1230 (0.6)	0.05	1221 (0.8)	1158 (0.7)	0.00
Unspecified dementia	1430 (0.8)	1472 (0.7)	0.01	1141 (0.7)	1166 (0.7)	0.00
Systemic lupus erythematosus (SLE)	1289 (0.7)	1504 (0.7)	0.00	1114 (0.7)	1155 (0.7)	0.00
Unspecified chronic bronchitis	1116 (0.6)	1966 (0.9)	0.04	1024 (0.6)	1092 (0.7)	0.01
Bronchiectasis	1022 (0.6)	2559 (1.2)	0.07	997 (0.6)	1159 (0.7)	0.01
Rheumatoid arthritis with rheumatoid factor	1184 (0.6)	1312 (0.6)	0.00	990 (0.6)	1039 (0.6)	0.00

© 2023 Olaker VR et al. *JAMA Network Open.*

Simple and mucopurulent chronic bronchitis	882 (0.5)	1771 (0.8)	0.04	826 (0.5)	869 (0.5)	0.00
Chronic passive congestion of liver	853 (0.5)	1218 (0.6)	0.01	775 (0.5)	828 (0.5)	0.00
Cocaine related disorders	809 (0.4)	935 (0.4)	0.00	665 (0.4)	696 (0.4)	0.00
Hepatic failure, not elsewhere classified	690 (0.4)	747 (0.3)	0.00	570 (0.4)	561 (0.3)	0.00
Inhalant related disorders	667 (0.4)	751 (0.3)	0.00	559 (0.3)	568 (0.3)	0.00
Dementia in other diseases classified elsewhere	650 (0.4)	716 (0.3)	0.00	540 (0.3)	565 (0.3)	0.00
Borderline personality disorder	565 (0.3)	626 (0.3)	0.00	475 (0.3)	458 (0.3)	0.00
Alzheimer's disease	546 (0.3)	567 (0.3)	0.01	445 (0.3)	466 (0.3)	0.00
Portal hypertension	566 (0.3)	522 (0.2)	0.01	431 (0.3)	415 (0.3)	0.00
Sedative, hypnotic, or anxiolytic related disorders	488 (0.3)	598 (0.3)	0.00	413 (0.3)	422 (0.3)	0.00
Family history of other psychoactive substance abuse and dependence	485 (0.3)	264 (0.1)	0.03	315 (0.2)	254 (0.2)	0.01
Vascular dementia	367 (0.2)	414 (0.2)	0.00	290 (0.2)	298 (0.2)	0.00
Hallucinogen related disorders	284 (0.2)	334 (0.2)	0.00	240 (0.1)	236 (0.1)	0.00
Chronic hepatitis, not elsewhere classified	211 (0.1)	213 (0.1)	0.00	182 (0.1)	163 (0.1)	0.00
Dementia with Lewy bodies	57 (0)	76 (0)	0.00	50 (0)	53 (0)	0.00
Frontotemporal dementia	44 (0)	51 (0)	0.00	41 (0)	41 (0)	0.00
Antisocial personality disorder	42 (0)	41 (0)	0.00	27 (0)	29 (0)	0.00
Blood Pressure, Systolic	107720 (58)	125182 (58.1)	0.13	93587 (57.5)	94155 (57.8)	0.01
Blood Pressure, Systolic, At least 160 mm[Hg]	36091 (19.4)	32919 (15.3)	0.11	28414 (17.4)	28515 (17.5)	0.00
Blood Pressure, Systolic, 140-160 mm[Hg]	65283 (35.1)	63790 (29.6)	0.12	53368 (32.8)	54275 (33.3)	0.01
Blood Pressure, Systolic, At most 140 mm[Hg]	103782 (55.8)	121940 (56.6)	0.02	90591 (55.6)	91103 (55.9)	0.01
Blood Pressure, Diastolic	107434 (57.8)	124975 (58)	0.04	93337 (57.3)	93990 (57.7)	0.04
Blood Pressure, Diastolic, At least 100 mm[Hg]	26013 (14)	23076 (10.7)	0.10	20240 (12.4)	20236 (12.4)	0.00
Blood Pressure, Diastolic, 90-100 mm[Hg]	51474 (27.7)	49670 (23.1)	0.11	41670 (25.6)	42250 (25.9)	0.01
Blood Pressure, Diastolic, At most 90 mm[Hg]	105631 (56.8)	123397 (57.3)	0.01	91996 (56.5)	92514 (56.8)	0.01
BMI	86152 (46.4)	95179 (44.2)	0.20	74051 (45.5)	72290 (44.4)	0.01
BMI, At least 30 kg/m2	45708 (24.6)	43647 (20.3)	0.10	37043 (22.7)	37607 (23.1)	0.01
BMI, 25-30 kg/m2	38834 (20.9)	43366 (20.1)	0.02	33630 (20.7)	34278 (21)	0.01
BMI, At most 25 kg/m2	33005 (17.8)	47695 (22.2)	0.11	30896 (19)	30567 (18.8)	0.01
Hospital Inpatient Services	23182 (12.5)	23713 (11)	0.05	18776 (11.5)	19077 (11.7)	0.01
New or Established Patient Initial Hospital Inpatient Care Services	18921 (10.2)	19232 (8.9)	0.04	15201 (9.3)	15435 (9.5)	0.00

© 2023 Olaker VR et al. *JAMA Network Open.*

Severe acute respiratory syndrome coronavirus 2 (SARS-CoV-2) (Coronavirus disease [COVID-19]) vaccine, mRNA-LNP, spike protein, preservative free, 30 mcg/0.3mL dosage, diluent reconstituted, for intramuscular use	8840 (4.8)	13419 (6.2)	0.06	8445 (5.2)	8627 (5.3)	0.01
Immunization administration by intramuscular injection of severe acute respiratory syndrome coronavirus 2 (SARS-CoV-2) (Coronavirus disease [COVID-19]) vaccine, mRNA-LNP, spike protein, preservative free, 30 mcg/0.3mL dosage, diluent reconstituted; first dose	8403 (4.5)	12871 (6)	0.07	8049 (4.9)	8239 (5.1)	0.01
Immunization administration by intramuscular injection of severe acute respiratory syndrome coronavirus 2 (SARS-CoV-2) (Coronavirus disease [COVID-19]) vaccine, mRNA-LNP, spike protein, preservative free, 30 mcg/0.3mL dosage, diluent reconstituted; second dose	7745 (4.2)	11820 (5.5)	0.06	7422 (4.6)	7601 (4.7)	0.01
Critical Care Services	6115 (3.3)	6563 (3)	0.01	4975 (3.1)	5052 (3.1)	0.00
Severe acute respiratory syndrome coronavirus 2 (SARS-CoV-2) (Coronavirus disease [COVID-19]) vaccine, mRNA-LNP, spike protein, preservative free, 100 mcg/0.5mL dosage, for intramuscular use	1490 (0.8)	2214 (1)	0.02	1400 (0.9)	1473 (0.9)	0.00
Immunization administration by intramuscular injection of severe acute respiratory syndrome coronavirus 2 (SARS-CoV-2) (Coronavirus disease [COVID-19]) vaccine, mRNA-LNP, spike protein, preservative free, 100 mcg/0.5mL dosage; first dose	1450 (0.8)	2133 (1)	0.02	1361 (0.8)	1437 (0.9)	0.01
Immunization administration by intramuscular injection of severe acute respiratory syndrome coronavirus 2 (SARS-CoV-2) (Coronavirus disease [COVID-19]) vaccine, mRNA-LNP, spike protein, preservative free, 100 mcg/0.5mL dosage; second dose	1364 (0.7)	2036 (0.9)	0.02	1280 (0.8)	1345 (0.8)	0.00
Renal Transplantation Procedures	372 (0.2)	201 (0.1)	0.03	226 (0.1)	192 (0.1)	0.01
Severe acute respiratory syndrome coronavirus 2 (SARS-CoV-2) (coronavirus disease [COVID-19]) vaccine, DNA, spike protein, adenovirus type 26 (Ad26) vector, preservative free, 5x10^10 viral	153 (0.1)	281 (0.1)	0.01	151 (0.1)	151 (0.1)	0.00

© 2023 Olaker VR et al. *JAMA Network Open.*

particles/0.5mL dosage, for intramuscular use						
Immunization administration by intramuscular injection of severe acute respiratory syndrome coronavirus 2 (SARS-CoV-2) (coronavirus disease [COVID-19]) vaccine, DNA, spike protein, adenovirus type 26 (Ad26) vector, preservative free, 5x10^10 viral particles/0.5mL dosage, single dose	152 (0.1)	280 (0.1)	0.01	150 (0.1)	150 (0.1)	0.00
Liver Transplantation Procedures	50 (0)	60 (0)	0.00	44 (0)	44 (0)	0.00
Introduction of COVID-19 Vaccine Dose 1 into Muscle, Percutaneous Approach, New Technology Group 6	26 (0)	15 (0)	0.01	18 (0)	15 (0)	0.00
Introduction of COVID-19 Vaccine Dose 1 into Subcutaneous Tissue, Percutaneous Approach, New Technology Group 6	10 (0)	10 (0)	0.00	10 (0)	10 (0)	0.00
Introduction of COVID-19 Vaccine into Muscle, Percutaneous Approach, New Technology Group 6	10 (0)	10 (0)	0.00	10 (0)	10 (0)	0.00
Introduction of COVID-19 Vaccine Dose 2 into Muscle, Percutaneous Approach, New Technology Group 6	10 (0)	10 (0)	0.00	10 (0)	10 (0)	0.00
Severe acute respiratory syndrome coronavirus 2 (SARS-CoV-2) (coronavirus disease [COVID-19]) vaccine, DNA, spike protein, chimpanzee adenovirus Oxford 1 (ChAdOx1) vector, preservative free, 5x10^10 viral particles/0.5mL dosage, for intramuscular use	0 (0)	0 (0)		0 (0)	0 (0)	
Immunization administration by intramuscular injection of severe acute respiratory syndrome coronavirus 2 (SARS-CoV-2) (coronavirus disease [COVID-19]) vaccine, DNA, spike protein, chimpanzee adenovirus Oxford 1 (ChAdOx1) vector, preservative free, 5x10^10 viral particles/0.5mL dosage; first dose	0 (0)	0 (0)		0 (0)	0 (0)	

© 2023 Olaker VR et al. *JAMA Network Open.*

Immunization administration by intramuscular injection of severe acute respiratory syndrome coronavirus 2 (SARS-CoV-2) (coronavirus disease [COVID-19]) vaccine, DNA, spike protein, chimpanzee adenovirus Oxford 1 (ChAdOx1) vector, preservative free, 5x10^10 viral particles/0.5mL dosage; second dose	0 (0)	0 (0)		0 (0)	0 (0)	
Introduction of COVID-19 Vaccine Dose 2 into Subcutaneous Tissue, Percutaneous Approach, New Technology Group 6	0 (0)	0 (0)		0 (0)	0 (0)	
Introduction of COVID-19 Vaccine into Subcutaneous Tissue, Percutaneous Approach, New Technology Group 6	0 (0)	0 (0)		0 (0)	0 (0)	
Visit: Inpatient Encounter	49064 (26.4)	56475 (26.2)	0.00	42183 (25.9)	42738 (26.2)	0.01
Visit: Short Stay	3603 (1.9)	2853 (1.3)	0.05	2603 (1.6)	2630 (1.6)	0.00
Visit: Inpatient Non-acute	3521 (1.9)	1515 (0.7)	0.11	1981 (1.2)	1504 (0.9)	0.03
SARS-CoV-2 (COVID-19) Vaccine	10225 (5.5)	12960 (6)	0.02	9134 (5.6)	9334 (5.7)	0.01

eTable 8. Baseline Characteristics for Block 8 Before and After Matching+A1:G117

	Before matching			After matching		
	Cohort, No. (%)			Cohort, No. (%)		
Characteristic Name	COVID-19	ORI	SMD	COVID-19	ORI	SMD
Total number	346000	264837		248869	248869	
Current Age, mean (SD), y	46.2 (18.8)	44.8 (20.7)	0.07	45.0 (19.0)	45.2 (20.6)	0.01
Age at Index, mean (SD), y	45.8 (18.8)	43.8 (20.7)	0.07	44.1 (19.0)	44.2 (20.6)	0.01
Gender	248257 (71.8)	191555 (72.3)	0.01	180013 (72.3)	179430 (72.1)	0.01
Female	228092 (65.9)	193787 (73.2)	0.16	179048 (71.9)	179006 (71.9)	0.00
Male	199376 (57.6)	160312 (60.5)	0.06	149845 (60.2)	148683 (59.7)	0.01
Unknown	135553 (39.2)	96758 (36.5)	0.05	91510 (36.8)	92620 (37.2)	0.01
Ethnicity	65458 (18.9)	51578 (19.5)	0.01	47321 (19)	48317 (19.4)	0.01
Hispanic or Latino	64431 (18.6)	33651 (12.7)	0.16	33823 (13.6)	33470 (13.4)	0.00
Not Hispanic or Latino	44404 (12.8)	30714 (11.6)	0.04	29409 (11.8)	29896 (12)	0.01
Unknown	32285 (9.3)	21704 (8.2)	0.04	21535 (8.7)	21122 (8.5)	0.01
Race	11071 (3.2)	7767 (2.9)	0.02	7514 (3)	7566 (3)	0.00
Asian	7611 (2.2)	5619 (2.1)	0.01	5571 (2.2)	5469 (2.2)	0.00
Black or African American	1013 (0.3)	791 (0.3)	0.00	756 (0.3)	756 (0.3)	0.00
Native American or Alaska Native	449 (0.1)	275 (0.1)	0.01	262 (0.1)	272 (0.1)	0.00
Native Hawaiian or Other Pacific Islander	105019 (30.4)	77452 (29.2)	0.02	73859 (29.7)	72800 (29.3)	0.01
Unknown	82770 (23.9)	67984 (25.7)	0.04	62612 (25.2)	61777 (24.8)	0.01
White	88287 (25.5)	65025 (24.6)	0.02	62151 (25)	61230 (24.6)	0.01
Hypertensive diseases	77669 (22.4)	54722 (20.7)	0.04	52839 (21.2)	52072 (20.9)	0.01
Other anxiety disorders	63391 (18.3)	47909 (18.1)	0.01	45230 (18.2)	44496 (17.9)	0.01
Neoplasms	67575 (19.5)	45842 (17.3)	0.06	44333 (17.8)	43655 (17.5)	0.01
Overweight and obesity	50676 (14.6)	43575 (16.5)	0.05	39209 (15.8)	38802 (15.6)	0.00
Depressive episode	50623 (14.6)	32830 (12.4)	0.07	32258 (13)	31585 (12.7)	0.01
Other forms of heart disease	41648 (12)	28809 (10.9)	0.04	27701 (11.1)	27197 (10.9)	0.01
Asthma	25110 (7.3)	20850 (7.9)	0.02	19105 (7.7)	18883 (7.6)	0.00
Type 2 diabetes mellitus	21187 (6.1)	19338 (7.3)	0.05	16968 (6.8)	16875 (6.8)	0.00
Nicotine dependence	24994 (7.2)	17610 (6.6)	0.02	16908 (6.8)	16688 (6.7)	0.00
Generalized anxiety disorder	16664 (4.8)	15337 (5.8)	0.04	13317 (5.4)	13186 (5.3)	0.00
Bronchitis, not specified as acute or chronic	17550 (5.1)	13786 (5.2)	0.01	12832 (5.2)	12619 (5.1)	0.00
Reaction to severe stress, and adjustment disorders	20559 (5.9)	11892 (4.5)	0.07	11815 (4.7)	11594 (4.7)	0.00
Other chronic obstructive pulmonary disease	14222 (4.1)	10118 (3.8)	0.01	9839 (4)	9620 (3.9)	0.00
Major depressive disorder, recurrent	12148 (3.5)	10867 (4.1)	0.03	9677 (3.9)	9571 (3.8)	0.00

© 2023 Olaker VR et al. *JAMA Network Open.*

Persons with potential health hazards related to socioeconomic and psychosocial circumstances	13532 (3.9)	7428 (2.8)	0.06	7484 (3)	7232 (2.9)	0.01
Attention-deficit hyperactivity disorders	12194 (3.5)	7426 (2.8)	0.04	7295 (2.9)	7117 (2.9)	0.00
Fatty (change of) liver, not elsewhere classified	10554 (3.1)	6067 (2.3)	0.05	6033 (2.4)	5851 (2.4)	0.00
Cerebral infarction	8334 (2.4)	6276 (2.4)	0.00	5959 (2.4)	5833 (2.3)	0.00
Certain disorders involving the immune mechanism	10233 (3)	5721 (2.2)	0.05	5781 (2.3)	5549 (2.2)	0.01
Hypertensive chronic kidney disease	9507 (2.7)	5571 (2.1)	0.04	5536 (2.2)	5418 (2.2)	0.00
Other specified diseases of liver	7858 (2.3)	5721 (2.2)	0.01	5451 (2.2)	5321 (2.1)	0.00
Other psychoactive substance related disorders	6910 (2)	6414 (2.4)	0.03	5430 (2.2)	5484 (2.2)	0.00
Phobic anxiety disorders	7453 (2.2)	5080 (1.9)	0.02	4830 (1.9)	4857 (2)	0.00
Bipolar disorder	5746 (1.7)	4467 (1.7)	0.00	4249 (1.7)	4199 (1.7)	0.00
Emphysema	6870 (2)	4248 (1.6)	0.03	4164 (1.7)	4080 (1.6)	0.00
Other rheumatoid arthritis	7260 (2.1)	3807 (1.4)	0.05	3910 (1.6)	3732 (1.5)	0.01
Psoriasis	5411 (1.6)	3839 (1.5)	0.01	3710 (1.5)	3646 (1.5)	0.00
Type 1 diabetes mellitus	5999 (1.7)	3577 (1.4)	0.03	3492 (1.4)	3439 (1.4)	0.00
Cannabis related disorders	6210 (1.8)	3379 (1.3)	0.04	3417 (1.4)	3285 (1.3)	0.00
Malignant neoplasms of lymphoid, hematopoietic and related tissue	4784 (1.4)	3548 (1.3)	0.00	3317 (1.3)	3283 (1.3)	0.00
Schizophrenia, schizotypal, delusional, and other non-mood psychotic disorders	3792 (1.1)	2329 (0.9)	0.02	2327 (0.9)	2240 (0.9)	0.00
Personal history of other mental and behavioral disorders	2389 (0.7)	2714 (1)	0.04	2055 (0.8)	2034 (0.8)	0.00
Opioid related disorders	2240 (0.6)	2263 (0.9)	0.02	1856 (0.7)	1834 (0.7)	0.00
Fibrosis and cirrhosis of liver	2870 (0.8)	1835 (0.7)	0.02	1800 (0.7)	1762 (0.7)	0.00
Bronchiectasis	2901 (0.8)	1610 (0.6)	0.03	1604 (0.6)	1576 (0.6)	0.00
Unspecified chronic bronchitis	3287 (1)	1530 (0.6)	0.04	1567 (0.6)	1507 (0.6)	0.00
Systemic lupus erythematosus (SLE)	2303 (0.7)	1583 (0.6)	0.01	1512 (0.6)	1521 (0.6)	0.00
Other stimulant related disorders	1722 (0.5)	2040 (0.8)	0.03	1493 (0.6)	1503 (0.6)	0.00
Unspecified dementia	1776 (0.5)	1371 (0.5)	0.00	1277 (0.5)	1261 (0.5)	0.00
Rheumatoid arthritis with rheumatoid factor	1851 (0.5)	1158 (0.4)	0.01	1133 (0.5)	1094 (0.4)	0.00
Simple and mucopurulent chronic bronchitis	1369 (0.4)	853 (0.3)	0.01	817 (0.3)	798 (0.3)	0.00
Chronic passive congestion of liver	1383 (0.4)	806 (0.3)	0.02	802 (0.3)	768 (0.3)	0.00
Cocaine related disorders	1306 (0.4)	812 (0.3)	0.01	775 (0.3)	782 (0.3)	0.00

© 2023 Olaker VR et al. *JAMA Network Open.*

Dementia in other diseases classified elsewhere	1222 (0.4)	759 (0.3)	0.01	751 (0.3)	733 (0.3)	0.00
Hepatic failure, not elsewhere classified	934 (0.3)	693 (0.3)	0.00	653 (0.3)	613 (0.2)	0.00
Inhalant related disorders	1162 (0.3)	662 (0.3)	0.02	648 (0.3)	635 (0.3)	0.00
Borderline personality disorder	1107 (0.3)	664 (0.3)	0.01	636 (0.3)	642 (0.3)	0.00
Alzheimer's disease	806 (0.2)	464 (0.2)	0.01	459 (0.2)	444 (0.2)	0.00
Portal hypertension	779 (0.2)	358 (0.1)	0.02	354 (0.1)	354 (0.1)	0.00
Sedative, hypnotic, or anxiolytic related disorders	570 (0.2)	379 (0.1)	0.01	352 (0.1)	353 (0.1)	0.00
Vascular dementia	420 (0.1)	257 (0.1)	0.01	251 (0.1)	247 (0.1)	0.00
Hallucinogen related disorders	128 (0)	73 (0)	0.01	69 (0)	70 (0)	0.00
Family history of other psychoactive substance abuse and dependence	114 (0)	62 (0)	0.01	63 (0)	60 (0)	0.00
Chronic hepatitis, not elsewhere classified	92 (0)	57 (0)	0.00	60 (0)	55 (0)	0.00
Dementia with Lewy bodies	178352 (51.5)	147920 (55.9)	0.08	136607 (54.9)	134544 (54.1)	0.01
Frontotemporal dementia	54993 (15.9)	37203 (14)	0.05	36128 (14.5)	35592 (14.3)	0.01
Antisocial personality disorder	103066 (29.8)	73968 (27.9)	0.04	71297 (28.6)	70442 (28.3)	0.01
Blood Pressure, Systolic	172115 (49.7)	143564 (54.2)	0.09	132534 (53.3)	130288 (52.4)	0.02
Blood Pressure, Systolic, At least 160 mm[Hg]	177742 (51.4)	147672 (55.8)	0.04	136225 (54.7)	134304 (54)	0.02
Blood Pressure, Systolic, 140-160 mm[Hg]	41535 (12)	25988 (9.8)	0.07	25590 (10.3)	25223 (10.1)	0.00
Blood Pressure, Systolic, At most 140 mm[Hg]	82640 (23.9)	57290 (21.6)	0.05	55579 (22.3)	54863 (22)	0.01
Blood Pressure, Diastolic	174790 (50.5)	145546 (55)	0.09	134477 (54)	132201 (53.1)	0.02
Blood Pressure, Diastolic, At least 100 mm[Hg]	151162 (43.7)	108418 (40.9)	0.13	105499 (42.4)	102253 (41.1)	0.03
Blood Pressure, Diastolic, 90-100 mm[Hg]	78067 (22.6)	50247 (19)	0.09	49786 (20)	48955 (19.7)	0.01
Blood Pressure, Diastolic, At most 90 mm[Hg]	71629 (20.7)	48930 (18.5)	0.06	47624 (19.1)	47114 (18.9)	0.01
BMI	64591 (18.7)	51713 (19.5)	0.02	48124 (19.3)	47304 (19)	0.01
BMI, At least 30 kg/m2	42711 (12.3)	27541 (10.4)	0.06	26707 (10.7)	26333 (10.6)	0.00
BMI, 25-30 kg/m2	34699 (10)	22078 (8.3)	0.06	21462 (8.6)	21103 (8.5)	0.01
BMI, At most 25 kg/m2	26984 (7.8)	20119 (7.6)	0.01	18782 (7.5)	18672 (7.5)	0.00
Hospital Inpatient Services	23891 (6.9)	17738 (6.7)	0.01	16554 (6.7)	16477 (6.6)	0.00
New or Established Patient Initial Hospital Inpatient Care Services	22438 (6.5)	16248 (6.1)	0.01	15288 (6.1)	15243 (6.1)	0.00

© 2023 Olaker VR et al. *JAMA Network Open.*

Severe acute respiratory syndrome coronavirus 2 (SARS-CoV-2) (Coronavirus disease [COVID-19]) vaccine, mRNA-LNP, spike protein, preservative free, 30 mcg/0.3mL dosage, diluent reconstituted, for intramuscular use	11108 (3.2)	7117 (2.7)	0.03	6888 (2.8)	6736 (2.7)	0.00
Immunization administration by intramuscular injection of severe acute respiratory syndrome coronavirus 2 (SARS-CoV-2) (Coronavirus disease [COVID-19]) vaccine, mRNA-LNP, spike protein, preservative free, 30 mcg/0.3mL dosage, diluent reconstituted; first dose	5081 (1.5)	3176 (1.2)	0.02	3195 (1.3)	3072 (1.2)	0.00
Immunization administration by intramuscular injection of severe acute respiratory syndrome coronavirus 2 (SARS-CoV-2) (Coronavirus disease [COVID-19]) vaccine, mRNA-LNP, spike protein, preservative free, 30 mcg/0.3mL dosage, diluent reconstituted; second dose	4747 (1.4)	2883 (1.1)	0.03	2922 (1.2)	2826 (1.1)	0.00
Critical Care Services	4516 (1.3)	2726 (1)	0.03	2777 (1.1)	2680 (1.1)	0.00
Severe acute respiratory syndrome coronavirus 2 (SARS-CoV-2) (Coronavirus disease [COVID-19]) vaccine, mRNA-LNP, spike protein, preservative free, 100 mcg/0.5mL dosage, for intramuscular use	486 (0.1)	431 (0.2)	0.01	401 (0.2)	394 (0.2)	0.00
Immunization administration by intramuscular injection of severe acute respiratory syndrome coronavirus 2 (SARS-CoV-2) (Coronavirus disease [COVID-19]) vaccine, mRNA-LNP, spike protein, preservative free, 100 mcg/0.5mL dosage; first dose	478 (0.1)	426 (0.2)	0.01	394 (0.2)	389 (0.2)	0.00
Immunization administration by intramuscular injection of severe acute respiratory syndrome coronavirus 2 (SARS-CoV-2) (Coronavirus disease [COVID-19]) vaccine, mRNA-LNP, spike protein, preservative free, 100 mcg/0.5mL dosage; second dose	889 (0.3)	200 (0.1)	0.04	253 (0.1)	200 (0.1)	0.01

© 2023 Olaker VR et al. *JAMA Network Open.*

Severe acute respiratory syndrome coronavirus 2 (SARS-CoV-2) (coronavirus disease [COVID-19]) vaccine, DNA, spike protein, adenovirus type 26 (Ad26) vector, preservative free, 5x10^10 viral particles/0.5mL dosage, for intramuscular use	132 (0)	59 (0)	0.01	58 (0)	57 (0)	0.00
Immunization administration by intramuscular injection of severe acute respiratory syndrome coronavirus 2 (SARS-CoV-2) (coronavirus disease [COVID-19]) vaccine, DNA, spike protein, adenovirus type 26 (Ad26) vector, preservative free, 5x10^10 viral particles/0.5mL dosage, single dose	36 (0)	17 (0)	0.00	19 (0)	16 (0)	0.00
Renal Transplantation Procedures	54 (0)	14 (0)	0.01	18 (0)	14 (0)	0.00
Liver Transplantation Procedures	10 (0)	0 (0)	0.01	10 (0)	0 (0)	0.01
Introduction of COVID-19 Vaccine into Muscle, Percutaneous Approach, New Technology Group 6	15 (0)	10 (0)	0.00	10 (0)	10 (0)	0.00
Introduction of COVID-19 Vaccine Dose 1 into Muscle, Percutaneous Approach, New Technology Group 6	0 (0)	0 (0)		0 (0)	0 (0)	
Introduction of COVID-19 Vaccine Dose 1 into Subcutaneous Tissue, Percutaneous Approach, New Technology Group 6	0 (0)	0 (0)		0 (0)	0 (0)	
Introduction of COVID-19 Vaccine into Subcutaneous Tissue, Percutaneous Approach, New Technology Group 6	0 (0)	0 (0)		0 (0)	0 (0)	
Introduction of COVID-19 Vaccine Dose 2 into Muscle, Percutaneous Approach, New Technology Group 6	0 (0)	0 (0)		0 (0)	0 (0)	
Severe acute respiratory syndrome coronavirus 2 (SARS-CoV-2) (coronavirus disease [COVID-19]) vaccine, DNA, spike protein, chimpanzee adenovirus Oxford 1 (ChAdOx1) vector, preservative free, 5x10^10 viral particles/0.5mL dosage, for intramuscular use	10 (0)	0 (0)	0.01	0 (0)	0 (0)	

© 2023 Olaker VR et al. *JAMA Network Open.*

Immunization administration by intramuscular injection of severe acute respiratory syndrome coronavirus 2 (SARS-CoV-2) (coronavirus disease [COVID-19]) vaccine, DNA, spike protein, chimpanzee adenovirus Oxford 1 (ChAdOx1) vector, preservative free, 5x10^10 viral particles/0.5mL dosage; first dose	91265 (26.4)	69914 (26.4)	0.00	65269 (26.2)	64393 (25.9)	0.01
Immunization administration by intramuscular injection of severe acute respiratory syndrome coronavirus 2 (SARS-CoV-2) (coronavirus disease [COVID-19]) vaccine, DNA, spike protein, chimpanzee adenovirus Oxford 1 (ChAdOx1) vector, preservative free, 5x10^10 viral particles/0.5mL dosage; second dose	8044 (2.3)	4778 (1.8)	0.04	4782 (1.9)	4669 (1.9)	0.00
Introduction of COVID-19 Vaccine Dose 2 into Subcutaneous Tissue, Percutaneous Approach, New Technology Group 6	3199 (0.9)	1281 (0.5)	0.05	1388 (0.6)	1279 (0.5)	0.01
Visit: Inpatient Encounter	29564 (8.5)	17478 (6.6)	0.07	17470 (7)	17199 (6.9)	0.00
Visit: Short Stay	6841 (2)	3775 (1.4)	0.04	3751 (1.5)	3732 (1.5)	0.00
Visit: Inpatient Non-acute	3200 (0.9)	1284 (0.5)	0.05	1385 (0.6)	1280 (0.5)	0.01
SARS-CoV-2 (COVID-19) Vaccine	29938 (8.8)	18453 (7.1)	0.06	18053 (7.4)	18095 (7.4)	0.00

© 2023 Olaker VR et al. *JAMA Network Open.*
