# Supplementary material for: Association of Recent SARS-CoV-2 Infection With New-Onset Alcohol Use Disorder, January 2020 Through January 2022
Source: JAMA Netw Open. 2023 Feb 9;6(2):e2255496. doi: 10.1001/jamanetworkopen.2022.55496 (PMC9912133; doi:10.1001/jamanetworkopen.2022.55496)
Supplement: Supplement 2. — Data Sharing Statement [file jamanetwopen-e2255496-s002.pdf]

Data Sharing Statement

Olaker. Association of Recent SARS-CoV-2 Infection With New-Onset Alcohol Use Disorder, January 2020 Through January 2022. *JAMA Netw Open*. Published February 09, 2023. doi:10.1001/jamanetworkopen.2022.55496

Data
Data available: No

Additional Information
Explanation for why data not available: We used a cloud-based database and cannot download the data set. In addition, the database is constantly being upgraded with new information, so the actual data from which the analysis was done will not be available at a subsequent time. That is why we indicate when the database was accessed and which specific data set was used. The EMR data is de-identified and so individual data cannot be made available to us or anyone else.
